# A Test of the Coordinated Expression Hypothesis for the Origin and Maintenance of the *GAL* Cluster in Yeast

**DOI:** 10.1371/journal.pone.0025290

**Published:** 2011-09-22

**Authors:** Gregory I. Lang, David Botstein

**Affiliations:** Department of Molecular and Cellular Biology and The Lewis-Sigler Institute for Integrative Genomics, Princeton University, Princeton, New Jersey, United States of America; Duke University, United States of America

## Abstract

Metabolic gene clusters—functionally related and physically clustered genes—are a common feature of some eukaryotic genomes. Two hypotheses have been advanced to explain the origin and maintenance of metabolic gene clusters: coordinated gene expression and genetic linkage. Here we test the hypothesis that selection for coordinated gene expression underlies the clustering of *GAL* genes in the yeast genome. We find that, although clustering coordinates the expression of *GAL1* and *GAL10*, disrupting the *GAL* cluster does not impair fitness, suggesting that other mechanisms, such as genetic linkage, drive the origin and maintenance metabolic gene clusters.

## Introduction

**Figure 1:**
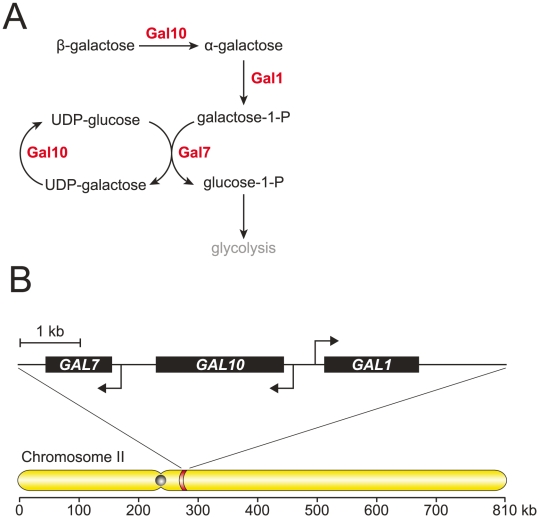
The *GAL1*-*GAL10*-*GAL7* gene cluster in *Saccharomyces cerevisiae*. (A) *GAL1*, *GAL10*, and *GAL7* encode enzymes that catalyze sequential steps in the assimilation of galactose. Gal1 is the galactokinase. Gal10 contains two catalytic domains: a mutarotase that interconverts galactose enantiomers, and an epimerase domain that converts UDP-galactose to UDP-glucose. Gal7 is the galactose-1-p uridyl transferase. An intermediate in galactose assimilation, galactose-1-p, is toxic to cells. (B) *GAL1*, *GAL10*, and *GAL7* are clustered within a 7 kb region on Chromosome II with *GAL1* and *GAL10* sharing a divergent promoter.

In eukaryotic genomes functionally related genes are, to a first approximation, dispersed throughout the genome. There are counter examples, however, of the physical clustering of genes whose products function in the same metabolic pathway [1]–[9]. In the yeast, *Saccharomyces cerevisiae*, metabolic gene clusters exist for biotin synthesis [2], allantoin degredation [9], and galactose assimilation [6]. The *GAL* cluster consists of three genes (*GAL1*, *GAL10*, and *GAL7*), encoding enzymes that catalyze four sequential steps in galactose assimilation, that are clustered in a 7 kb region of Chromosome II (Figure 1). The *GAL* cluster evolved independently through gene relocation in two fungal phyla (Ascomycota and Basidiomycota) and has been horizontally transferred within Ascomycota [6].

It is not clear what evolutionary forces favored the formation and maintenance of gene clusters. As is the case for hypotheses for the origin and maintenance of bacterial operons, there are two attractive ideas: coordinated expression [10] and genetic linkage [11]–[14]. Many gene clusters encode for metabolic pathways with toxic intermediates, for example thalianol and thalian-diol in the triterpene biosynthesis pathway in plants [1], [4], glyoxylate in the yeast allantoin degradation pathway [9], and galactose-1-phosphate in the yeast *GAL* pathway. Coordinated expression of individual enzymes of these pathways could facilitate metabolic channeling and lessen the buildup of toxic intermediates. Alternatively, the physical proximity of functionally related genes could reflect selection for genetic linkage, either to maintain alleles of co-adapted genes or as a result of recurrent horizontal transfer of the gene cluster. Neither of these models has been tested experimentally. Using the *GAL* cluster in *S. cerevisiae*, we directly test the coordinated expression hypothesis, which makes two experimental predictions: (1) clustering contributes to coordination gene expression and (2) clustering provides a fitness advantage.

## Results

**Figure 2:**
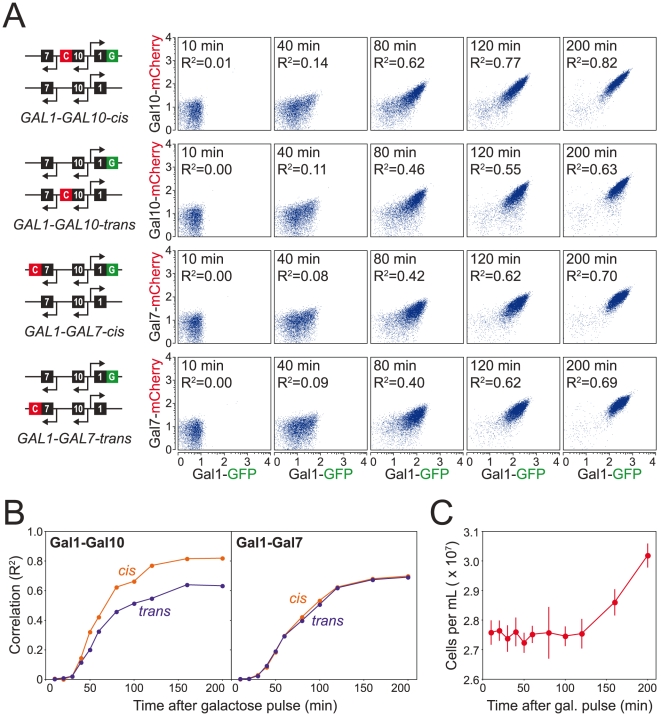
To determine the effect of gene clustering on the coordinated expression of the *GAL* genes, we monitored production of Gal1-GFP and Gal10-mCherry (or Gal7-mCherry) in both the *cis* and *trans* conformations following a 2.5 g/L galactose pulse into a steady-state glucose-limited (0.8 g/L) chemostat[**25**]. GFP and mCherry were quantified by flow cytometry. (A) Population profiles showing the correlation (R^2^) between Gal1-GFP and Gal10-mCherry (or Gal7-mCherry) following the galactose pulse. (B) Correlation coefficients (R^2^) between Gal1-GFP and Gal10-mCherry as a function of time following the galactose pulse. Gal1-GFP and Gal10-mCherry are more correlated in the *cis* conformation (0.82 at 200 min) compared to the *trans* conformation (0.63 at 200 min). For Gal1-GFP and Gal7-mCherry, however, we find no difference in the coordination of gene expression between the two conformations (0.70 and 0.69 for *cis* and *trans*, respectively at 200 min). The correlation between Gal1 and Gal7 in either conformation is similar to Gal1-Gal10 in the *trans* conformation. (C) The average cell density (± one standard deviation) for all eight populations following the galactose pulse as measured by Coulter counter. Although the Gal proteins were detectable 30 minutes, cell number did not increase until 120 minutes subsequent to the galactose pulse.

To determine whether the *GAL* cluster organization improves coordinated expression of the *GAL* genes, we generated diploid strains in which GFP is fused to *GAL1* and mCherry is fused to *GAL10* (or *GAL7*) in either the *cis* or *trans* conformation (Figure 2). We monitored the correlation between Gal1-GFP and Gal10-mCherry (or Gal7-mCherry) following induction of the *GAL* genes in a steady-state glucose-limited chemostat (Figure 2). Consistent with the coordinated expression hypothesis, Gal1-GFP and Gal10-mCherry are more correlated when these genes are in *cis*. This is not surprising since these two genes share a divergent promoter. For Gal1-GFP and Gal7-mCherry, however, we find no difference in the coordination of gene expression between the two conformations. The correlation between Gal1 and Gal7 in either conformation is similar to Gal1-Gal10 in the *trans* conformation. This is inconsistent with the hypothesis that the physical association of these genes facilitates coordinated expression.

**Figure 3:**
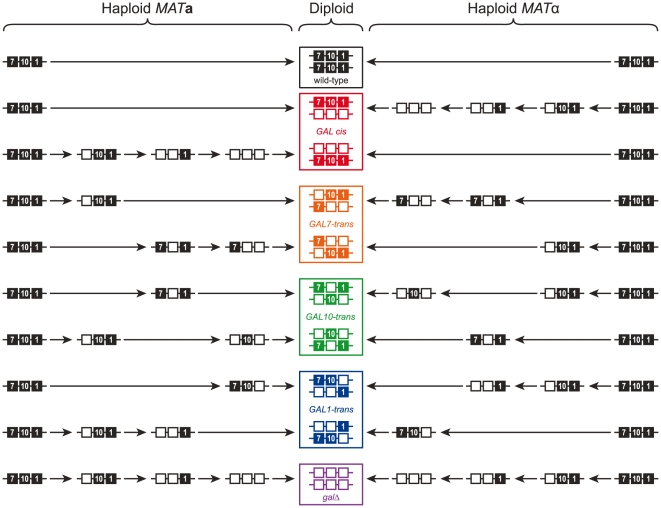
Strategy for disrupting the contiguity of the *GAL* cluster starting from the prototrophic S288c strains DBY12000 (*MAT*a) and DBY12001 (*MAT*α). Construction of strains hemizygous for each of the three *GAL*-cluster genes required three rounds of transformation replacing *GAL7*, *GAL10*, and *GAL1* with *HphMX*, *KanMX*, and *NatMX*, respectively. Prior to mating, the haploid strains were backcrossed to DBY12000 (or DBY12001) carrying either GFP or dTomato in order to fluorescently label strains for the competition experiment. Note that each of the four possible hemizygous was constructed twice independently, and are indicated by open and closed circles in Figure 4.

**Figure 4:**
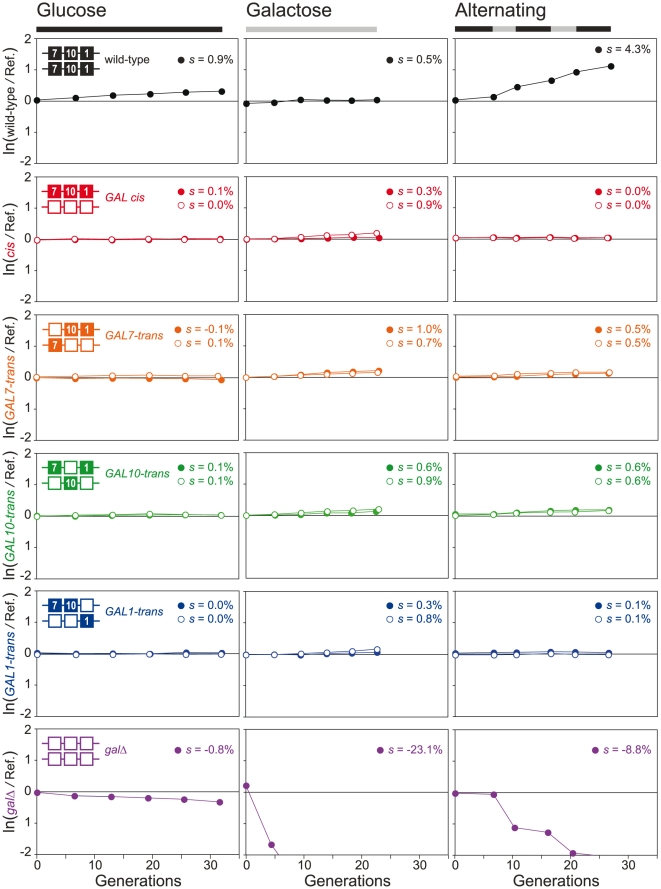
To determine if *GAL* gene clustering provides a selective advantage, we generated GFP-labeled strains hemizygous for *GAL1*, *GAL10* and *GAL7*, in either the *cis* conformation, or with one of the *GAL* genes in *trans* (**Figure 3**) and measured their fitness by competing each against a dTomato-labeled *GAL cis* strain in three conditions: glucose (YPD), galactose (YPG), and alternating glucose and galactose. Open and filled circles represent independently constructed biological replicates of the hemizygous strains (Figure 3). In glucose there is no difference in fitness for any of the hemizygous strains. The homozygous *GAL* (wild-type) strain has a 0.9% advantage and the homozygous *gal*Δ strain has a 0.8% fitness disadvantage compared to the hemizygous reference strain. Since the *GAL* genes are repressed in glucose, this fitness difference is due to the presence of the three drug cassettes (*KanMX*, *NatMX*, and *HphMX*) absent from the wild-type strain, present in one copy in the reference strain, and in two copies in the *gal*Δ strain. In galactose, the *gal*Δ strain is quickly outcompeted, however all other strains have a slight advantage over the reference strain, suggesting a slight advantage to GFP over dTomato in galactose (0.7±0.3%). The lack of a fitness difference between wild-type and the hemizygous strains in galactose suggests two things: (1) the cost of the drug markers in mitigated in galactose (perhaps because of slower growth in galactose and/or because the *GAL* genes are overexpressed under this condition and may incur a cost themselves), and (2) the hemizygotes are able to maintain adequate levels of the Gal proteins despite a two-fold reduction in gene copy number. Under alternating conditions, the wild-type strain has a 4.3% advantage indicating that increased copy number of the *GAL* genes, which does not affect fitness when growing exclusively in galactose, is beneficial in a changing environment, likely by establishing steady state levels of the Gal proteins more rapidly. In the alternating regime, like in galactose, disrupting the contiguity of the *GAL1*-*GAL10*-*GAL7* cluster does not impair fitness; *GAL10*-*trans* and *GAL7*-*trans* may, in fact, have a fitness advantage.

To determine whether *GAL* gene clustering provides a selective advantage, we generated strains hemizygous for *GAL1*, *GAL10* and *GAL7*, in either the *cis* conformation, or with one of the *GAL* genes in *trans* (Figure 3). We measured the fitness of the hemizygous strains, as well as homozygous wild-type and *gal*Δ strains in batch culture under three conditions: glucose (*GAL* genes fully repressed), galactose (*GAL* genes fully induced), and alternating glucose/galactose. Disrupting the contiguity of the *GAL1*-*GAL10*-*GAL7* cluster does not decrease fitness in any of the tested conditions (Figure 4). We have reported previously that the error in measurement in competitive fitness assays is approximately 0.4% [15]. The eight hemizygotes, competed in glucose, where we do not expect a difference in fitness, have a standard deviation of only 0.1%. Estimates of the effective population size for natural yeast populations are ∼10^7^ [16], [17]; therefore, evolution can conceivably act on selection coefficients several orders of magnitude smaller that we can detect in the laboratory. For this reason we can not rule out that very small, but non-trivial, selective forces play some role in the maintenance of the *GAL* cluster, although our data suggest that *GAL10*-*trans* and *GAL7*-*trans* may, in fact, have a slight fitness *advantage* in alternating glucose/galactose (0.5% and 0.6%, respectively) perhaps by alleviating transcriptional interference between *GAL10* and *GAL7* [18], [19]. These results fail to support the hypothesis that selection for coordinated gene expression is responsible for the origin or maintenance of the *GAL* gene cluster, and suggest (1) that the *GAL* cluster may be maintained in spite of fitness cost and (2) that coordinated expression of Gal1 and Gal10 is a consequence, rather than a cause, of clustering.

## Discussion

Our demonstration that disrupting the contiguity of the *GAL* cluster does not incur a fitness cost lends support to the hypothesis that genetic linkage is the selective force driving the origin and maintenance of *GAL* gene clusters. Why would genetic linkage of *GAL1*, *GAL10*, and *GAL7* be selectively advantageous? In *S. kudriavzevii*, a closely related species to *S. cerevisiae*, the *GAL* cluster, as well as the unlinked *GAL2*, *GAL4*, and *GAL80* exist as degenerate pseudogenes that are maintained, along with functional alleles of these genes, despite historical gene flow between the Gal^+^ and Gal^-^ subpopulations [20]. In a population maintaining the *GAL* genes as a balanced unlinked gene network polymorphism, linkage of *GAL1*, *GAL10*, and *GAL7* prevents the buildup of the toxic galactose-1-phosphate, which occurs in *GAL1*-proficient strains lacking either *GAL10* or *GAL7*. The loss of the *GAL* genes in *S. kudriavzevii* is far more recent than the evolution of the *GAL* cluster; however, it is not unique: at least five Ascomycota species have recently lost or pseudogenized the *GAL* genes [6], [21]. It is possible that the spread of nonfunctional *gal* genes in an ancient population drove the evolution of the *GAL* gene cluster. In a population segregating functional and nonfunctional alleles of unlinked *GAL* genes, the alleles of these genes will assort randomly in the absence of galactose. Upon exposure to galactose, Dobzhansky-Muller incompatibilities will be revealed between the functional *GAL1* allele and the nonfunctional *gal10* and *gal7* alleles. Clustering eliminates this incompatibility by genetically linking *GAL1*, *GAL10*, and *GAL7*. Similarly, the rate of loss of the *GAL* genes is greater in species where *GAL1*, *GAL10*, and *GAL7* are clustered [6]; this is consistent with the spread of nonfunctional alleles being attenuated in species with unlinked *GAL* genes.

A second mechanism that could favor genetic linkage is horizontal gene transfer. Phylogenic evidence indicates that fungal gene clusters, including the *GAL* cluster, can be horizontally transferred [5]–[7], [22]–[24]. The “selfish-operon” hypothesis posits that clustering enhances the spread of genes without producing a direct fitness benefit [11], [12], [14]. Given only one documented horizontal transfer of the *GAL* cluster [6], it is unclear if the rate of horizontal gene transfer is sufficient to explain the maintenance of the *GAL* cluster based solely on this mechanism.

We have shown that clustering coordinates the expression of *GAL1* and *GAL10*. Clustering, however, does not coordinate the expression of *GAL1* and *GAL7*, nor does it provide a fitness advantage during continuous induction or alternating induction and repression of the *GAL* genes. Our results fail to support the coordinated expression hypothesis and suggest that other mechanisms, such as genetic linkage, drive the origin and maintenance of *GAL* gene clusters in yeast.

## Materials and Methods

### Strain construction

All strains in this experiment are derived from the prototrophic S288c strains DBY12000 (*MAT* **a**) and DBY12001 (*MAT*α). Strains for monitoring the correlation between Gal1-GFP and Gal10-mCherry (or Gal7-mCherry) were constructed as follows: In DBY12000, GFP (with a *KanMX* marker) was fused to the *GAL1* gene and mCherry (with a *NatMX* marker) was fused to the GAL10 (or GAL7) gene. These strains were then crossed to DBY12001 to generate the Gal1-Gal10-*cis* and Gal1-Gal7-*cis* strains, respectively. Additionally, mCherry (with a *NatMX* marker) was fused to the GAL10 (or GAL7) gene in DBY12001. These strains were then crossed to DBY12000 (with a Gal1-GFP fusion) to generate the Gal1-Gal10-*trans* and Gal1-Gal7-*trans* strains, respectively.

Our strategy for disrupting the contiguity of the *GAL* cluster is shown in Figure 3. Starting with DBY12000 and DBY12001, we constructed all possible combinations of *gal1*Δ, *gal10*Δ, and *gal7*Δ, replaced with *NatMX*, *KanMX*, and *HphMX*, respectively. Prior to mating, the haploid strains were backcrossed to DBY12000 (or DBY12001) carrying either GFP or dTomato integrated at the dubious ORF *YLR255c* (marked with the *NatMX*) cassette in order to fluorescently label strains for the competition experiment.

### Coordinated gene expression measurements

We monitored production of Gal1-GFP and Gal10-mCherry or Gal7-mCherry (in either the *cis* or *trans* conformation) following a 2.5 g/L galactose pulse into a steady-state glucose-limited (0.8 g/L) chemostat [25]. Samples were taken at 10, 20, 30, 40, 50, 60, 80, 100, 120, 160, and 200 minutes following the galactose pulse and expression of Gal1-GFP and Gal10-mCherry (or Gal7-mCherry) was determined by flow cytometry. Correlation coefficients were calculated in Matlab and points on the axes were excluded.

### Fitness assays

We measured the fitness of the hemizygous strains, as well as homozygous wild-type and *gal*Δ strains in three conditions: glucose (*GAL* genes fully repressed), galactose (*GAL* genes fully induced), and alternating glucose/galactose. Fitness assays were performed as described previously [15] with slight modifications. Briefly, prior to mixing, cells were initially grown to mid log in YPD (for the glucose and alternating regimes) or YPG (for the galactose regime) prior to starting the competition. Cultures were diluted every 12 hours; dilutions from YPD and YPG were approximately 1∶500 and 1∶100, respectively, although the exact dilutions were adjusted to keep the cells per culture consistent between competitions. At each dilution, cells were counted to determine the number of generations between each sample point, and fitness was calculated as the rate of change of the *ln* ratio of experimental to reference versus generations [26].

### Notebook

The complete laboratory notebook describing these experiments is available as Notebook S1.

## Supporting Information

Notebook S1 **The complete laboratory notebook detailing the strain constructions and experiments presented in this study.**
(PDF)
