# Supplementary material for: A Test of the Coordinated Expression Hypothesis for the Origin and Maintenance of the GAL Cluster in Yeast
Source: PLoS One. 2011 Sep 22;6(9):e25290. doi: 10.1371/journal.pone.0025290 (PMC3178652; doi:10.1371/journal.pone.0025290)
Supplement: Notebook S1 — The complete laboratory notebook detailing the strain constructions and experiments presented in this study. (PDF) [file pone.0025290.s001.pdf]

8/27/08

OPERON-LIKE ORGANIZATION OF THE GAL GENES

Although eukaryotes lack true operons, there are examples of operon-like gene clusters. Three examples are the galactose utilization genes in S. cerevisiae (GAL1, GAL10, GAL7), the allantoin degradation genes in S.c. (DAL1, DAL2, DAL3, DAL4, DAL7, DCG1), and the thaliandiol synthesis genes in Arabadopsis (THAS, THAH, THAS):

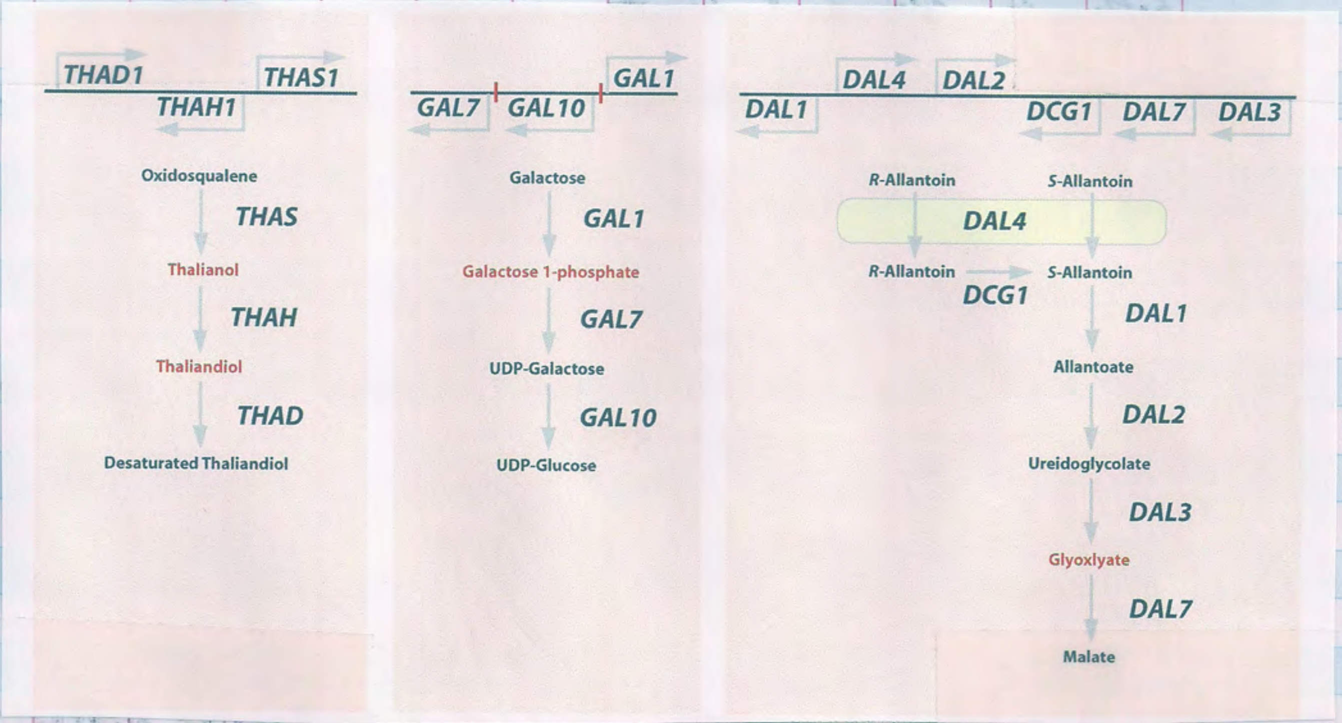

Two explanations have been given to account for this organization: genetic ~~clustering~~ linkage and metabolic channeling.

The genetic linkage hypothesis seems to be favored in the literature. It is interesting to note, however, that all three pathways above have intermediates that are toxic to the organism (in red). Here I want to test the hypothesis that the operon-like organization allows for better co-regulation of the genes and helps ~~channel~~ maintain flux through the pathway thus prevent the accumulation of the toxic intermediate ⟹

I want to show that disruption of the operon-like organization of the GAL genes:

(1) leads to less co-ordinated expression

(2) reduces fitness

(3) leads to a buildup of pathway intermediates.

The strategy for (1) & (2) is to tag the GAL genes with fluorescent proteins or delete the GAL genes both in cis and in trans.

I have put a lot of thought into how to do the strain construction. Some of the issues:

- strain bkg: W303 or S288c
- auxotrophs or prototrophs
- HAP1+ ?
- delete all 3 genes (GAL1, 10, 7) in each strain? or just pairs (i.e. GAL1 & 10)
- leave drug markers in place or popout? (Popout req. gal induction of Cre)
- Fitness assays: indirect (FACS) or direct (Sequencing)

8/28/08

<u>NOTES ON STRAINS</u>

I have decided upon a strain construction strategy. I will use prototrophic Hap1+ strains of S288c. FACS-based fitness assays will be more difficult since S288c has bkg fluorescence — also I can not use my standard (W303) reference. I would like to use quantitative sequencing to directly compare fitness of various strains. I am not going to popout the markers, rather I will use KanMX, NatMX, and HygMX for GAL1, 10, & 7. In addition, I will make sure that I delete all three genes in each strain. I will make the Mata & Matα portions of the strains separately — prior to mating I will select for $5FOA^R$ in each of the Matα strains — this will give each finished strain a unique SNP to be used for quantitative sequencing. This will require that the Mata portion have a deletion of ura3 to prevent amplification of the WT URA3.

8/28/08

STRAINS TO BE CONSTRUCTED — FOR GENE DELETION/FITNESS

1 - GAL1 K - KanMX
7 - GAL7 H - HygMX
10 - GAL10 N - NatMX

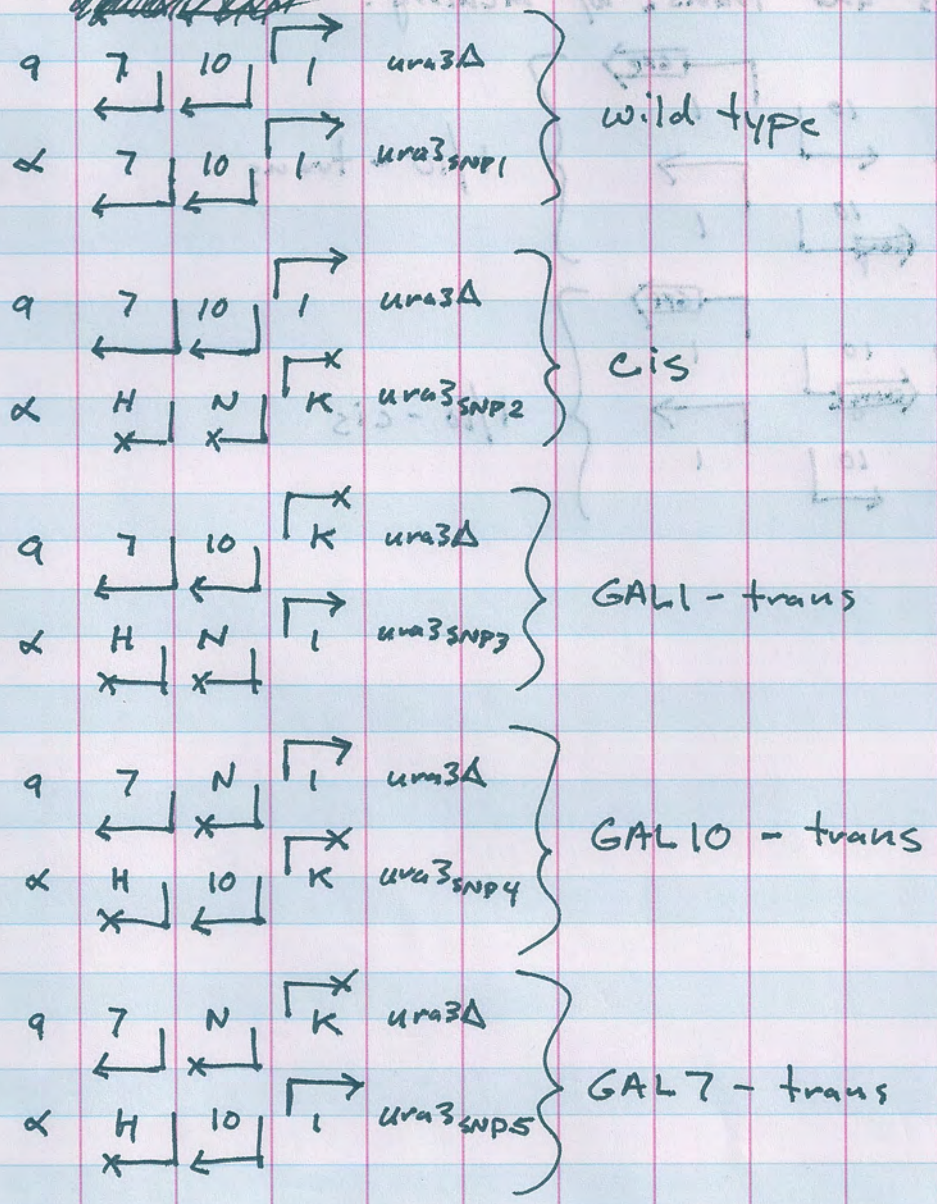

8/29/08

STRAINS TO BE CONSTRUCTED - CO-ORDINATED EXPRESSION

- In addition to measuring fitness in the deletion strains, I will need to construct strains to measure the coordinated expression. For this, I will use a GAL1-GFP fusion from Max. I will tag either GAL7 or GAL10 in both cis and trans w/ mCherry. For instance:

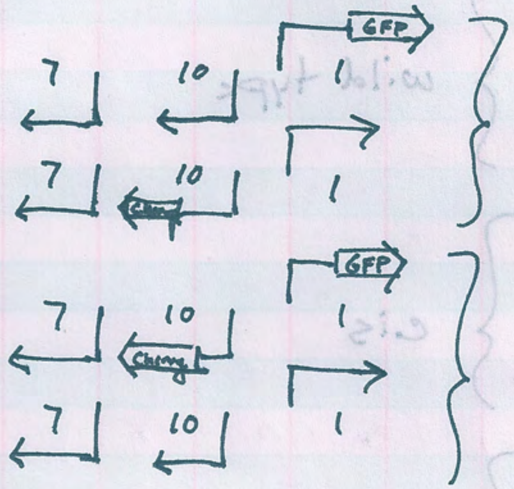

1/10 - trans

1/10 - cis

10/9/08

NOTES:

I've decided to construct the strains as URA prototrophs and I will plan to use the FACS-based fitness assay to measure growth rate against a common reference.

I have obtained primers to knock out GAL1, GAL10, & GAL7 using NatMX, KanMX, and HphMX, respectively:

oGIL103 GAL1_extF1 ATATACCTCTATACTTTAACGTCAAGGAGAAAAAACTATAGATCCCCGGGTTAATTAAGG
for knocking out GAL1 with NatMX from pAG25

oGIL104 GAL1_extR1 TGAGAAGTTGTTCTGAACAAAGTAAAAAAAAGAAGTATACTAATACGACTCACTATAGGG
for knocking out GAL1 with NatMX from pAG25

oGIL105 GAL10_extF1 CCATCCAAAAAAAAAGTAAGAATTTTTGAAAATTCAATATAACGTACGCTGCAGGTCGAC
for knocking out GAL10 with KanMX from pFA6a

oGIL106 GAL10_extR1 TATAGAGTGCATATTTTCAAGAAGGATAGTAAGCTGGCAAAATCGATGAATTCGAGCTCG
for knocking out GAL10 with KanMX from pFA6a

oGIL107 GAL7_extF1 TCAACATGATAAAAAAAAACAGTTGAATATTCCCTCAAAAGATCCCCGGGTTAATTAAGG
for knocking out GAL7 with HphMX from pAG32

oGIL108 GAL7_extR1 ATAGAAAAAATATGATATGAATGAATATTCCACTTTCTTTTAATACGACTCACTATAGGG
for knocking out GAL7 with HphMX from pAG32

oGIL109 GAL10mCherry_forward ACGGTGAAACTTACGGGTCCAAGATTGTCTACAGATTTTCCATGGTGAGCAAGGGCGAGG
for tagging GAL10 with mCherry::KanMX from pAC77

oGIL110 GAL10mCherry_reverse ATAGAGTGCATATTTTCAAGAAGGATAGTAAGCTGGCAAATAATACGACTCACTATAGGG
for tagging GAL10 with mCherry::KanMX from pAC77

oGIL111 GAL7mCherry_forward TAAGAAATTTAGATGGTCAGATTCATTATCTACAAAGACTGATGGTGAGCAAGGGCGAGG
for tagging GAL7 with mCherry::KanMX from pAC77

oGIL112 GAL7mCherry_reverse ATAGAAAAAATATGATATGAATGAATATTCCACTTTCTTTTAATACGACTCACTATAGGG
for tagging GAL7 with mCherry::KanMX from pAC77

Yesterday I struck from frozen stock bacteria carrying the plasmids pAG25, pFA6a, and pAG32.

This morning I inoculated a single colony off each plate into 2 mL 2×YT + Amp ~ 10:45

Miniprep plasmids → elute 30 λ EB

PCR cassettes:

TEMPLATE	PRIMERS
pAG25	GAL1_extF1 / GAL1_extR1
pFA6a	GAL10_extF1 / GAL10_extR1
pAG32	GAL7_extF1 / GAL7_extR1

10/10/08

TRANSFORMATION OF ~~[illegible]~~ FY4 & FY5 w/ gal7Δ::HphMX

On Tuesday (10/7) I streaked FY4 & FY5 from frozen stock

Last night I inoculated 3mL YPD w/ single colony of FY4 & FY5

This morning I diluted each o/N 1:100 (100λ into 10mL) ~ 9:15

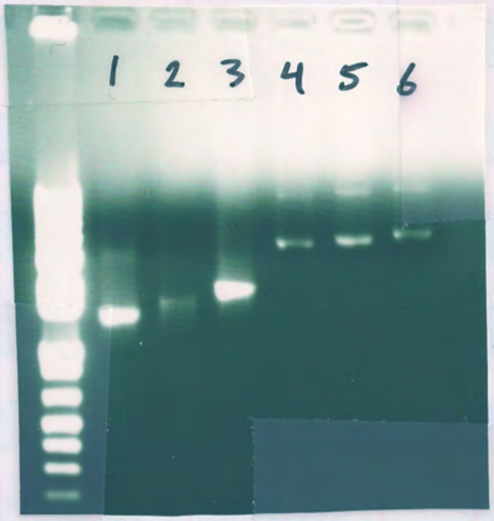

LANE

1	gal1Δ::Nat	primers on p. 5
2	gal10Δ::Kan	primers on p. 5
3	gal7Δ::Hph	primers on p. 5
4	pAG25	
5	pFA6a	
6	pAG32	

→ PCR prep → elute 30λ EB

Coulter count ~3:00 pm

Bkg	2.724E6	
FY4	4.192E7	4.222E7
FY5	3.947E7	3.968E7
Post Bkg	3.742E6	3.362E6

Transform FY4 & FY5 w/ 13λ of gal7Δ::Hph PCR product

10/13/08

NOTES ON gal7Δ::Hph TRANSFORMATION

- Transformation was done @ 30° → heat shock @ 42° w/ DMSO
- Plated on YPD. Incubated o/n then replica plated to YPD+hygromycin on Saturday (10/11).
- For the hygromycin plates I added 25 λ of 383 mg/mL hygro. stock ≈ 320 μg/mL final conc. I read that ~300 μg/mL is recommended. The hygro. was clearly not evenly distributed through the plate – I see lawns near the edges – next time I will add more volume to the plates to get a better spread of drug.

- Today I observed ~50 colonies on both of the transformation plates and no colonies on either of the control plates.
- Today I picked two single putative transformants off of each plate and inoculated 5 mL YPD. I also picked a single colony of FY4 & FY5 off of the plate from the fridge. Tomorrow I will transform these six strains with gal10Δ::KanMX → today I will need to redo the PCR in order to get enough product for 6 transformations.

- I decided to do all strain constructions in both mating-types. The strain construction strategy is quite lengthy; by doing it in both mating types I will have done the entire construction twice. If needed I can cross pairs of isogenic strains to make sure that there are no segregating differences in fitness.

10/14/08

TRANSFORMATION OF STRAINS w/ gal10Δ::KanMX

· Yesterday I started o/N cultures of
- FY4
- FY4 gal7Δ A
- FY4 " B
- FY5
- FY5 gal7Δ A
- FY5 " B

· ~ 9:15 - dilute cultures 1:100 (100 λ into 10 mL YPD)

· Yesterday I ran 2 200λ PCRs of the gal10Δ::KanMX cassette using either the pFA6a-KanMX plasmid or the purified gal10Δ::KanMX PCR product (see p 6) as template

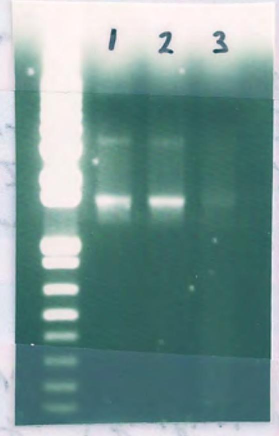

LANE
1 Purified PCR from p. 6
2 using pFA6a as template
3. using PCR as template

→ Purify lane 2 → elute 60λ and combine w/ lane 1 ⟹

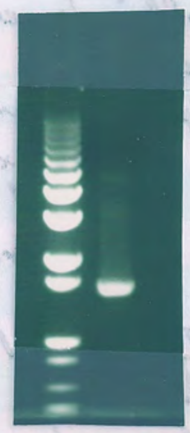

· 2:45 Coulter count

Bkg	2.8 E6	2.482 E6
FY4 gal7Δ A	4.174 E7	4.334 E7
Post Bkg	4.474 E6	4.8 E6

· 3:00 - Start transformation

10/15/08

NOTES ON gal10Δ::KanMX TRANSFORMATIONS

For the gal10Δ::KanMX transformations I did not do controls for no PCR product. This is partly because I am doing more transformations and I don't want to do twice the samples. It was only for the hygromycin plates where I was concerned about background. This way I can use more cells per transformation

→ At the end of the protocol I resuspended the cells using only 20λ (instead of 70λ) LiOAc Mix.

Yesterday I added 75λ H_2O + 23λ Hygromycin (383 mg/mL) to four G418 plates

Today I replica plated the YPD plates from yesterday onto either YPD+G418 (for the FY4 & FY5 transformations) and YPD+G418+hyg (for the transformations in the gal7Δ::HphMX bkgs).

10/16/08

I guess my G418 plates were too old ~2 Mo. I got a lawn on all plates. Today I poured a new batch of YPD plates. I will supplement these w/ 2x G418 and hygro (as needed) and redo the replica plating.

10/17/08

Replica plate the original YPD plates (from 10/14) onto the newly made drug plates.

~~10/15/08~~ 10/16/08

VERIFICATION AND PURIFICATION OF gal7::Hph TRANSFORMANTS

· On 10/14, before I transformed the strains I streaked the O/N cultures of the putative transformants to YPD to get single colonies to freezedown.
↳ Today I will start O/N cultures and freeze down tomorrow.
In addition ~~&~~ I streaked onto YPD & YPGalactose:

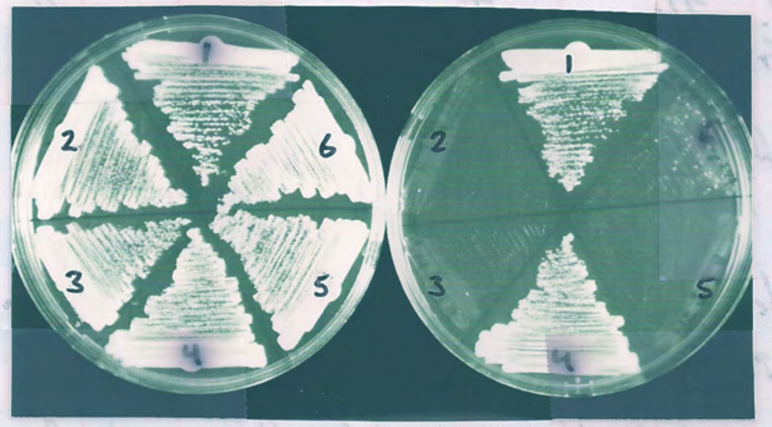

1 FY4
2 FY4 gal7Δ A
3 FY4 " B
4 FY5
5 FY5 gal7Δ A
6 FY5 " B

It appears that the transformations were successful
The few colonies on YPGal (especially in 6) could be due to not purifying the putative transformants.

10/16/08

PREP. OF pAC77 AND PCR OF mCHERRY CASSETTES AND gal1Δ::Nat

· Yesterday I miniprepped 2 clones carrying pAC77 off of the same LBAmp plate – A & B below

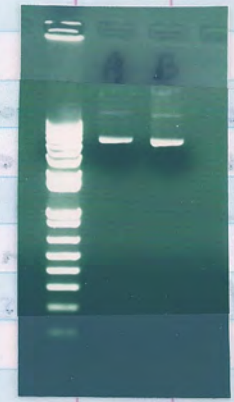

← It is hard to tell, but the purified plasmids may not be identical in size

I set up 5 PCRs (extension 3 m)

PRIMERS (see p 5)	TEMPLATE	LANE
GAL1extF1 / GAL1extR1	pAG25 (p. 6)	1
GAL10mCherry Forward/Reverse	pAC77 A	2
GAL7mCherry Forward/Reverse	" A	4
GAL10 —— " ——	" B	3
GAL7 —— " ——	" B	5

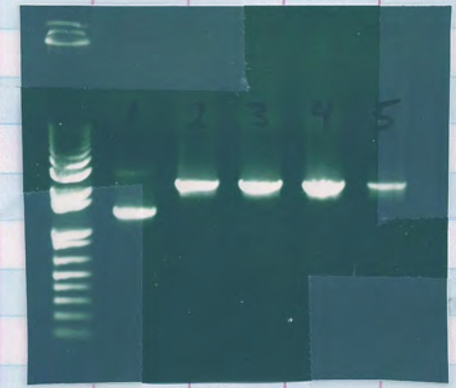

10/17/08

· Purify lane 1 above
↳ elute ~~30~~ 60λ EB and combine w/ lane 1 (p. 6) → I now have ~90λ of gal1Δ::KanMX ⇒

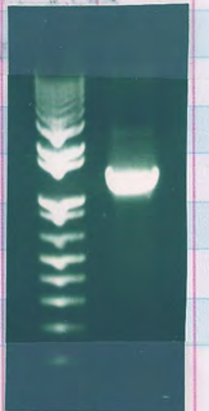

10/17/08

NOTE ON HAP1

I probably should have done this experiment in the HAP1+ background instead of the HAP1 hypomorphic background of FY4/FY5.

I think if I have trouble getting these last round of transformations to grow properly on the selective media, I may start over with the HAP1+ bkg. If I see colonies on Sunday I will continue with the strain construction in the FY4/FY5 bkg. Later I may need to address the issue since Max's GFP strain is HAP1+.

· Today I froze down the following strains:

FY4	gal7Δ::HphMX A	→	yGIL306
FY4	—— " —— B		" 307
FY5	—— " —— A		" 308
FY5	—— " —— B		" 309

· Today I streaked the following strains

yGIL306, " 307, " 308, " 309 } from O/N

" 294 (GAL1-GFP::Nat), " 201 (FY4), " 202 (FYS), ~~[illegible]~~ DBY 12000, ~~[illegible]~~ DBY 12001 } from frozen stock

10/20/08

NOTES ON gal10Δ::KanMX TRANSFORMATIONS

There are a number of colonies on the G418 & the G418+Hygromycin plates from 10/17 (see p. 9). There are ~100 colonies per plate — the colonies near the edge are smaller and the bkg lawn is more apparent probably due to a gradient in drug concentration — I restricted ~~pic~~ to picking colonies near the center of the plate.

From some plates I picked a single colony and inoculated 5 ml YPD o/N for transformation today. Today I purified 2 putatitive transformants off of each plate for freezedown and I streaked the o/Ns on YPD and YPGal

PLATE	putatitive gal10Δ		gal10Δ	10/23/08 - freezedown
FY4		→ o/N YPD →	A	yGIL310
		→	B	311
FY4 gal7ΔA	"	→ o/N YPD →	A	312
		→	B	313
" B	"	→ o/N YPD →	A	314
		→	B	315
FY5	"	→ o/N YPD →	A	316
		→	B	317
FY5 gal7ΔA	"	→ o/N YPD →	A	318
		→	B	319
" B	"	→ o/N YPD →	A	320
		→	B	321

10/23/08

YPD YPGal

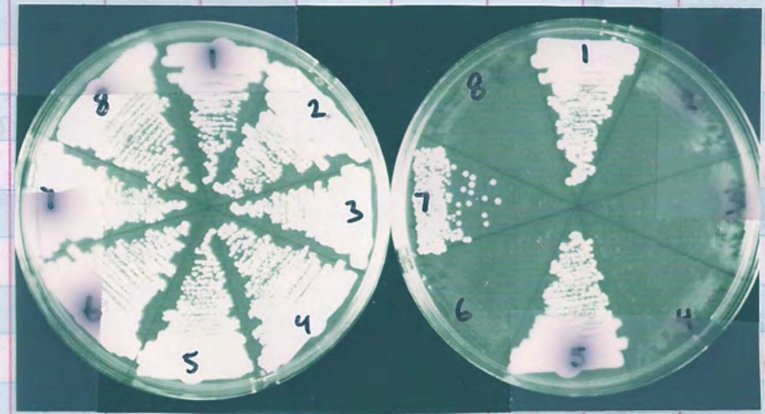

~~[illegible]~~	
1	FY4
2	FY4 gal10Δ A
3	FY4 gal7Δ A gal10Δ A
4	FY4 gal7Δ B gal10Δ A
5	FY5
6	FY5 gal10Δ A
7	FY5 gal7Δ A gal10Δ A
8	FY5 gal7Δ B gal10Δ A

10/20/08

TRANSFORMATIONS OF gal1Δ::NatMX AND mCherry FUSIONS

Today I am doing a large # of transformations

SET1: Transform the following strains w/ gal1Δ::NatMX cassette:

- FY4
- FY4 gal7Δ
- FY4 gal10Δ
- FY4 gal7Δ gal10Δ
- FY5
- FY5 gal7Δ
- FY5 gal10Δ
- FY5 gal7Δ gal10Δ

SET2: Transform yGIL294 (GAL1-GFP::NatMX) ~~[illegible]~~ and DBY12001 with:

- GAL10-mCherry::KanMX
- GAL7-mCherry::KanMX

Yesterday I started O/N cultures of the above strains. This morning I diluted all cultures 1:100 (100λ into 10mL) ~ 9:00am

Start transformations ~3pm

10/21/08

Today I replica plated the transformations from YPD to the proper single, double, or triple drug media

NOTES

- For the gal1Δ::NatMX transformations I used 20λ LiOAc mix; for the mCherry::KanMX transformations I used 70λ and split the cells into two transformations.

- Drug additions:

		STOCK CONC.
G418	→ 60λ	(200 mg/mL)
Nat	→ 30λ	(100 mg/mL)
Hyg	→ 23λ	(383 mg/mL)

- Prior to transformation I pooled the two GAL10-mCherry::KanMX and the two GAL7-mCherry::KanMX PCRs (see p. 11)

10/21/08

SOME DISCUSSION

Soon I will have the strains I need to start doing experiments. First I will need to cross the strains to produce the diploids described on p. 3&4 (note that I ended up using Nat for GAL1 and Kan for GAL10).
↳ For the deletion strains I can build two indep. sets since I built everything in both mating types

· Possible control exp — since everything is in both mating types I can cross isogenic strains to make sure that there are no segregating fitness differences.

· The 1st expt. will probably be the GFP/mCherry expt so that I can get a good idea of what Glucose/Galactose concentrations to use

· I will do all expts in ~~a mix~~ glucose, galactose, and a mixture of glucose & galactose

· It may be useful to do expression arrays on the heterozygous deletes to see if in fact the gene dosage is halved.

· If the fusions function properly I could look at co-localization of the GAL gene products — some evidence suggests that they may form a complex

· I could potentially propagate the trans-deletions for many transfers to see ~~th~~ if I can select for gene conversion.
↳ A similar experiment can be done in a haploid by moving one of the gal genes and seeing if I can select for restoration of the complex.

10/24/08

NOTES ON gal1Δ::NatMX and mCherry TRANSFORMATIONS

- I see many (~50) colonies on all plates except for the GAL1-GFP::NatMX transformed w/ mCherry::KanMX where there are no colonies — possibly the cells are not Nat^R.
  ↳ today I replica plated these YPD plates to 2×G418

- On many of the multi-drug plates ~~[illegible]~~ it was difficult to distinguish the true transformants over the bkg. Several things are probably contributory to this: drug gradients across the plate, different densities of cells due to replica plating, and drug interactions
  ↳ for each of the 10 successful transformations (see p. 14) I picked a single colony close to the center of the plate and restreaked to YPD (two colonies for the mCherry strains).

10/27/08

NOTES ON mCherry TRANSFORMATIONS

→ Today I see lawns on these G418 plates → Could Max have marked GAL1-GFP w/ KanMX? The strains he gave me are listed as Nat^R but I have never checked these strains. → Rep. to Nat plates.

Today I noticed a single colony colong on the GAL10-mCherry GAL1-GFP plate — this colony is near the edge where the drug concentration should be the lowest → Picked this colony; inoc. 5mL YPD ⇒ 10/28/08 Not red on Gal → discard

10/30/08

- Max did use KanMX — I checked in his thesis — also I ~~grew~~ streaked his strain (along w/ ~~a~~ known $G418^R$ and Nat^R strains) onto YPD, YPD+G418 & YPD+Nat and verified that it is KanMX. Today I will redo these transformations

- ~~[illegible]~~ Yesterday I checked the putative mCherry fusions for fluorescence. Both GAL7 and both GAL10 fusions (strains yGIL330-333, p 17) show strong fluorescence.

10/27/08

NOTES ON gal1Δ::NatMX TRANSFORMATIONS

- Today I picked a single colony off of each of the restreaked putative transformants → inoc. 5 mL YPD

~~Today~~

10/28/08

STRAINS		FREEZEDOWN
yGIL201 (FY4) putative gal1Δ::NatMX	→	yGIL322
yGIL202 (FY5) —— // ——	→	323
306 (FY4 gal7Δ::HphMX) —— // ——	→	324
308 (FY5 gal7Δ::HphMX) —— // ——	→	325
310 (FY4 gal10Δ::KanMX) —— // ——	→	326
316 (FY5 gal10Δ::KanMX) —— // ——	→	327
312 (FY4 gal7Δ::HphMX gal10Δ::KanMX) —— // ——	→	328
318 (FY5 gal7Δ::HphMX gal10Δ::KanMX) —— // ——	→	329
DBY12000 putative GAL10-mCherry::KanMX A	→	330
" —— // —— B	→	331
" putative GAL7-mCherry::KanMX A	→	332
" —— // —— B	→	333

10/30/08

PHENOTYPIC CHARACTERIZATION OF GAL MUTANT STRAINS

I wanted to phenotypically characterize the deletion and fusion strains that I have built thus far. Yesterday I spotted these strains onto YPD, YPD+hyg, YPD+Nat, YPD+G418, & YPGal. Some of the strains (7, 8, 11, 12, 13, 14, 15, 16, 19, 20, 21, & 22) I had as saturated cultures on the bench (see p. 17). The others I streaked from frozen stock on 10/27 — I picked a single 2 day old colony and resuspended it in 100λ YPD to do the spotting.

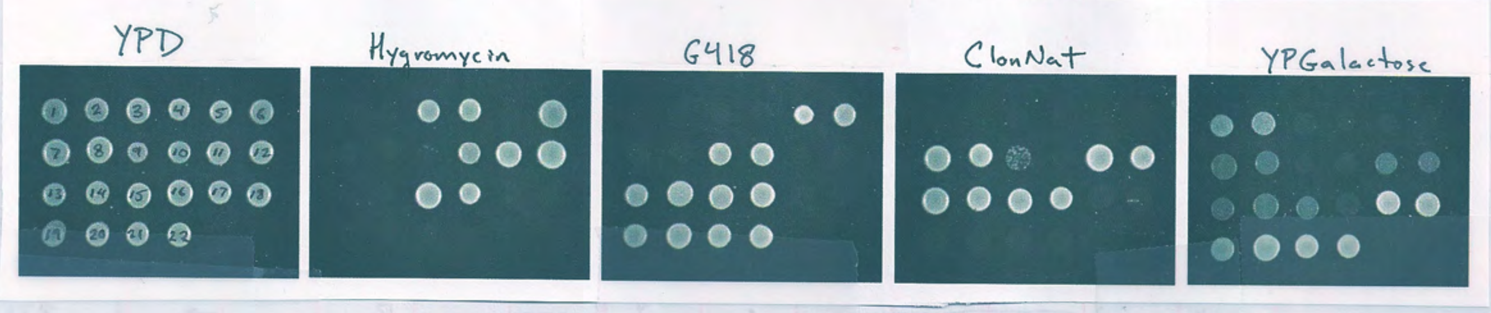

Spot	Strain	YPD	Hyg	G418	Nat	Gal
1	FY4	+	-	-	-	+
2	FY5	+	-	-	-	+
3	FY4 gal7	+	+	-	-	-
4	FY5 gal7	+	+	-	-	-
5	FY4 gal10	+	-	+	-	-
6	FY5 gal10	+	+	+	-	-
7	FY4 gal1	+	-	-	+	-
8	FY5 gal1	+	-	-	+	-
9	FY4 gal7 gal10	+	-	+	?	-
10	FY5 gal7 gal10	+	+	+	-	-
11	FY4 gal7 gal1	+	+	-	+	-
12	FY5 gal7 gal1	+	+	-	+	-
13	FY4 gal10 gal1	+	-	+	+	-
14	FY5 gal10 gal1	+	-	+	+	-
15	FY4 gal7 gal10 gal1	+	+	+	+	-
16	FY5 gal7 gal10 gal1	+	+	+	+	-
17	DBY12000	+	-	-	-	+
18	DBY12001	+	-	-	-	+
19	DBY12001 GAL7-mCherry A	+	-	+	-	+
20	DBY12001 GAL7-mCherry B	+	-	+	-	+
21	DBY12001 GAL10-mCherry A	+	-	+	-	+
22	DBY12001 GAL10-mCherry B	+	-	+	-	+

Incorrect Strains:

- #6 - FY5 gal10Δ should not grow on hyg
- #9 - FY4 gal7Δ gal10Δ should grow on hyg but not on Nat

10/30/08

TRANSFORMATION OF yGIL294 AND DBY12001 W/ mCherry FUSIONS

I need to redo my fusions using the NatMX marker

· I miniprepped two colonies off of a freshly streaked plate of bacteria carrying pAC80 (A & B below). This plasmid is the same as pAC77 (see p. 11) except that it has the NatMX reporter instead of KanMX.

· 200λ PCRs as before (p. 11)

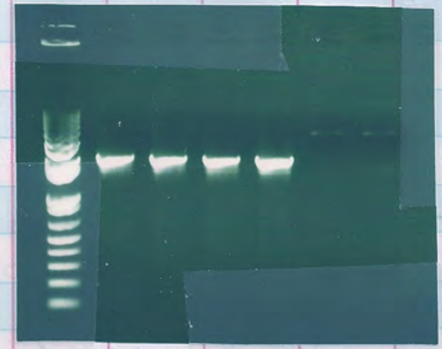

LANE	
1	GAL10-mCherry::Nat off pAC80 A
2	——— B
3	GAL7-mCherry::Nat off pAC80 A
4	——— B
5	pAC80 A
6	pAC80 B

→ PCR purify lanes 1 & 3 → elute 30λ EB

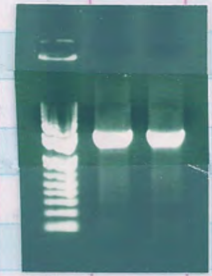

· Started o/N cultures last night → this morning dilute 1:100 (100λ into 10mL) → 9:15am

· 3:00 pm – Coulter count

Bkg	1.174E6	1.132E6
yGIL294	4.199E7	4.338E7
DBY12001	4.13E7	4.121E7
Post Bkg	2.248E6	1.692E6

· Start transformation ~ 3:15

10/31/08

NOTES ON TRANSFORMATIONS

- For yGIL294 & DBY12001 I resuspended the cells in 60λ LiOAc + 10λ carrier
this gave me ~120λ cells which I split into 2 60λ aliquots
and added 13λ of PCR product to each

· To 2 YPD plates I added 30λ ClonNat and to 2 plates
I added 30λ ClonNat + 60λ G418 (each 1000x)

· Today I replica plated the YPD plates to the respective
drug plates.

11/3/08

· Yesterday I picked two independent putative transformants
of each of the 4 transformations and restreaked to YPD.

· This morning I picked ~~a~~ another single colony off of each plate
and patched it on YPGalactose.

11/4/08

Yesterday afternoon I patched the 8 restreaked putative mCherry transformants
onto YPGal. This morning I looked at the cells under the scope
- of the 4 that I patched off of the transformation plate onto
YPGal only the yGIL294-gal7-mCherry was red - the other
3 were dark.
- All 8 of the ones restreaked to YPD were red. I picked a
single colony off of each of these plates and inoculated 5mL YPD -
I will freeze down these strains tomorrow.

11/5/08

FREEZE DOWN mCherry::NatMX FUSIONS

yGIL294	GAL10-mCherry	A	→ yGIL337
	〃	B	→ 338
	GAL7-mCherry	A	→ 339
↓	〃	B	→ 340
DBY12001	GAL10-mCherry	A	→ 341
	〃	B	→ 342
	GAL7-mCherry	A	→ 343
↓	〃	B	→ 344

11/7/08

CONSTRUCTION OF DIPLOID STRAINS (GFP & mCherry FUSIONS)

- On 11/4 I streaked yGIL294, DBY12000, & DBY12001 from freezer
- On 11/5 I patched the above ~~strains~~ strains (yGIL337-344) on YPD
- Yesterday I picked 3 zygotes of the following crosses after 3h on YPD:

	Cross		Genotype	Strain
(1)	yGIL337 ×	DBY12001	(GAL1-GFP/GAL10-mCherry cis) ⟹	yGIL345
(2)	yGIL338 ×	↓	〃	346
(3)	yGIL339 ×	↓	(GAL1-GFP/GAL7-mCherry cis)	347
(4)	yGIL340 ×	↓	〃	348
(5)	yGIL341 ×	yGIL294	(GAL1-GFP/GAL10-mCherry trans)	349
(6)	yGIL342 ×	↓	〃	350
(7)	yGIL343 ×	↓	(GAL1-GFP/GAL7-mCherry trans)	351
(8)	yGIL344 ×	↓	〃	352
(9)	DBY12001 ×	DBY12000		353

11/10/08

- Yesterday I started O/N 3mL YPD cultures of the 9 strains above → inoculated off of the ~~dissection~~ zygote plates.
- Today I froze down the strains (→ yGIL345–353 above)

11/7/08

EXPERIMENT: COORDINATED GENE EXPRESSION DURING GALACTOSE PULSE

I am setting up to test if the GAL gene organization coordinates expression of the GAL genes

PROTOCOL OUTLINE

- I will pulse in 0.25 g/L galactose into a steady state glucose limited chemostat (0.08 g/L glucose in feed)
- Samples will be taken every 20 min for 3 h. after pulse
- Strains:
  - 1/10 - cis
  - 1/10 - trans
  - 1/7 - cis
  - 1/7 - trans

  } all strains are GAL1-GFP and have mCherry fused to either GAL10 or GAL7 on either the same chromosome ~~a~~ (cis) or ~~[illegible]~~ homologous chromosome (trans) as GAL1-GFP

For crosses 1, 3, 5, & 7 ~~[illegible]~~ on p. 21, pick two colonies and inoculate 1 mL Glucose-limited chemostat media. Spot 5 λ onto YPD and YPGalactose. Add 4 additional mL of Gluc-limited media to each tube. → rollerdrum 5:00pm

11/8/08

- Inoculated Chemostats (see "Chemostat Experiments" notebook, Exp #4)

11/12/08

- Yesterday I patched onto YPD and YPGal the following:
  - yGIL294
  - ~~[illegible]~~ yGIL353

  } from fridge

  - yGIL341
  - yGIL343
  - yGIL349
  - yGIL351

  } from freezer

- pick large streak full of cells → ~~[illegible]~~ wash 1 mL H_2O → resuspend in 1 mL PBST

  ↳ these will be controls for FACS

11/12/08

GALACTOSE PULSE EXPERIMENT

· Pulse in 375λ 20% Galactose (200g/L) into each 300 mL chemostat ⟹ pulse = 0.25 g/L

Sample (time following pulse) counts

t(min)	1/10 cis	1/10 trans	1/7 cis	1/7 trans
-10	3.003 E7	2.715 E7	2.68 E7	2.75 E7
10	2.812 E7	2.721 E7	2.762 E7	2.737 E7
20	2.779 E7	2.803 E7	2.744 E7	2.731 E7
30	2.705 E7	2.793 E7	2.751 E7	2.701 E7
40	2.799 E7	2.802 E7	2.726 E7	2.713 E7
50	2.708 E7	2.763 E7	2.688 E7	2.734 E7
60	2.764 E7	2.784 E7	2.732 E7	2.726 E7
80	2.87 E7	2.77 E7	2.719 E7	2.669 E7
100	2.753 E7	2.783 E7	2.706 E7	2.739 E7
120	2.811 E7	2.774 E7	2.731 E7	2.7 E7
160	2.922 E7	2.852 E7	2.825 E7	2.841 E7
200	3.06 E7	3.043 E7	2.983 E7	2.987 E7
11/13/08 → 1200	2.475 E7	2.503 E7	2.484 E7	2.389 E7

11/13/2008

NOTES ON GALACTOSE PULSE EXPERIMENT

Sampling: at each time point I removed 500λ using the p1000. I took 10λ for coulter counting. I spun the remainder for 2 min, aspirated media and resuspended in 1mL of PBST (PBS + 0.1% tween) and put O/N in fridge

After the 200 min point I checked the fluorescence — I could see both GFP and mCherry in all strains (although the mCherry was faint)

→ Today I will run the samples through the FACS

From the counts above it appears that cell # increases only after 160 min — this is consistent w/ Max's data

11/14/08

PRELIMINARY ANALYSIS OF FACS DATA

Yesterday I ran the Gal-pulse timecourse samples through the FACS. I looked at the data in FlowJo, but I have yet to do a careful analysis — that will probably wait until next week. It appears that GAL1 & GAL10 expression are better coordinated when in cis. However I do not see any difference in the coexpression of GAL & GAL7 between the cis and trans configurations.

Some possible confounding factors:

(1) Maturation of the fluorescent reporters — I always see Gal1-GFP first — this could mean that GAL1 is turned on before GAL10 & GAL7 or that GFP matures faster — if ~~this~~ ~~is~~ the latter is true this would be problematic because the mCherry readout would actually reflect transcription/translation from earlier time points.

(2) Lack of CR in glucose-limited chemostats — carbon limited chemostats are in a state of "non-induction" for the GAL genes as opposed to "repressed" when in the presence of glucose. This ~~p~~ is a concern I had early on — I want to redo this experiment going from repressed to induced. There are several ways to achieve this both in batch and in chemostats.

- in chemostats (1) pulse in a mixture of glucose & galactose into a glucose-limited chemostat or (2) swich carboys from phosphate-limited glucose to phosphate-limited galactose
- in batch by (1) growing in a mixture of glucose and galactose or (2) by spinning down log-phase glucose cultures and resuspending in galactose.

12/20/08

CONSTRUCTING DIPLOID DELETION STRAINS

- On Tuesday (12/16) I streaked the following strains from the freezer:

yGIL 201	WT		
202	WT		
306	7Δ		
308	7Δ		
310	10Δ		
316	10Δ		
322	1Δ		
323	1Δ		
312	10Δ	7Δ	
318	10Δ	7Δ	
326	1Δ	10Δ	
327	1Δ	10Δ	
328	1Δ	10Δ	7Δ
329	1Δ	10Δ	7Δ

- On [illegible] Thursday (12/18) I started 5 mL o/n YPD cultures with a single colony of each strain (except 201 & 202).

- Yesterday I made genomic preps of the 12 strains, and ran PCR using the following primers:

39923958 IDT
G.LANG
81510977 10/21/2008
GAL1_extF2
5'- AAC GTC AAG GAG AAA AAA C -3'
Tm= 48.3 ºC , MW= 5,871.9
5.8 OD_{260} 28.00 nmol 0.16 mg

39923959 IDT
G.LANG
81509760 10/21/2008
GAL1_extR2
5'- ATT CAT ATA GAC AGC TGC CC -3'
Tm= 51.9 ºC , MW= 6,061.0
6.6 OD_{260} 34.10 nmol 0.21 mg

39923960 IDT
G.LANG
81509761 10/21/2008
GAL10_extF2
5'- AAT GAC AGC TCA GTT ACA AAG -3'
Tm= 50.6 ºC , MW= 6,447.3
5.7 OD_{260} 26.40 nmol 0.17 mg

39923961 IDT
G.LANG
81509762 10/21/2008
GAL10_extR2
5'- GTA AGC TGG CAA ATC AGG AA -3'
Tm= 53.0 ºC , MW= 6,199.1
6.3 OD_{260} 30.20 nmol 0.19 mg

39923962 IDT
G.LANG
81509763 10/21/2008
GAL7_extF2
5'- CCC TCA AAA ATG ACT GCT GA -3'
Tm= 52.9 ºC , MW= 6,070.0
6.2 OD_{260} 32.50 nmol 0.20 mg

39923963 IDT
G.LANG
81509764 10/21/2008
GAL7_extR2
5'- CCA CTT TCT TTT TAC AGT CTT -3'
Tm= 48.4 ºC , MW= 6,288.1
6.4 OD_{260} 35.00 nmol 0.22 mg

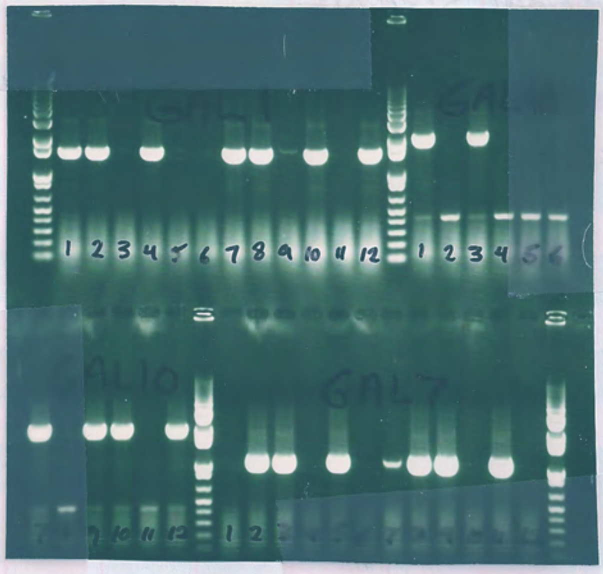

LANE	
1	yGIL306
2	310
3	322
4	312
5	326
6	328
7	308
8	316
9	323
10	318
11	327
12	329

- All primer pairs amplify the coding regions; therefore, no product will be formed in the Δ strains.

PRODUCT SIZES

GAL1	1.6 kb
GAL10	2.1 kb
GAL7	1.1 kb

Two of the lanes were ambiguous — I reran a few to make sure that the two light bands were not due to overloading adjacent wells

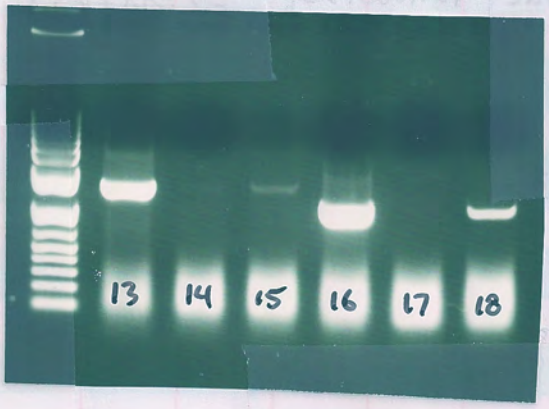

LANE	PRIMERS	TEMPLATE
13	GAL1	yGIL310
14	"	322
15	"	323
16	GAL7	310
17	"	306
18	"	308

12/20/08

SUMMARY OF PCR PHENOTYPING

Four strains appear to be incorrect:
(1) yGIL308 - should be 7Δ
(2) yGIL323 - — " — 1Δ
(3) yGIL318 - — " — 10Δ 7Δ
(4) yGIL329 - — " — 10Δ 7Δ 1Δ

} interestingly these are all Matα strains

I should redo these PCRs and get primers that are external to the coding sequence.

SETTING UP CROSSES

I wanted to make homozygous WT and deletion strains by crossing:

Mata		Matα	
yGIL201	×	yGIL202	WT / WT
yGIL328	×	yGIL329	7Δ10Δ1Δ / 7Δ10Δ1Δ —

In addition I wanted to make various heterozygous strains using the following crosses:

Mata		Matα		
yGIL201	×	yGIL329	WT / 7Δ10Δ1Δ	—
306	×	327	7Δ / 10Δ1Δ	
310	×	325	10Δ / 7Δ1Δ	
322	×	318	1Δ / 10Δ7Δ	—
312	×	323	7Δ10Δ / 1Δ	—
324	×	316	7Δ1Δ / 10Δ	
326	×	308	10Δ1Δ / 7Δ	—
328	×	202	7Δ10Δ1Δ / WT	

(marked crosses: likely to be wrong (see above))

Set up crosses (~9:30 am)
@ 12:30 - pick 3 zygotes for each cross

12/23/08

Yesterday I picked a single colony from each cross and inoculated 5ml YPD.

12/23/08

I reran the PCR on the two strains that were ambiguous (see p.). Here it seems that both yGIL323 & yGIL308 are true gal1Δ and gal7Δ respectively

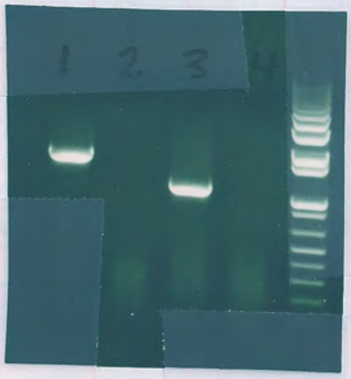

LANE	PRIMERS	TEMPLATE
1	GAL1	yGIL310
2	"	yGIL323
3	GAL7	yGIL310
4	"	yGIL308

NOTES ON STRAIN CONSTRUCTION

From the PCRs I suspect that two of my strains are incorrect: yGIL318 and yGIL329 (which was derived from yGIL318). I have 3 isogenic strains to yGIL318 (yGIL319-321, see p. 13). I have yet to do PCR on yGIL324 & 325 — I hope to do that today. Also, today I will phenotype these strains and the crosses.

I should have been wary of yGIL318 given the photograph on p. 13. showing some growth of this strain on Gal plates.

- Streak yGIL319-321 from freezer. Friday I plan to transform these strains w/ the ~~[illegible]~~ gal11Δ::NatMX cassette to generate a Mat α triple Δ to replace the incorrect ~~strains, yGIL365 and yGIL357~~ strain yGIL329.

12/24/08

· Yesterday I froze down the crosses from p. 27

Mat a	Mat α		New Strain #
201	× 202	→	yGIL 356
201	× 329	→	357
328	× 202	→	358
306	× 327	→	359
326	× 308	→	360
310	× 325	→	361
324	× 316	→	362
312	× 323	→	363
322	× 318	→	364
328	× 329	→	365

· Yesterday I made gDNA preps from yGIL324 & yGIL325. Overnight, I ran PCRs to genotype these two strains

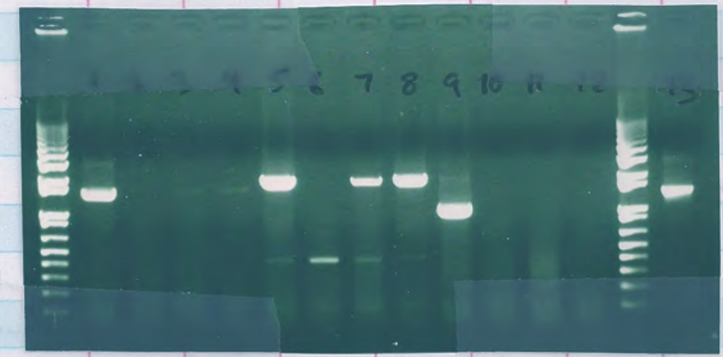

LANE	PRIMERS	TEMPLATE	CONTROLS
1	GAL1	yGIL 306	+
2	"	328	−
3	"	324	
4	"	325	
5	GAL10	306	+
6	"	328	−
7	"	324	
8	"	325	
9	GAL7	310	+
10	"	328	−
11	"	324	
12	"	325	

Both yGIL324 & 325 are gal1Δ/gal7Δ

· Yesterday I set up a 100λ PCR to amplify the gal1Δ::NatMX cassette from pAG25 − 3m extension

→ this morning I ran the PCR on a gel (lane 13 above) and put the PCR in the freezer.

12/24/08

PHENOTYPIC CHARACTERIZATION OF GAL Δ HAPLOIDS AND DIPLOIDS

Yesterday I pinned the haploid deletes and the diploids to YPD, YPGal, YPD+Hyg, YPD+G418, and YPD+ClonNat

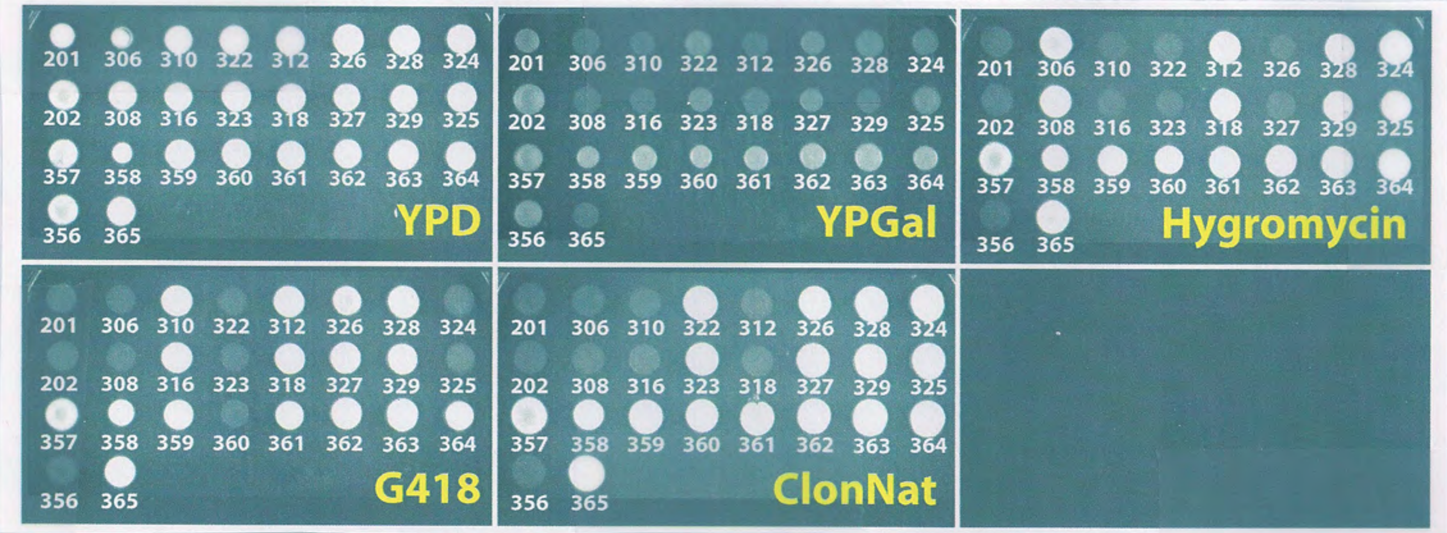

- the YPGal plate is slow growing since it is an old plate

Summary of Phenotypic assay

	YPD	YPGal	Hyg	G418	Nat
yGIL 201	+	+			
yGIL 306	+		+		
yGIL 310	+			+	
yGIL 322	+				+
yGIL 312	+		+	+	
yGIL 324	+		+		+
yGIL 326	+			+	+
yGIL 328	+		+	+	+
yGIL 202	+	+			
yGIL 308	+		+		
yGIL 316	+			+	
yGIL 323	+				+
yGIL 318	+		+	+	
yGIL 325	+		+		+
yGIL 327	+			+	+
yGIL 329	+		+	+	+
yGIL 356	+	+			
yGIL 357	+	+	+	+	+
yGIL 358	+	+	+	+	+
yGIL 359	+	+	+	+	
yGIL 360	+	+	+		+
yGIL 361	+	+	+	+	+
yGIL 362	+	+	+	+	+
yGIL 363	+	+	+	+	+
yGIL 364	+	+	+	+	+
yGIL 365	+		+	+	+

Mat a: WT (201); single Δs (306, 310, 322); Double Δs (312, 324, 326); Triple Δ (328)

Mat α: WT (202); Single Δs (308, 316, 323); Double Δs (318, 325, 327); Triple Δ (329)

Diploids (356–365)

12/26/08

Note: After 2 extra days of growth I have noticed a few strains growing where I did not expect to see growth:

On YPGal: Growth of 322, 324, 326, 328, 323, 325, & 327. Few small colonies on 306, 312 and 365.

On Hyg: What looks like a jackpot on 326 & 327

On Nat: ~~[illegible]~~ What looks like a jackpot on 312 & 316

12/24/08

SUMMARY OF INCORRECT STRAINS

yGIL318: did not genotype properly (p. 26) - should be 10Δ 7Δ but it only looks like 7Δ.

yGIL329: did not genotype properly (p. 26) → should be 10Δ7Δ1Δ but it only looks like 7Δ. this strain was derived from yGIL318.

Both yGIL318 & yGIL329 have the correct drug markers (p. 30)
↳ one possibility is that this strain duplicated the GAL1/10 region

yGIL357: derived from yGIL329

yGIL364: derived from yGIL318

yGIL365: derived from yGIL329

yGIL360: this is a bit of a puzzle. This strain is a cross between yGIL326 and yGIL308. Although I was, for a time, skeptical that yGIL308 is correct (see p. 26 & 27), I think that it is indeed (p. 28 & 30). The most plausible explanation is the spontaneous loss of KanMX.

12/27/09

MORE STRAIN PHENOTYPING

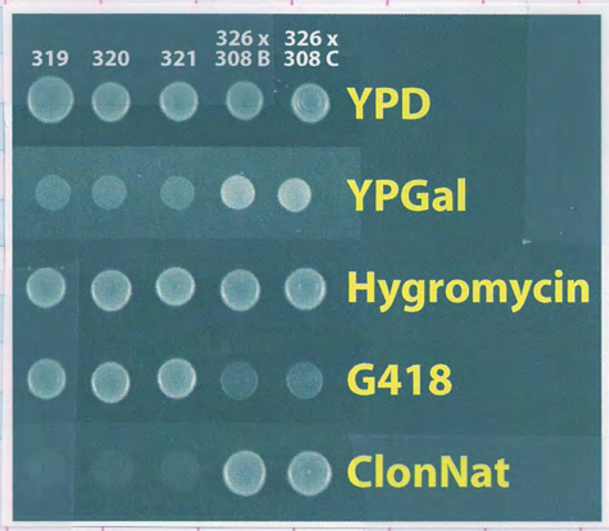

• I streaked from the freezer yGIL319-321 (supposedly isogenic to yGIL318; 10Δ7Δ) and transformed each w/ gal1Δ::NatMX (p.32)
↳ all 3 strains phenotype correctly

• I also phenotyped the two other $^{326}_{\times 308}$ zygotes I picked on 12/20 (p. 27)
↳ neither ~~[illegible]~~ phenotypes correctly (should be G418R) — I will need to redo this cross.

12/26/08

TRANSFORMATION OF yGIL319-321 w/ gal1Δ::NatMX

- Last night I started 5mL o/N cultures of yGIL319-321
- This morning I made 2 dilutions:
  - 1:100 – 100λ into 10 mL YPD } 10:45am
  - 1:50 – 200λ —"— }

Preparation of gal1Δ::NatMX cassette

- On 12/23 I ran a 100λ PCR of gal1Δ::NatMX (p. 29)
  ↳ today I PCR purified the cassette → elute in 40λ H_2O

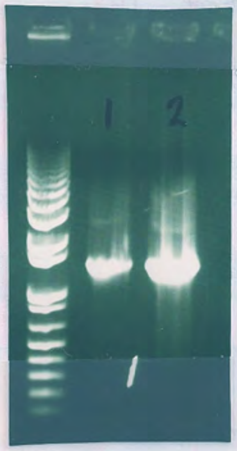

LANE

1 gal1Δ::NatMX new
2 —"— from 10/20 (p. 14) ~ I had ~2λ of that remaining on my bench.

5:45

Coulter count 1:100 dilutions & start transformation

~~Bkg~~
~~yGIL319~~
~~yGIL320~~
~~yGIL321~~
~~Post Bkg~~

Coulter counter not working

12/27/08

- Last night I overlaid G418, Nat, and Hyg onto 3 YPD plates (p. 14 for conc.)

- Today I replica plated the YPD plates from yesterday.

12/29/08

TRANSFORMATION CONT.

· I see a number of colonies on all 3 plates. Today I picked two putative gal1Δ::NatMx transformants of yGIL319-321. ⟶ restreak on YPD

GENOTYPING yGIL319-321 — primers same as p. 25

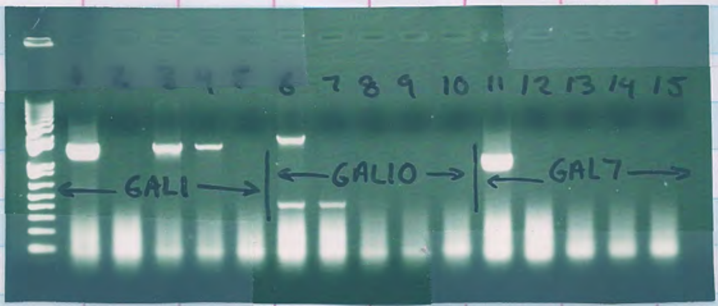

LANE

1, 6, & 11 - positive control (yGIL306 or yGIL310, see p. 29)

2, 7, & 12 - negative control (yGIL328)

3, 8, & 13 - yGIL319

4, 9, & 14 - yGIL320

5, 10, & 15 - yGIL321

⟶ yGIL321 did not genotype properly — should be GAL1.

12/31/08

· Last night I started 5 mL o/n cultures of the ~~putatitive~~ putative transformants
· Today I made gDNA preps and genotyped @ GAL1;

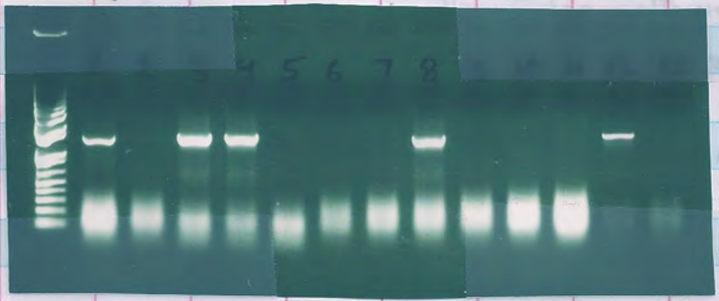

LANE		
1	yGIL306	- pos. control
2	328	- neg. control
3	319	
4	320	
5	321	
6	319	putative transformant A
7	319	—— " —— B
8	320	putative transformant A
9	320	—— " —— B
10	321	putative transformant A
11	321	—— " —— B
12	306	putative gal1 mutant
13	312	putative gal1 mutant

· Freezedown: yGIL319 putative transformant A ⟹ yGIL366
yGIL320 putative transformant B ⟹ yGIL367

1/2/09

· On 12/31 I streaked all 6 putative transformants onto YPD, YPGal, ~~#~~ G418, Nat, and Hyg. All 6 phenotyped correctly (although not all genotyped correctly)

12/29/08

NOTES ON PHENOTYPING

· On 12/26 I noted that after 2 days extra growth I could see strains ~~growing~~ on YPGal plates and drug plates where I didn't expect to see growth (p. 30)

· On 12/27 I took pictures of the plates:

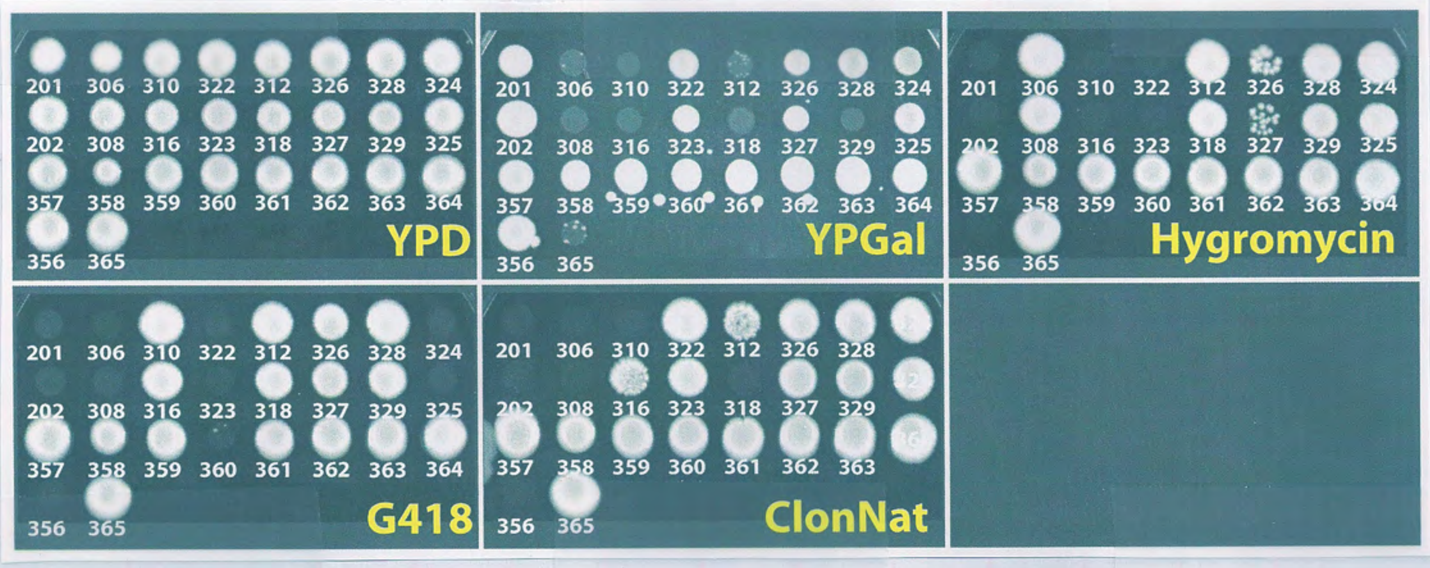

· I can explain the growth on YPGal:
  - After 1 day of growth, only the true Gal+ strains grow (p. 30)
  - After 3 days of growth, gal− strains can grow — presumably using a.a.s or ethanol as a carbon source.
  - However strains that have GAL1 but lack either GAL10 and/or GAL7 will suffer from galactose toxicity.
  - The ~~mutants~~ colonies I see growing on the ~~g~~ YPGal plates (on an otherwise dead bkg) are most likely GAL1 mutants

1/2/09

· On ~~[illegible]~~ 12/29, I picked a single colony from the yGIL306 & 312 spots on the YPGal plates and restreaked on YPD. On 12/30 I started a 5 mL o/n from a single colony and on 12/31 I made gDNA preps and amplified GAL1 (lanes 12 & 13 on p. 33)
↳ I suspect that both of these are GAL1 mutants — this is supported by the observation that no product was amplified from the yGIL312 putative gal1 mutant.

1/2/09

SECOND ROUND OF CROSSES

Today I set up a second round of crosses to generate strains that were incorrect in the 1st cross (p. 31). In addition I set up crosses to generate FACS reference strains for the fitness assays.

Mat a	Mat α
yGIL322×	yGIL319
322×	320
201×	366
201×	367
328×	366
328×	367
326×	308
214×	207
313 ~~214~~×	220 ~~213~~

- Strains yGIL366 & 367 were streaked on 12/31; strains yGIL319 & 320 were streaked on 12/23; all others were streaked on 12/29.

- For the yGIL214 × yGIL207 & yGIL220 × yGIL213, I set up two crosses using independent colonies of each — this is because I have had trouble with the fluorescent strains (particularly w/ GFP)

1/5/09

- This morning I inoculated 5 mL YPD w/ a single diploid colony off of eaches of the cross plates (except 326×308, 2 colonies; ~~213×220, 2 colonies~~ 214×207, 2 colonies per plate)

- This afternoon I spotted 5 λ of the gal mutant crosses onto YPD, YPGal, G418, Nat, and Hyg.

- Streak from freezer:

yGIL356 (WT)
358 (cis)
359 (7-trans)
361 (10-trans)
362 (10-trans)
363 (1-trans)

1/6/09

PHENOTYPING STRAINS FROM SECOND ROUND OF CROSSES

All 7 galΔ crosses phenotyped correctly – all are resistant to all 3 drugs and all but the 322×319 and 322×320 are Gal^{+}.

Freeze down crosses:

yGIL201 × yGIL366 (cis) → yGIL368
201 × 367 (cis) → 369
326 × 308 (7-trans) → 370
322 × 319 (1-trans) → 371
322 × 320 (1-trans) → 372
328 × 366 (homozyg. Δ) → 373
328 × 367 (homozyg. Δ) → 374

I examined all 6 of the fluorophore crosses under the scope last night and today (after overnight in the fridge in 2 vol. PBST). All appear to be equally fluorescent – previously the haploid GFP strains have appeared to lose fluorescence, but the diploids look fine under the scope – they may still not look great in the FACS – we'll see.

I froze down two GFP diploids & two dTomato diploids ⟷ because of a numbering mixup they are not sequential.

yGIL214 × yGIL207 → yGIL375 (homozyg. YLR255Δ::GFP NAT)
yGIL213 × yGIL220 → 376 (homozyg. YLR255Δ::dTomato Nat)
yGIL213 × yGIL220 → 377 ← isogenic (to 376)
yGIL214 × yGIL207 → 378 ← isogenic (to 375)

1/7/09

MORE GENOTYPING gal1Δ STRAINS

I have done PCR to verify the absence of GAL1, 10, & 7 in my Δ strains (p 25, 26, 29, & 33). Here I am using PCR to verify the presence/absence of the drug cassettes. I set up PCRs using the following primer pairs (standard PCR 25λ)

43024155 IDT
G.LANG
82587624 12/31/2008
GAL1_extF3 121
5'- AAA TGG AAA AGC TGC ATA ACC -3'
Tm= 51.8 °C , MW= 6,456.3
6.5OD260 30.00nmol 0.19mg

& NatMX_intR1 (oGIL006)

43024156 IDT
G.LANG
82587625 12/31/2008
GAL10_extF3 122
5'- ATG GGG CTC TTT ACA TTT CC -3'
Tm= 52.6 °C , MW= 6,074.0
7.0OD260 38.60nmol 0.23mg

& KanMXintR1 (oGIL005)

43024157 IDT
G.LANG
82587626 12/31/2008
GAL7_extF3 123
5'- TAC AGT GTT CAC AAA ATA GCC -3'
Tm= 50.6 °C , MW= 6,398.2
7.1OD260 33.90nmol 0.22mg

&

43024158 IDT
G.LANG
82587627 12/31/2008
HphMX_intR1 124
5'- GTG AGT TCA GGC TTT TTA CC -3'
Tm= 51.4 °C , MW= 6,114.0
7.4OD260 39.70nmol 0.24mg

MATa

MATα

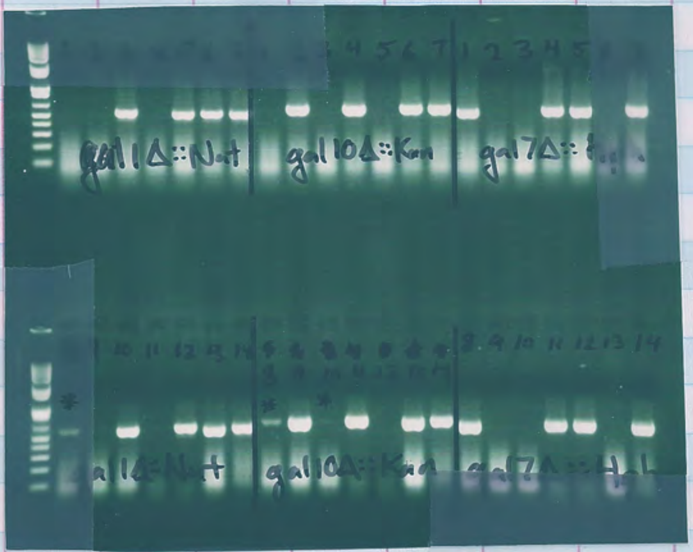

LANE		
1	yGIL306	7Δ
2	310	10Δ
3	322	1Δ
4	312	7Δ 10Δ
5	324	7Δ 1Δ
6	326	10Δ 1Δ
7	328	7Δ 10Δ 1Δ
8	yGIL308	7Δ
9	316	10Δ
10	323	1Δ
11	319	7Δ 10Δ
12	325	7Δ 1Δ
13	327	10Δ 1Δ
14	366	7Δ 10Δ 1Δ

* ambiguous bands - should not have a product if correct.

1/8/09

- from the genotyping yesterday, two strains (yGIL308 & yGIL323) produced ambiguous results (p.38, *)
- Today I reran the PCRs and found the same result:

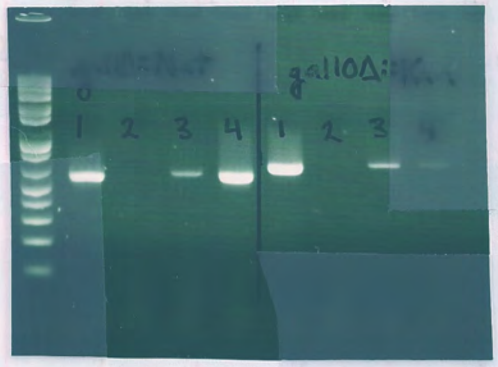

LANE		
1	yGIL328	(positive control)
2	306	(negative control)
3	308	
4	323	

- again I see light bands (not true positives) in yGIL308 (gal1 & gal10) and yGIL323 (gal10). Perhaps I should redo the gDNA preps.

1/7/09

EXPERIMENTAL PLAN

Now that I have constructed and verified my diploid deletion strains, I want to do fitness assays on these strains in 3 media: Glucose, Galactose, and Glucose/Galactose. Before I do the actual experiment I need to determine a few things: (1) What ~~concentration~~ ratio of Glucose to Galactose should I use in the mixed sugar media?, and (2) What dilutions should I do to keep the cells in exponential growth? I will run pilot experiments to address each of these questions.

PILOT EXPERIMENT 1: GLUCOSE/GALACTOSE MIXTURE

Like previous fitness assays (see "EVOLUTION 1" notebook) I will dilute each tube (a mixture of Exp. & Ref. strains) every 12 h. For the Gluc./Gal. mixed media I would like the switch in carbon source to occur near the 6 h. point. I don't think that the yeast exhaust the sugar by 12 h (I would guess that they use half of their sugar); therefore I would probably want Gluc:Gal ~ 1:4.

- Yesterday I made 0.5L of YPD & YPGal
- Yesterday I streaked yGIL345 (#GAL1-GFP, GAL10-mCherry, p 21) from freezer.
- Today I inoculated 5 ml of YPD & YPGal w/ an independent small colony of yGIL345 ~ rollerdrum ~ 5:00.

1/8/09

10:30am	Coulter count			to get to 10^6 cells / 10 mL
Bkg	88000	152000		
YPD	1.554E8	1.569E8	→	~~20~~ 7 λ into 10 mL
YPGal	4.945E7	4.983E7	→	200 λ into 10 mL
Post Bkg	4.28E6	2.202E6		

1/8/09

I made 30 mL of each of the following:
- 50% Dextrose, 50% Galactose (15 mL YPD & 15 mL YPGal)
- 30% ", 70% " (9 " 21 ")
- 20% ", 80% " (6 " 24 ")
- 10% ", 90% " (3 " 27 ")

I inoculated 10 mL of the above media ~~w/ either~~ w/ 10^6 cells that were grown overnight in either YPD or YPGal.

→ 11:15 am

After I inoculated the cultures I examined the O/N cultures and found that all cells in YPD were dark and most of the cells in YPGal were expressing GFP & mCherry. Interestingly about ~~[illegible]~~ 25% of the cells in YPGal were not expressing either GFP or mCherry.

Coulter Counts (2:15)

				I reran these after I finished w/ the rest	
Bkg	82000	52000			
50 Dex	5.002E6	946000	1.64 E6	5.822E6	5.946E6
50 Gal	3.818E6	3.442 E6		3.712E6	
30 Dex	1.148E6	1.052 E6		1.12 E6	
30 Gal	8.828E6	8.4E6			
20 Dex	4.856 E6	4.736E6			
20 Gal	8.838E6	9.212 E6			
10 Dex	4.814E6	4.948E6			
10 Gal	8.726E6	9.16E6			
Post Bkg	254000	94000			

→ These data seem a bit strange to me — I expected (at this point) that all the "Dex" cultures would be the same and all the "Gal" cultures would be the same.

MICROSCOPY — I looked under the microscope — all "Gal" were still v. bright and all "Dex" dark.

1/8/09

Coulter Counts (5:00pm)

Bkg	38000	20000
50 Dex	$1.948E^6$	$1.786E^6$
50 Gal	$1.296E^7$	$1.289E^7$
30 Dex	$2.608E^6$	$2.562E^6$
30 Gal	$1.244E^7$	$1.262E^7$
20 Dex	$2.29E^6$	$2.2E^6$
20 Gal	$1.277E^7$	$1.334E^7$
10 Dex	$1.958E^6$	$1.604E^6$
10 Gal	$1.513E^7$	$1.49E^7$
Post Bkg	392000	260000

- Under the microscope, all of the cultures from the o/N YPD were dark and all from YPGal were still bright

1/9/09

This morning I examined the eight cultures from yesterday under the microscope — all eight were green.

I think that the purdurance of the ~~fast~~ fusion proteins is too high to reliably determine when the genes are turned on.

I am not sure that the mixed glucose/galactose is a good idea — instead I should keep them in the situation where only one sugar is present by alternating between YPD & YPGal.

1/9/09

PILOT EXPERIMENT 2: DETERMINING DILUTIONS

· Last night I inoculated 5 ml YPD and 5 ml YPGal w/ a single large colony of yGIL345 (GAL1-GFP, GAL10-mCherry) and yGIL356 (WT)

· Today I diluted the ~~glucose~~ YPD cultures 1:300 (30λ into 10 ml YPD) and the YPGal 1:300 (30λ into 10 ml YPGal) and 1:100 (100λ into 10 ml YPGal) ~ 11:30.

Coulter Counts (6:00)

	Bkg	52000	32000
yGIL 345	YPD 1:300	$1.119E^7$	$1.174E^7$
356	YPD 1:300	$1.181E^7$	$1.177E^7$
345	YPG 1:300	$2.209E^7$	$2.199E^7$
356	YPG 1:300	$1.797E^7$	$1.754E^7$
345	YPG 1:100	$3.033E^7$	$3.068E^7$
356	YPG 1:100	$3.931E^7$	$3.963E^7$
	Post Bkg	$1.21E^6$	718000

I examined the cultures of yGIL345 under the microscope – in the YPD cultures all cells were dark. I YPG almost all cells were bright – there were still a few dark cells but not the ~25% I saw before (p 40)

Using the ~~1:300~~ 1:670 dilutions, I set up 16 cultures:

TUBE	CURRENT MEDIA	STRAIN	DILUTED INTO
1,9	YPD	yGIL 345	YPD
2,10	↓	"	YPG
3,11	↓	356	YPD
4,12	↓	"	YPG
5,13	YPG	345	YPD
6,14	↓	"	YPG
7,15	↓	356	YPD
8,16	↓	"	YPG

· tubes 1-8: ~~1:300~~ 1:670 dilution (15λ into 10 ml)

· tubes 9-16: 1:100 dilution (100λ into 10 ml)

→ dilutions @ 6:45pm

1/10/09

Coulter Counts (6:45 am)

				Dilution	Strain	Previous Media	Current Media	Dilute into
Bkg	72000	66000						
Tube 1	2.122 E7	2.128 E7		15 λ	yGIL345	YPD	YP D	YPD
2	1.78 E7	1.837 E7		↓	"	↓	G	↓
3	2.764 E7	2.738 E7		↓	356	↓	D	↓
4	2.178 E7	2.192 E7		↓	"	↓	G	↓
5	2.006 E7	2.014 E7		↓	345	YPGal	D	YPG
6	2.194 E7	2.216 E7		↓	"	↓	G	↓
7	3.024 E7	3.036 E7		↓	356	↓	D	↓
8	2.107 E7	2.078 E7		↓	"	↓	G	↓
9	1.165 E8			100 λ	345	YPD	D	
10	4.427 E7			↓	"	↓	G	
11	1.231 E8			↓	356	↓	D	
12	4.34 E7			↓	"	↓	G	
13	1.066 E8			↓	345	YPGal	D	
14	6.043 E7			↓	"	↓	G	
15	1.364 E8			↓	356	↓	D	
16	8.127 E7			↓	"	↓	G	
Post Bkg	2.194 E6	1.632 E6						

- The 1:100 dilutions were too dense so I only counted each once.

- I decided to do a second transfer of tubes 1-8 back into their original media (YPD for 1-4 and YPG for 5-8)
  ↳ dilutions @ 7:30 – the delay is from coulter counting and setting up the next set of tubes

I examined the cells under the microscope. All cultures currently growing in YPD are dark regardless of prior Gal exposure. The YPG → YPG cultures are 100% bright. There were some issues w/ the YPD → YPG cultures: In the ~~1:300~~ 1:670 dilution only about 5% of cells were v. bright – some others appeared to have dim fluorescence. In the more saturated 1:100 dilution about half were bright.

↳ What is going on? It has long been known that even small amounts of glucose ~~at~~ will inhibit gal, but they must be using the galactose.

1/10/09

- In order to determine if the cells diluted from 2% Glucose could be growing on the, at most $\frac{15\lambda}{10{,}000\lambda} \cdot 2 = 0.003\%$ Glucose, I diluted both yGIL345 and yGIL356 from the YPD cultures (i.e. cultures 1 & 3 p. 43) into YP (15λ into 10 mL) — 9:00am rollerdrum
  → 8:15 pm: No growth in either YP tube

- Coulter Count (8:15 pm)

Bkg	68000	56000
1	3.955E7	4.035E7
2	2.803E7	2.735E7
3	4.677E7	4.637E7
4	3.221E7	3.226E7
5	2.438E7	2.463E7
6	2.302E7	2.304E7
7	2.804E7	2.759E7
8	3.184E7	3.238E7
PostBkg	1.248E6	634000

STRAIN	TRANSFERS
yGIL345	YPD → YPD → YPD
"	YPD → YPG → YPD
356	YPD → YPD → YPD
"	YPD → YPG → YPD
345	YPG → YPD → YPG
"	YPG → YPG → YPG
356	YPG → YPD → YPG
"	YPG → YPG → YPG

- MICROSCOPY (8:30 pm)

I kept the YPD → YPG cultures (tubes 2 & 10) in the rollerdrum. Now both tubes look saturated

TUBE	%Bright @ 6:45 am, p. 43	% Bright @ 8:30 pm
dd 2	~5%	~90%
dd 10	~50%	~90%

TUBE	TRANSFERS	% Bright @ 8:30pm
1	YPD → YPD → YPD	0 (All dark)
2	YPD → YPG → YPD	0 (All dark)
5	YPG → YPD → YPG	many faint, few bright
6	YPG → YPG → YPG	~100%

1/12/09

FITNESS ASSAYS ON GAL MUTANT STRAINS

I have decided to do the fitness assays under 3 conditions (YPD, YPG, and alternating between YPD & YPG)

Streak the following strains onto YPD from freezer:

yGIL 356 (WT)
368 (cis)
358 (cis)
359 (7-trans)
370 (7-trans)
361 (10-trans)
362 (10-trans)
363 (1-trans)
371 (1-trans)
373 (Δ)
375 GFP Ref.
376 dTomato Ref.

1/13/09

Today I made YPD and YPG media

15g Yeast Extract
30g Peptone
30g Dextrose (or Galactose)
1.5 L H_2O

→ I used the bulk dispenser to dispense ~10.6 ml in each tube and autoclaved the racks. I made 134 tubes each of YPD (blue caps) and YPG (green caps)

1/14/09

NOTES ON FITNESS ASSAYS

- I have decided to do 6 time points instead of the usual 4 — this way I will grow the mixed lines in YPG twice (∴ 2 YPD → YPG shifts & 2 YPG → YPD shifts)

	(1)	(2)	(3)	(4)	(5)	(6)
YPD :	D →	D →	D →	D →	D →	D
ALT :	D →	D →	G →	D →	G →	D
YPG :	G →	G →	G →	G →	G →	G

↑ (1): this is the 1:300 following original O/N; mix point

Schedule:

I am aiming for 9:30 dilutions

W(1/14)	Th(1/15)	F(1/16)	Sa(1/17)	Su(1/18)	M(1/19)
	Dilute 1:300 (2 pm)	Dilute Sample (2) (9:30 am)	Dilute Sample (4) (9:30 am)	Sample (6) (9:30am)	FACS
O/N cultures (5:30pm)	Mix; Dilute Sample (1) (9:30pm)	Dilute Sample (3) (9:30pm)	Dilute Sample (5) (9:30pm)		

- Inoculate 5 ml of YPD and YPG with single colony of the 12 strains on p. 45. → rollerdrum 5:30

1/15/09

NOTES

- This morning I looked at the cultures — all are fairly dense, except the Δ strain in YPG, as expected.

  ↳ However, I am a bit surprised by how dense this culture is — I will need to watch the culture after the 1:300 dilution — I expect this strain to drop out completely from the YPG fitness assay. There is a clear difference in the density between this and all other cultures → so they are undoubtedly using the galactose.

- I looked at the GFP & dTomato cultures under the scope — ~~[illegible]~~ I could clearly see fluorescence in all cultures, but it was a little more dim in the YPG cultures, possibly because the cells were at lower density.

  → ~~[illegible]~~ Duplicates for dTomato Ref.

- 2:00pm - dilute all O/N cultures 1:300 (30 λ into 10 mL)

- 3:30pm - I made new 1×PBS (w/ 0.1% Tween) for this experiment.

- I have decided to count each culture once at each dilution — I set aside 33 cuvettes

- Looking by eye at the O/N cultures on my bench, the GFP and dTomato are growing more slowly in both YPD & YPG — I will aim for 2:1 ref: exp

1/15/09

Coulter Counts (8:15 pm)

Bkg	68000	46000
WT-YPD	1.411E7	1.462E7
CIS.1-YPD	1.035E7	1.036E7
Ref.1-YPD	9.958E6	9.842E6
WT - YPG	7.498E6	7.738E6
CIS.1 - YPG	9.88E6	1.028E7
Ref.1 - YPG	7.256E6	7.214E6
Post Bkg	1.41E6	236000

- I should let the cells go through about one more doubling — I will start ~10.

- In ~~Bkg~~ YPD, the fluorescent strains have a disadvantage — it is not clear if that holds true in YPG. I may want the Ref:Exp more like 60:40

- The lack of growth of the Δ strain in YPG is very apparent in the 1:300 dilutions.

Coulter Counts (9:30 pm)

Bkg	18000	38000
WT-YPD	1.895E7	1.909E7
CIS.1-YPD	1.944E7	1.945E7
Ref.1-YPD	1.914E7	1.922E7
WT-YPG	1.234E7	1.24E7
CIS.1-YPG	1.259E7	1.273E7
Ref.1-YPG	1.152E7	1.13E7
Post Bkg	732000	260000

(WT-YPD through Ref.1-YPG:) 200 ~~300~~ λ Exp : 300 λ Ref

· Dilutions @ 10:00pm

↳ Since the density is a bit low, I did a 1:500 dilution (20 λ into 10 mL)

1/15/09

Coulter Count (11:00pm) ①

Bkg		28000	158000	22000
YPD	WT	1	2.734 E7	2.698 E7
	CIS.1	2	2.68 E7	2.648 E7
	CIS.2	3	2.707 E7	2.719 E7
	7.1	4	2.518 E7	2.455 E7
	7.2	5	2.569 E7	2.544 E7
	10.1	6	2.975 E7	3.048 E7
	10.2	7	2.665 E7	2.625 E7
	1.1	8	2.421 E7	2.462 E7
	1.2	9	2.572 E7	2.548 E7
	Δ	10	2.463 E7	2.454 E7
	GFP	11	2.508 E7	2.505 E7
YP6	WT	12	1.542 E7	1.542 E7
	CIS.1	13	1.49 E7	1.49 E7
	CIS.2	14	1.436 E7	1.455 E7
	7.1	15	1.413 E7	1.437 E7
	7.2	16	1.563 E7	1.539 E7
	10.1	17	1.55 E7	1.513 E7
	10.2	18	1.536 E7	1.508 E7
	1.1	19	1.527 E7	1.462 E7	1.528 E7
	1.2	20	1.443 E7	1.432 E7
	Δ	21	9.672 E6	9.684 E6
	GFP	22	1.647 E7	1.301 E7	1.269 E7
ALT (in YPD)	WT	23	2.75 E7	2.838 E7
	CIS.1	24	3.216 E7	3.221 E7
	CIS.2	25	2.922 E7	2.882 E7
	7.1	26	2.523 E7	2.54 E7
	7.2	27	2.516 E7	2.476 E7
	10.1	28	2.804 E7	2.866 E7
	10.2	29	2.368 E7	2.336 E7
	1.1	30	2.548 E7	2.531 E7
	1.2	31	2.717 E7	2.721 E7
	Δ	32	2.618 E7	2.602 E7
	GFP	33	2.221 E7	2.263 E7
Post Bkg			666000	558000

1/16/09

- Gal cultures look dilute

Test Count (9:40 am)

Bkg	254000	190000
WT-YPD	3.284E7	3.406E7
WT-GAL	4.656E6	4.308E6
Post Bkg	510000	214000

→ I will dilute 15λ from YPD cultures (into either YPD or YPG – for alternating culture) and 100λ from YPG.

- Dilutions 10:00 am

↳ sample 500λ from YPD & 1 ml from YPG

Coulter Count (11:00 am) (2)

	Bkg		62000	66000	
YPD	WT	1	3.775E7	3.785E7	
YPD	CIS.1	2	3.893E7	3.844E7	
YPD	CIS.2	3	4.041E7	4.011E7	
YPD	7.1	4	3.669E7	3.669E7	
YPD	7.2	5	3.83E7	3.755E7	
YPD	10.1	6	3.611E7	3.557E7	
YPD	10.2	7	3.744E7	3.755E7	
YPD	1.1	8	3.662E7	3.644E7	
YPD	1.2	9	3.736E7	3.753E7	
YPD	Δ	10	3.441E7	3.445E7	
YPD	GFP	11	3.66E7	3.612E7	
YPG	WT	12	5.448E6	4.64E6	4.766E6
YPG	CIS.1	13	4.23E6	4.374E6	
YPG	CIS.2	14	4.428E6	4.332E6	
YPG	7.1	15	4.398E6	4.348E6	
YPG	7.2	16	4.416E6	4.324E6	
YPG	10.1	17	4.376E6	5.192E6	4.614E6
YPG	10.2	18	4.576E6	4.588E6	
YPG	1.1	19	4.538E6	4.406E6	
YPG	1.2	20	4.432E6	4.424E6	
YPG	Δ	21	2.588E6	2.424E6	
YPG	GFP	22	3.792E6	3.908E6	
ALT (in YPD)	WT	23	~~2~~ 3.622E7	3.633E7	
ALT (in YPD)	CIS.1	24	3.679E7	3.655E7	
ALT (in YPD)	CIS.2	25	3.452E7	3.53E7	

CONT. ON p 51 →

1/16/09

Coulter Counts continued

ALT (in YPD)	7.1	26	3.564E7	3.554E7
	7.2	27	3.547E7	3.55E7
	10.1	28	3.624E7	3.634E7
	10.2	29	3.814E7	3.823E7
	1.1	30	3.287E7	3.413E7
	1.2	31	3.576E7	3.527E7
	Δ	32	3.427E7	3.455E7
	GFP	33	3.455E7	3.402E7
		Post Bkg	1.108E6	718000

NOTES

I have been following the general practices of fitness assays that I used previously (see "Evolution I" notebook):
- I am keeping all of the media in the 30° room (prewarmed)
- I have been keeping several rack in the 30° room as well

I has been taking ~1.5 h to complete diluting, sampling, and counting — the is about twice as long as I recall it taking before — this is because I am doing much more sonicating and counting. The dilutions themselves only take ~10 min.

I have been doing the dilutions, sampling and counting in this order:
- Dilutions (put back on rollerdrum as soon as possible)
- Sample 10λ for Coulter Counter
- Sample 0.5-1 ml for FACS
- Spin down; aspirate; resus. in 1 mL PBST; vortex; 4°
- sonicate
- count

1/17/09

Because of the way I started the expt, (p. 46) the YPD & ALT started from the same colony (initially inoculated into YPD for o/N) and the YPG culture from another colony (inoculated into YPG for o/N)

1/16/09

Test Counts (9:30 pm)

			Dilution
Bkg	462000	682000	
WT-YPD	3.115 E7	3.152 E7	→ 15 λ (into YPD); expect 3-3.5 E7 in 12 h
WT-YPG	4.258 E6	4.188 E6	→ 200 λ (into YPG); expect 0.8-1 E7 in 12 h
WT-ALT	3.178 E6	3.202 E6	→ 100 λ (into YPD); expect 3-3.5 E7 in 12 h
Post Bkg	1.21 E6	932000	

- Again I will sample 500 λ from YPD and 1 mL from YPG
- Dilutions — 10 pm

Coulter Count (11 pm) ③

	#				
Bkg		24000	42000		
YPD	1	3.656 E7	3.719 E7		WT
YPD	2	3.657 E7	3.587 E7		cis.1
YPD	3	3.856 E7	3.864 E7		cis.2
YPD	4	3.619 E7	3.594 E7		7.1
YPD	5	3.747 E7	3.788 E7		7.2
YPD	6	3.603 E7	3.658 E7		10.1
YPD	7	3.736 E7	3.701 E7		10.2
YPD	8	3.814 E7	3.815 E7		1.1
YPD	9	3.521 E7	3.543 E7		1.2
YPD	10	3.59 E7	3.553 E7		Δ
YPD	11	3.644 E7	3.599 E7		GFP
YPG	12	5.856 E6	5.732 E6		WT
YPG	13	5.104 E6	4.716 E6	4.458 E6	cis.1
YPG	14	4.85 E6	4.528 E6		cis.2
YPG	15	5.082 E6	4.958 E6		7.1
YPG	16	5.82 E6	5.406 E6		7.2
YPG	17	5.354 E6	5.206 E6		10.1
YPG	18	4.91 E6	4.84 E6		10.2
YPG	19	4.912 E6	4.776 E6		1.1
YPG	20	4.394 E6	4.616 E6		1.2
YPG	21	2.622 E6	2.466 E6		Δ
YPG	22	4.35 E6	4.43 E6		GFP

CONTINUED ON p. 53 →

1/16/09

Coulter Counts Continued

ALT (in YPG)	23	3.554 E6	3.296 E6	WT	
	24	3.122 E6	3.034 E6	CIS.1	
	25	2.954 E6	3.004 E6	CIS.2	
	26	2.89 E6	2.896 E6	7.1	
	27	3.054 E6	2.862 E6	7.2	
	28	2.994 E6	2.876 E6	10.1	
	29	2.97 E6	3.092 E6	10.2	
	30	3.116 E6	2.762 E6	2.768 E6	1.1
	31	3.016 E6	3.074 E6	1.2	
	32	2.528 E6	2.398 E6	Δ	
	33	2.8 E6	3.066 E6	2.952 E6	GFP
	Post Bkg	82000	104000		

1/17/09

Test Counts (9:30am)

Bkg	50000	56000	~~[illegible]~~
WT-YPD	2.721 E7	2.73~~[illegible]~~4 E7	→ 15 λ (into YPD); expect 3 E7 in 12h
WT-YPG	7.274 E6	7.29 E~~[illegible]~~6	→ 100 λ (into YPG); expect 1 E7 in 12h
WT-ALT	1.09 E7	1.093 E7	→ 100 λ (into YPG); expect 1 E7 in 12h
Post Bkg	374000	192000	

DILUTIONS (10am)

Coulter Counts (11am)

(4)

	Bkg	36000	44000	
YPD	1	3.495 E7	3.482 E7	WT
	2	3.318 E7	3.365 E7	CIS.1
	3	3.442 E7	3.459 E7	CIS.2
	4	3.347 E7	3.303 E7	7.1
	5	3.419 E7	3.305 E7	7.2
	6	3.251 E7	3.197 E7	10.1
	7	3.23 E7	3.175 E7	10.2
	8	3.105 E7	3.083 E7	1.1
	9	3.14 E7	3.14 E7	1.2
	10	2.868 E7	2.836 E7	Δ
	11	3.295 E7	3.287 E7	GFP

CONTINUED ON p. 54 →

1/17/09

Coulter Counts cont.

YPG	12	$8.552E^6$	$1.146E^7$	$9.154E^6$	WT
	13	$8.2E^6$	$8.016E^6$		CIS.1
	14	$6.866E^6$	$6.74E^6$		CIS.2
	15	$7.598E^6$	$7.646E^6$		7.1
	16	$8.564E^6$	$8.37E^6$		7.2
	17	$7.968E^6$	$7.802E^6$		10.1
	18	$7.764E^6$	$6.884E^6$	$7.06E^6$	10.2
	19	$7.028E^6$	$7.276E^6$		1.1
	20	$7.036E^6$	$7.04E^6$		1.2
	21	$3.76E^6$	$3.898E^6$		Δ
	22	$6.952E^6$	$6.28E^6$		GFP
ALT (in YPD)	23	$1.414E^7$	$1.38E^7$		WT
	24	$1.321E^7$	$1.315E^7$		CIS.1
	25	$1.168E^7$	$1.142E^7$		CIS.2
	26	$1.219E^7$	$1.226E^7$		7.1
	27	$1.283E^7$	$1.288E^7$		7.2
	28	$1.175E^7$	$1.188E^7$		10.1
	29	$1.233E^7$	$1.264E^7$		10.2
	30	$1.07E^7$	$1.061E^7$		1.1
	31	$1.228E^7$	$1.187E^7$		1.2
	32	$9.574E^6$	$9.938E^6$		Δ
	33	$1.315E^7$	$1.278E^7$		GFP
	Post Bkg	290000	282000		

1/17/09

→ As usual @ 10pm

Test Counts (9:30pm)

			DILUTIONS	Sample
Bkg	342000	166000		
WT-YPD	$2.323E^{7}$	$2.298E^{7}$	15λ (into YPD)	0.5 mL
WT-YPG	$5.742E^{6}$	$5.26E^{6}$	~~100~~ 150λ (into YPG)	1 mL
WT-ALT	$9.738E^{6}$	$1.007E^{7}$	15λ (into YPD)	0.5 mL
Post Bkg	$1.258E^{6}$	256000		

Coulter Counts (11pm) ⑤

	Bkg	286000	230000		
YPD	1	$2.83E^{7}$	$2.861E^{7}$	WT	
	2	$2.882E^{7}$	$2.875E^{7}$	CIS.1	
	3	$2.833E^{7}$	$2.884E^{7}$	CIS.2	
	4	$2.817E^{7}$	$2.773E^{7}$	7.1	
	5	$2.907E^{7}$	$2.906E^{7}$	7.2	
	6	$2.746E^{7}$	$2.707E^{7}$	10.1	
	7	$2.794E^{7}$	$2.805E^{7}$	10.2	
	8	$2.615E^{7}$	$2.626E^{7}$	1.1	
	9	$2.633E^{7}$	$2.635E^{7}$	1.2	
	10	$2.549E^{7}$	$2.507E^{7}$	Δ	
	11	$2.724E^{7}$	$2.737E^{7}$	GFP	
YPG	12	$6.738E^{6}$	$6.472E^{6}$	$6.318E^{6}$	WT
	13	$6.84E^{6}$	$6.414E^{6}$	$6.294E^{6}$	CIS.1
	14	$4.966E^{6}$	$4.99E^{6}$	CIS.2	
	15	$6.142E^{6}$	$5.782E^{6}$	7.1	
	16	$6.502E^{6}$	$6.284E^{6}$	7.2	
	17	$5.394E^{6}$	$5.502E^{6}$	10.1	
	18	$4.924E^{6}$	$5.456E^{6}$	$5.094E^{6}$	10.2
	19	$5.25E^{6}$	$5.22E^{6}$	1.1	
	20	$5.09E^{6}$	$5.134E^{6}$	1.2	
	21	$2.8E^{6}$	$2.848E^{6}$	Δ	
	22	$4.718E^{6}$	$4.532E^{6}$	GFP	
ALT (in YPG)	23	$1.189E^{7}$	$1.121E^{7}$	WT	
	24	$1.04E^{7}$	$1.026E^{7}$	CIS.1	
	25	$9.55E^{6}$	$9.29E^{6}$	CIS.2	
	26	$9.81E^{6}$	$9.64E^{6}$	7.1	
	27	$9.858E^{6}$	$1.046E^{7}$	7.2	
	28	$9.674E^{6}$	$1.007E^{7}$	10.1	

1/17/09

Coulter Counts CONT.

ALT (in YPG)	29	$1.066E^7$	$1.098E^7$	10.2
	30	$8.644E^6$	$8.588E^6$	1.1
	31	$9.888E^6$	$9.614E^6$	1.2
	32	$8.218E^6$	$8.122E^6$	Δ
	33	$1.045E^7$	$1.044E^7$	GFP
	Post Bkg	326000	268000	

1/18/09

· Sample 0.5 mL from YPD ~~& ALT~~ & 1 ml from YPG & ALT

Coulter Counts (10:45 am) ⑥

	Bkg	104000	218000		
~~YPG~~ YPD	1	$2.381E^7$	$2.456E^7$		WT
	2	$2.347E^7$	$2.336E^7$		CIS.1
	3	$2.356E^7$	$2.357E^7$		CIS.2
	4	$2.279E^7$	$2.278E^7$		7.1
	5	$2.165E^7$	$2.254E^7$		7.2
	6	$2.102E^7$	$2.094E^7$		10.1
	7	$2.23E^7$	$2.212E^7$		10.2
	8	$2.102E^7$	$2.139E^7$		1.1
	9	$2.12E^7$	$2.094E^7$		1.2
	10	$1.902E^7$	$1.945E^7$		Δ
	11	$2.146E^7$	$2.142E^7$		GFP
YPG	12	$6.982E^6$	$6.814E^6$		WT
	13	$6.3E^6$	$6.01E^6$	$5.916E^6$	~~WT~~ CIS.1
	14	$5.282E^6$	$4.986E^6$		~~[illegible]~~ CIS.2
	15	$5.572E^6$	$5.25E^6$		~~[illegible]~~ 7.1
	16	$6.996E^6$	$7.128E^6$		~~[illegible]~~ 7.2
	17	$5.432E^6$	$5.54E^6$		~~[illegible]~~ 10.1
	18	$5.496E^6$	$5.37E^6$		~~[illegible]~~ 10.2
	19	$5.148E^6$	$5.146E^6$		~~[illegible]~~ 1.1
	20	$5.514E^6$	$5.23E^6$		~~[illegible]~~ 1.2
	21	$3.3E^6$	$3.574E^6$		~~[illegible]~~ Δ
	22	$5.274E^6$	$5.362E^6$		GFP

1/18/09

Coulter Counts Continued

ALT (in YPD)

23	6.892 E^6	6.98 E^6	WT	
24	5.98 E^6	5.654 E^6	CIS.1	
25	5.176 E^6	4.91 E^6	CIS.2	
26	5.196 E^6	4.876 E^6	4.93 E^6	7.1
27	5.04 E^6	5.006 E^6	7.2	
28	5.03 E^6	4.886 E^6	10.1	
29	5.656 E^6	5.714 E^6	10.2	
30	4.91 E^6	4.82 E^6	1.1	
31	5.352 E^6	5.308 E^6	1.2	
32	4.466 E^6	4.402 E^6	Δ	
33	6.022 E^6	5.902 E^6	GFP	
Post Bkg	264000	196000		

1/20/09 NOTES

Yesterday I transferred all of the samples to polystyrene 5mL FACS tubes and sonicated — 242 tubes, prep ~ 3.5h.

→ Unfortunately the LSRII was not keeping pressure properly. There were times when I could distinguish the dark cells & the dTomato cells, but then the two distributions would merge back into one. The GFP however looked great: glaringly bright in the positive control and little autofluorescence in the WT. We are going to try running the samples again tomorrow on the LSRII and possibly on the sorter.

It seems like I will need to redo the expt. using GFP as a ~~cor~~ Ref. I am toying w/ the idea of switching to minimal media → perhaps tonight I should start a pilot expt. to figure out what dilutions I will need to do if using minimal media.

→

1/20/08

NOTES

Pros & Cons of YP vs. Minimal media:

- I have been thus far doing all fitness assays in YP and I have a reasonable expectation for dilutions, timing, and cell densities

- YP is well buffered and is less likely to be conditioned
  ↳ I could always buffer minimal media

- Using minimal strongly constrains the carbon source, whereas gal mutants can grow (v. slightly) in YP.

1/26/09

ANALYSIS OF COULTER COUNTS

GENERATIONS PER 12h. INTERVAL

Medium	Strain	1	2	3	4	5
YPD	WT	6.55	6.48	6.45	6.30	6.34
YPD	cis.1	6.59	6.44	6.42	6.35	6.30
YPD	cis.2	6.61	6.46	6.39	6.31	6.31
YPD	7.1	6.60	6.49	6.42	6.33	6.30
YPD	7.2	6.61	6.50	6.39	6.36	6.23
YPD	10.1	6.39	6.52	6.38	6.33	5.97
YPD	10.2	6.56	6.49	6.35	6.37	6.27
YPD	1.1	6.62	6.55	6.29	6.34	6.29
YPD	1.2	6.59	6.44	6.38	6.33	6.28
YPD	D	6.55	6.54	6.28	6.38	6.23
YPD	GFP	6.59	6.50	6.41	6.32	6.26

	Ave Gen.	Ideal Dil.	Dil. Volume
YPD	6.41	605.10	16.53

Medium	Strain	1	2	3	4	5
YPG	WT	5.04	4.80	4.59	4.03	4.25
YPG	cis.1	4.97	4.70	4.45	4.37	4.18
YPG	cis.2	5.02	4.67	4.28	4.29	4.23
YPG	7.1	5.03	4.74	4.33	4.36	4.10
YPG	7.2	4.95	4.86	4.32	4.32	4.30
YPG	10.1	5.01	4.74	4.31	4.24	4.21
YPG	10.2	5.01	4.67	4.28	4.28	4.25
YPG	1.1	4.99	4.69	4.30	4.29	4.18
YPG	1.2	5.04	4.62	4.36	4.29	4.25
YPG	D	4.86	4.62	4.32	4.30	4.40
YPG	GFP	5.00	4.74	4.32	4.25	4.34

	Ave Gen.	Ideal Dil.	Dil. Volume
YPG	4.52	91.44	109.36

Medium	Strain	YPD	YPG	YPD	YPG	YPD
ALT	WT	6.48	4.14	6.01	4.41	5.99
ALT	cis.1	6.35	4.02	6.06	4.39	5.90
ALT	cis.2	6.40	4.04	5.96	4.40	5.88
ALT	7.1	6.56	3.99	6.05	4.38	5.84
ALT	7.2	6.57	4.02	6.07	4.37	5.80
ALT	10.1	6.46	3.99	6.00	4.43	5.81
ALT	10.2	6.70	3.97	6.02	4.48	5.84
ALT	1.1	6.49	4.01	5.95	4.39	5.93
ALT	1.2	6.48	4.05	5.98	4.39	5.90
ALT	D	6.49	3.87	5.98	4.43	5.89
ALT	GFP	6.64	4.05	6.08	4.39	5.94

	Ave Gen.	Ideal Dil.	Dil. Volume
YPD	6.14	462.42	21.63
YPG	4.21	67.31	148.56

CELL # VS. TIME

→ this is for each 10 ml culture tube – cells/mL is order of mag. less.

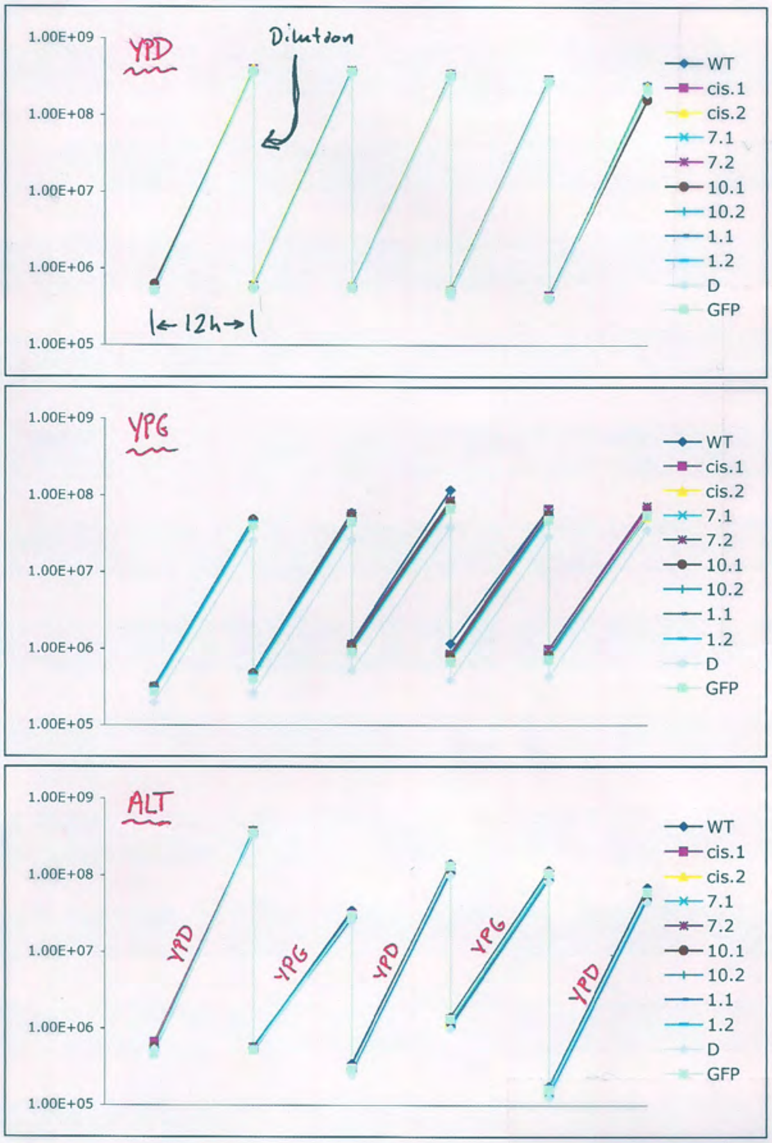

NOTES ON PROPAGATIONS

- I did a good job keeping the cultures in a reasonable range: no bottleneck was less than 10^5 and no culture reached 10^8 cells/mL.
- I was at the ideal dilution for YPD (each 12h period was more or less the same)
- I was hitting around the ideal dilution for YPG (110λ) – I did 20λ, 100λ, 200λ, 100λ, & 150λ
- I was off a bit w/ the ALT cultures — the dilutions and average culture densities were quite different

1/26/09 – FACS ANALYSIS

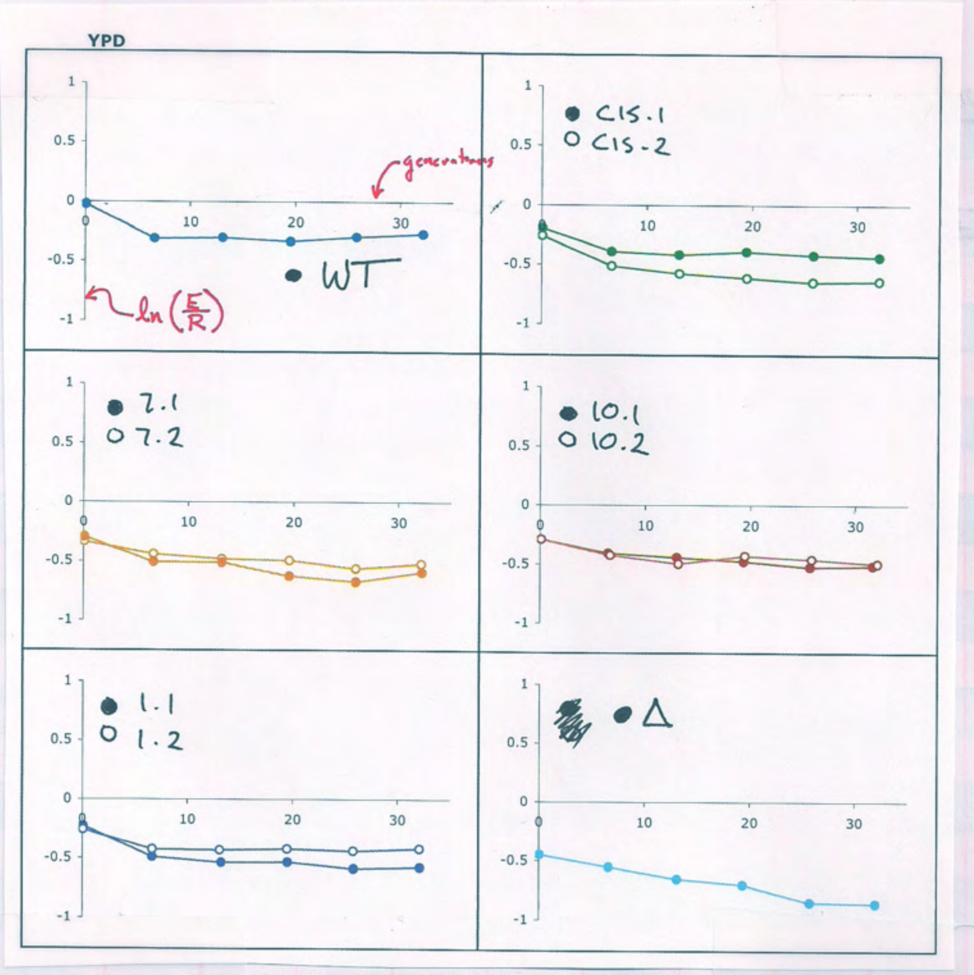

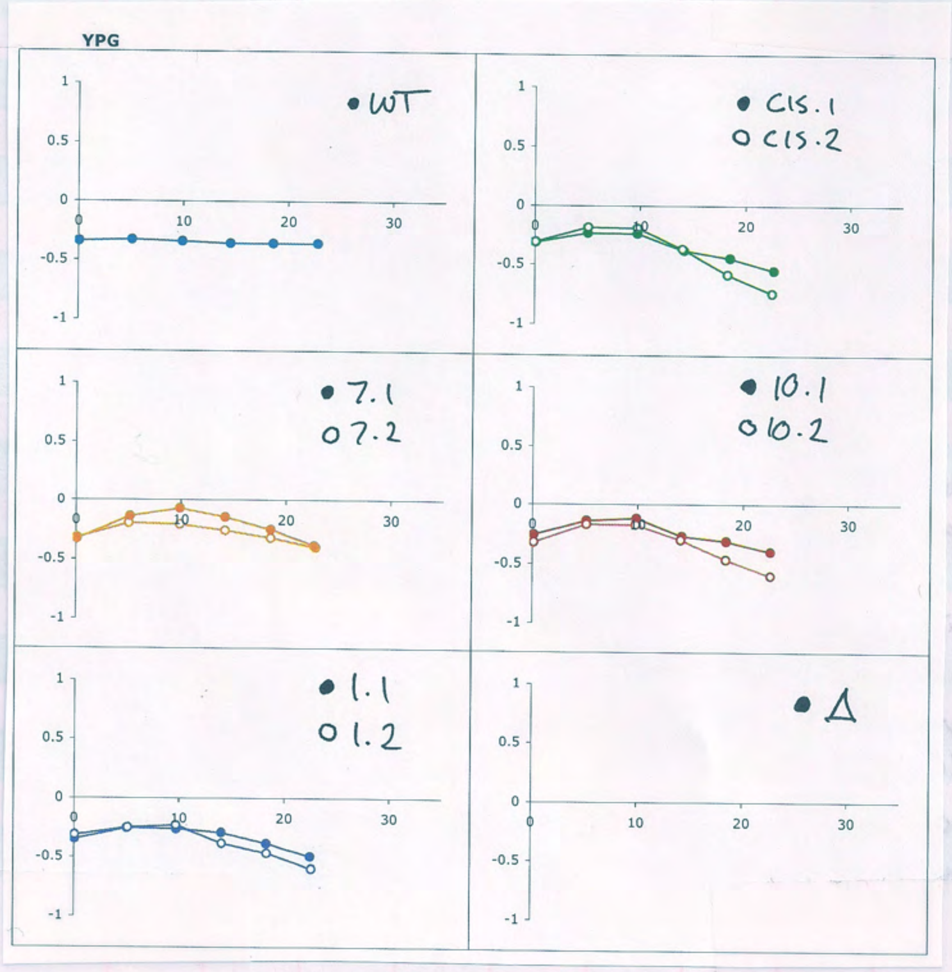

1/26/09 - FACS ANALYSIS CONT.

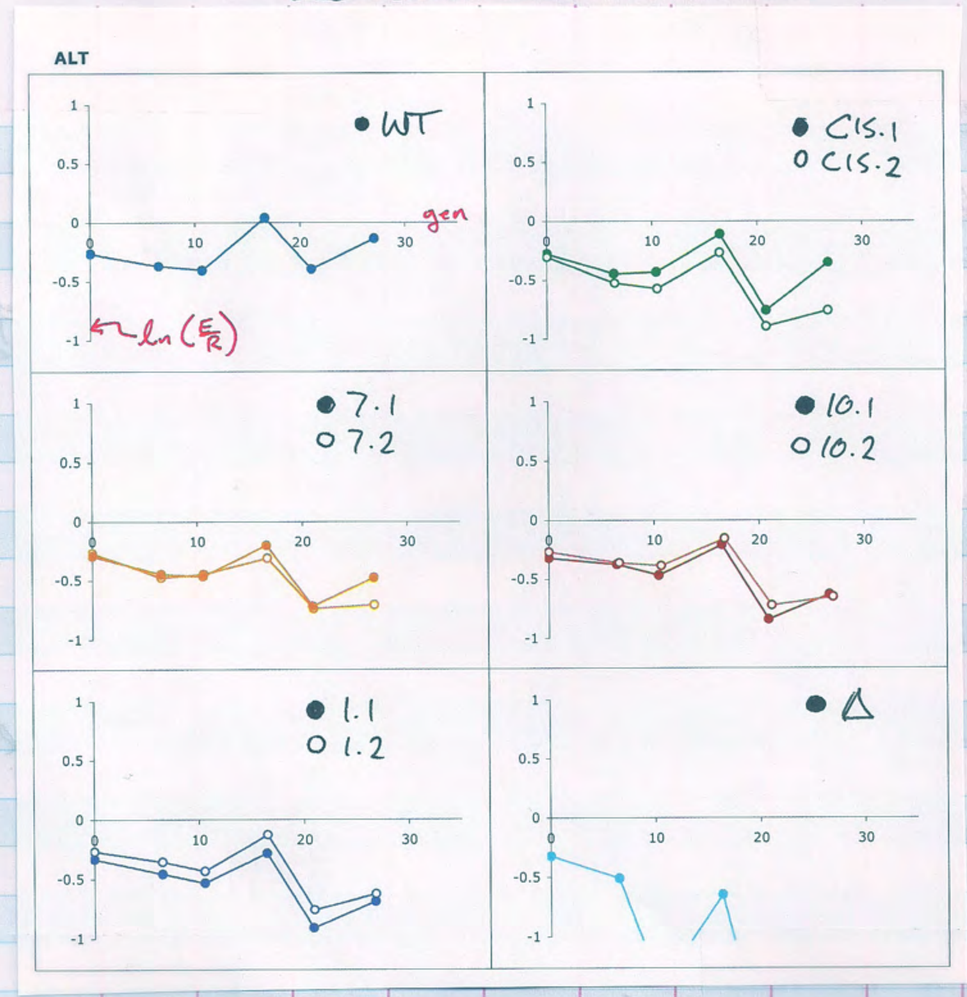

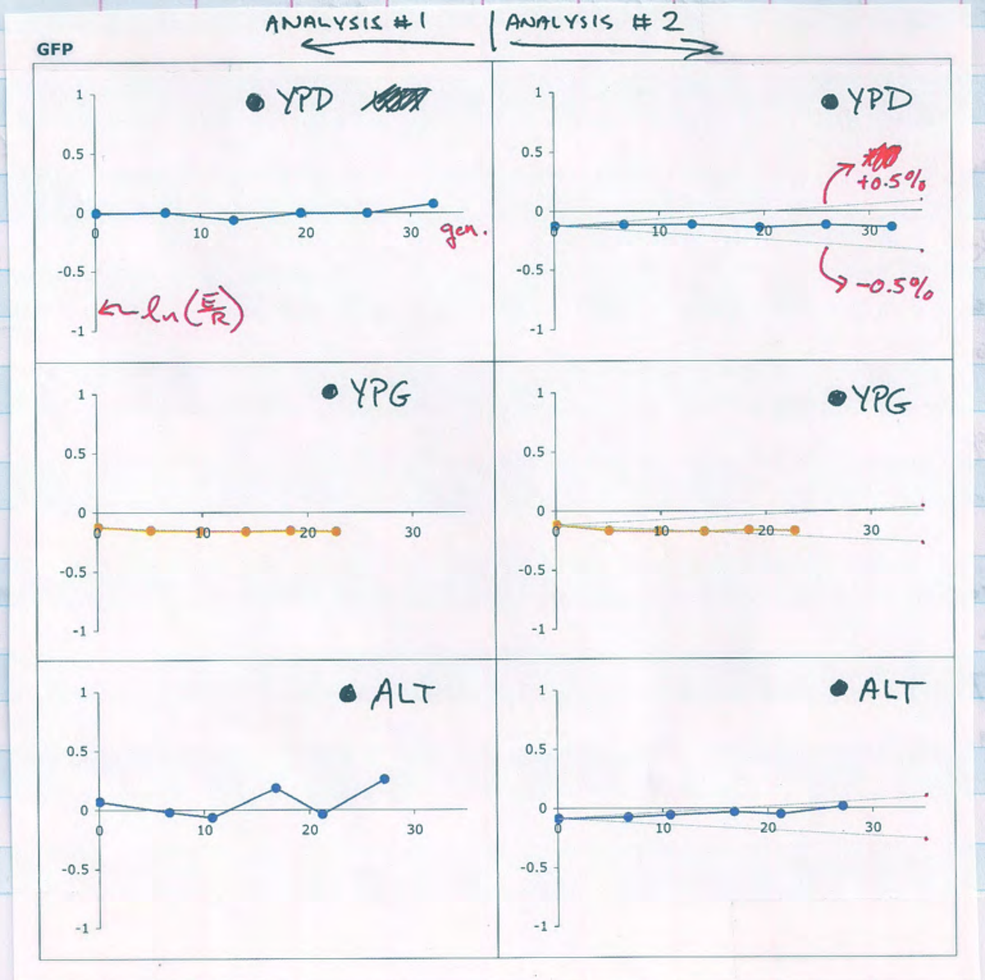

ANALYSIS #1 (left panels)

Here I distinguished the populations using only dTomato - from plot of FSC & dTomato

ANALYSIS #2 (right panels)

Here I used both fluorophores to distinguish the populations - from a plot of GFP & dTomato

1/26/09

NOTES AND ANALYSIS OF FITNESS ASSAYS

I was not able to run the samples last monday (1/19) as I had hoped due to a problem w/ the LSRII not keeping pressure. I stored my sonicated samples in the fridge until Wednesday and ran all of the samples at that time after the issue was fixed.

- The YPD cultures look fine (save for a "first 12h issue" like what I have seen before (see "Evolution I" notebook).

- I am terribly confused by the YPG cultures. The WT and GFP are both fine, but the heterozygous strains all show a strange trajectory. If I saw the opposite concavity I could explain it by adaptation. Other possible (but not equally plausable explanations) include:
  - Drug marker artefacts
  - Freq. dependent selection
  - Adaptation of the reference
  - Loss of reporter in YPG (but why not in WT & GFP)

The Alternating cultures are also difficult to interpret. In fact even though I think I know what is going on I don't think I can interpret these data. I see a zig-zag appearance to the ALT cultures. Some of this may be due to the presence of the fluorophore in the Ref strain since the zig-zag pattern is not as prominent in the GFP v. dTomato competition. The primary reason for this pattern ~~to the~~ arises from differences in the FACS data for YPD & YPG cultures — this results in differences in the overlap b/tw the dark & dTomato distributions when viewed in FSC v. dTomato. I was able to correct for this in the GFP v. dTomato competition by distinguishing the ~~[illegible]~~ populations using both fluorophores.

1/27/09

FITNESS IN YPD AND YPG

Analysis of fitness in YPD and YPG

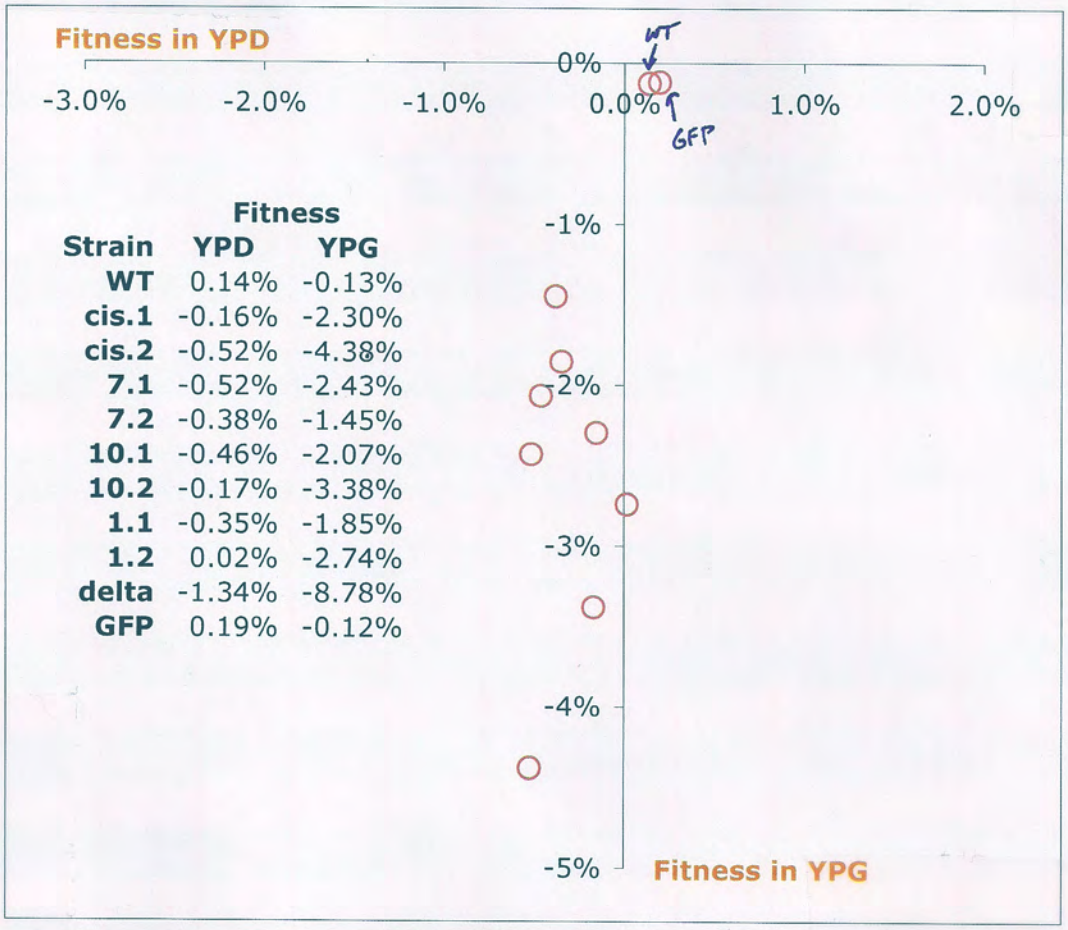

	Fitness	
**Strain**	**YPD**	**YPG**
**WT**	0.14%	-0.13%
**cis.1**	-0.16%	-2.30%
**cis.2**	-0.52%	-4.38%
**7.1**	-0.52%	-2.43%
**7.2**	-0.38%	-1.45%
**10.1**	-0.46%	-2.07%
**10.2**	-0.17%	-3.38%
**1.1**	-0.35%	-1.85%
**1.2**	0.02%	-2.74%
**delta**	-1.34%	-8.78%
**GFP**	0.19%	-0.12%

Fitness in ~~Y~~ YPD

I estimated the slopes from top panels on p. 60. and 61 (for GFP, left panels)
- I used all 6 points for Δ and GFP.
- I used points 2-6 for all others

Fitness in YPG
- I used all 6 points for WT and GFP
- I used points 3-6 for the rest.

1/27/09

SPORULATION AND DISSECTION OF GAL MUTANT STRAINS

- On 1/16 or 1/17 I took the original overnight cultures of the 12 strains used in this experiment, spun down 1 ml of saturated culture washed once w/ H_2O and resuspended in 4 ml of 2% KAc.

- On Saturday 1/24 I dissected 3 tetrads from each of the 12 cultures — today I picked all colonies off the plates and moved them to 96 well plates (w/ 100λ H_2O) and pin transferred to YPD, YPGal, YPD+Nat, YPD+Hyg, YPD+Kan, YNB-Glucose, and YNB-Galactose.

- Viable spores (#)

STRAIN	TETRAD 1	TETRAD 2	TETRAD 3
WT	4	4	4
CIS-1	4	4	4
CIS-2	4	4	2
7-1	3	4	4
7-2	4	4	4
10-1	4	4	3
10-2	4	4	4
1-1	4	4	4
1-2	4	3	2
Δ	3*	3	4
GFP	4	4	4
dTomato	4	4	4

* 1 of 3 has fitness defect

I looked at the GFP & dTomato segregants under the microscope
→ for GFP, I see 2:2 segregation of strong & weak fluorescence

STRONGLY FLUORESCENT	WEAKLY FLUORESCENT
1A, 1D, 2B, 2C, 3B, 3C	1B, 1C, 2A, 2D, 3A, 3D

All dTomato strains look the same

1/29/09

PHENOTYPIC ANALYSIS OF GAL MUTANT SEGREGANTS

	Hyg	G418	Nat	Gal
**WT**				
1A				+
1B				+
1C				+
1D				+
2A				+
2B				+
2C				+
2D				+
3A				+
3B				+
3C				+
3D				+
**cis.1**				
1A				+
1B	+	+	+	
1C				+
1D	+	+	+	
2A				+
2B				+
2C	+	+	+	
2D	+	+	+	+
3A	+	+	+	
3B				+
3C	+	+	+	
3D				+
**cis.2**				
1A	inviable			
1B				+
1C	inviable			
1D	+	+	+	
2A				+
2B	+	+	+	
2C				+
2D	+	+	+	
3A	+	+	+	
3B	+	+	+	
3C				+
3D				+
**7.1**				
1A		+	+	
1B	+			0
1C	+			0
1D		+	+	
2A	+			0
2B		+	+	
2C		+	+	
2D	+			0
3A		+	+	
3B	inviable			
3C	+	+	+	
3D	+			0
**7.2**				
1A	+			0
1B		+	+	
1C		+	+	
1D	+			0
2A		+	+	
2B	+			0
2C		+	+	
2D	+			0
3A		+	+	
3B		+	+	
3C	+			0
3D	+			0
**10.1**				
1A	inviable			
1B		+		0
1C	+		+	
1D	+		+	
2A	+		+	
2B		+		0
2C	+		+	
2D		+		0
3A		+		0
3B	+		+	
3C	+		+	
3D		+		0

	Hyg	G418	Nat	Gal
**10.2**				
1A	+		+	
1B		+		0
1C	+		+	
1D		+		0
2A	+		+	
2B	+		+	
2C		+		0
2D		+		0
3A	+		+	
3B		+		0
3C	+		+	
3D		+		0
**1.1**				
1A	+	+		0
1B			+	
1C			+	
1D	+	+		0
2A	+	+		0
2B			+	
2C			+	
2D	+	+		0
3A			+	
3B	+	+		0
3C	+	+		0
3D			+	
**1.2**				
1A	inviable			
1B	+	+		0
1C	inviable			
1D	+	+		0
2A			+	
2B			+	
2C	+	+		0
2D	inviable			
3A	+	+		0
3B	+	+		0
3C			+	
3D			+	
**delete**				
1A	+	+	+	
1B	+	+	+	
1C	+	+	+	
1D	+	+	+	
2A	inviable			
2B	+	+	+	
2C	+	+	+	
2D	+	+	+	
3A	inviable			
3B	+	+	+	
3C	+	+	+	
3D	+	+	+	
**GFP**				
1A			+	+
1B			+	+
1C			+	+
1D			+	+
2A			+	+
2B			+	+
2C			+	+
2D			+	+
3A			+	+
3B			+	+
3C			+	+
3D			+	+
**dTomato**				
1A			+	+
1B			+	+
1C			+	+
1D			+	+
2A			+	+
2B			+	+
2C			+	+
2D			+	+
3A			+	+
3B			+	+
3C			+	+
3D			+	+

1/28/09

Backcrossing gal-mutant strains to fluorescent strains

· In future rounds of fitness assays, I want to do two-colors — this is preferrable for two reasons: the presence of a fluorophore in only one strain can cause artefacts and two colors allows better discrimination of the populations.

· On Monday (1/26) I streaked the following strains from freezer:

yGIL 201
202
207
213
214
220
306
308
310
312
316
319
322
323
324
325
326
327
328
366

} these are all of the haploids used to construct the diploid gal mutants and fluorescent strains used in the fitness assays.

· Yesterday I patched the 6 ~~spore~~ segregants of yGIL375 that showed good GFP fluorescence onto YPD

→ I want to make a new GFP reference from these strains ~~that will be later today~~

· I want to cross all gal-mutant strains to dTomato strains (yGIL213 — MATa and yGIL220 — MATα)

· I want to cross the ~~cis~~ triple delete strains to GFP (the patched ones from yesterday) so that I can use the "cis" diploids as an alternative reference strain.

I looked under the microscope at the bright patched GFP strains and yGIL207 and yGIL214. yGIL214 is quite dim — this is the reason for the 2:2 segregation of bright fluorescence.

1/28/09

CROSSES

			1/29/09 # of viable zygotes
single colony of yGIL220	x	single colony of yGIL306	1
—	x	— 310	3
—	x	— 312	3
—	x	— 322	3
—	x	— 324	3
—	x	— 326	3
—	x	— 328	1
— 213	x	— 308	0
—	x	— 316	2
—	x	— 319	3
—	x	— 323	2
—	x	— 325	3
—	x	— 327	2
—	x	— 366	2
— 328	x	patch of yGIL378 segregant 1A	
—	x	— 1D	
—	x	— 2B	
—	x	— 2C	
—	x	— 3B	
—	x	— 3C	
— 366	x	— 1A	
—	x	— 1D	
—	x	— 2B	
—	x	— 2C	
—	x	— 3B	
—	x	— 3C	
patch of yGIL378 segregant 1A	x	— 1D	
—	x	— 2B	
—	x	— 2C	
—	x	— 3B	3
—	x	— 3C	

30° incubator @ 11:15 am

~~3~~ 1/28/09

@ 2:00 I looked at the 6 yGIL328/GFP crosses in order to determine the mating types of the yGIL378 segregants:

1A - α
1D - a
2B - α
2C - α
3B - a
3C - α

→ 1D did not mate w/ either 1A or yGIL366; ∴ use 3B instead

— Streak 1A & ~~1D~~ 3B to singles so I can freeze them down

— I will pick zygotes from the following:
yGIL328 × 1A
yGIL366 × ~~1D~~ 3B
1A × ~~1D~~ 3B

1/30/09

Freeze down:

yGIL378 segregant 1A ⟶ yGIL389
" 3B ⟶ yGIL390
yGIL389 × yGIL390 ⟶ yGIL391

Sporulate strains
- spin 1 mL culture
- wash once w/ 1 mL H_2O
- resuspend in 1 mL 2% KAc
- Add to tube of 3 mL 2% KAc
- RT rollerdrum

2/9/09

Today I dissected 5 tetrads from each of the 15 strains that have been sporulating for 10 days.

2/18/09

Segregation of Viability

Strain	4:0	4(f):0	3:1	3:1(f)	2:2	1:3	0:4
378-1B x 366	5						
378-1A x 328	3		1				1
220 x 306	4		1				
220 x 310	5						
220 x 312	3		2				
220 x 322	2		2	1			
220 x 324	4		1				
220 x 326	5						
220 x 328	1				3	1	
213 x 316	5						
213 x 319	5						
213 x 323	5						
213 x 325	5						
213 x 327	4	1					
213 x 366	3		1		1		

(f) - small colony

→ From this backcross I suspect that there may be a translocation in yGIL328 (triple Δ) & yGIL322 (1Δ)

TOTAL SPORE VIABILITY OF CROSSES INVOLVING 328 (here & p. 64)

	4:0	3:1	2:2	1:3	0:4
yGIL328	7	3*	4	1	1
yGIL322	3	4*	1		

↳ *1 each segregated a fitness defect in one spore

2/18/09

SCHEMATIC OF STRAIN CONSTRUCTION

Here is a concise figure showing the derivation of strains used in the 1st round of fitness assays

	MAT a			Diploid	MAT alpha			
WT	Deletion of GAL#				Deletion of GAL#			WT
	7	10	1		1	10	7	
201 ------>	------	------	------ ---->	356 (WT)	<---- ------	------	------	------ 202
201 ------>	------	------	------ ---->	368 (cis 1)	<---- 366	<---- 319	<---- 308	<---- 202
201 ---->	306 ---->	312 ---->	328* ---->	358* (cis 2)	<---- ------	------	------	------ 202
201 ---->	306 ------	------	------ ---->	359 (7-1)	<---- 327	<---- 316	<---- ------	------ 202
201 ------	------ ---->	310 ---->	326 ---->	370 (7-2)	<---- ------	------	308	<---- 202
201 ------	------ ---->	310 ------	------ ---->	361 (10-1)	<---- 325	<---- ------	308	<---- 202
201 ---->	306 ------	------ ---->	324 ---->	362 (10-2)	<---- ------	316	<---- ------	------ 202
201 ---->	306 ---->	312 ---->	------ ---->	363 (1-1)	<---- 323	<---- ------	------	------ 202
201 ------	------	------ ---->	322* ---->	371* (1-2)	<---- ------	319	<---- 308	<---- 202
201 ---->	306 ---->	312 ---->	328* ---->	373* (delete)	<---- 366	<---- 319	<---- 308	<---- 202

* Possible translocation

2/18/09 SUMMARY SHEETS FROM BACKCROSSES

Strain		Nat	G418	YPG	
378-1B x 366	1A			+	
A	1B	+	+		
1Δ, 10Δ, 7Δ	1C	+	+		
	1D	+	+	+	
	2A	+		+	
	2B			+	
	2C	+	+		⇇ } one of 2 will be green
	2D	+	+		
	3A	+	+		
	3B			+	
	3C	+	+		
	3D	+		+	
	4A	+	+		
	4B	+	+		
	4C	+	+	+	
	4D	+		+	
	5A	+	+		⇇ } one of 2 will be green
	5B	+	+		
	5C			+	
	5D	+		+	
378-1A x ~~366~~ 328	1A				
B	1B	+	+		⇇
1Δ, 10Δ, 7Δ	1C	+	+		
	1D			+	
	2A	+	+		← } 1 of 2
	2B			+	
	2C	+		+	
	2D	+	+		←
	3A				
	3B	+	+		
	3C	+	+		
	3D	+	+		
220 x 306	1A	+			←
C	1B			+	
7Δ	1C			+	
	1D	+			←
	2A	+			
	2B	+			
	2C	+		+	
	2D			+	
	3A			+	
	3B				m
	3C	+		+	
	3D	+			←
	4A	+		+	
	4B				
	4C	+			
	4D	+			
220 x 310	1A		+		
D	1B			+	
10Δ	1C	+		+	
	1D	+	+		←
	2A		+		
	2B	+	+		←
	2C			+	
	2D	+		+	
	3A	+	+		←
	3B			+	
	3C	+		+	
	3D		+		
	4A	+	+		
	4B	+	+		
	4C	+	+	+	
	4D			+	
	5A	+		+	
	5B	+	+	+	
	5C		+		
	5D		+		

Strain		Nat	G418	YPG	
220 x 312	1A	+	+		←
E	1B			+	
7Δ, 10Δ	1C	+		+	
	1D		+		
	2A			+	
	2B		+		
	2C	+		+	
	2D	+	+		←
	3A	+	+		←
	3B		+		
	3C	+		+	
	3D			+	
220 x 322	1A	+			
F	1B	+		+	
1Δ	1C	+			
	1D	+		+	
	2A	+		+	
	2B	+		+	
	2C	+			
	2D	+			
220 x 324	1A	+			← } 1 of 2 red
G	1B			+	
7Δ, 1Δ	1C	+			←
	1D	+		+	
	2A			+	
	2B	+			← } 1 of 2 red
	2C	+			←
	2D	+		+	
	3A	+		+	
	3B			+	
	3C	+			← } 1 of 2 red
	3D	+			←
	4A			+	
	4B			+	
	4C	+			←
	4D	+			←
220 x 326	1A	+	+	+	← } 1 of 2 red
H	1B			+	
10Δ, 1Δ	1C	+		+	
	1D	+	+		←
	2A	+	+		←
	2B			+	
	2C			+	
	2D	+	+	+	←
	3A				
	3B	+	+		←
	3C	+	+		←
	3D			+	
	4A	+	+		
	4B	+	+		
	4C	+		+	
	4D	+		+	
	5A	+		+	
	5B	+		+	
	5C	+	+		
	5D	+	+		
220 x 328	1A			+	
I	1B	+		+	
1Δ, 10Δ, 7Δ	1C	+	+	+	[illegible]
	1D	+	+		

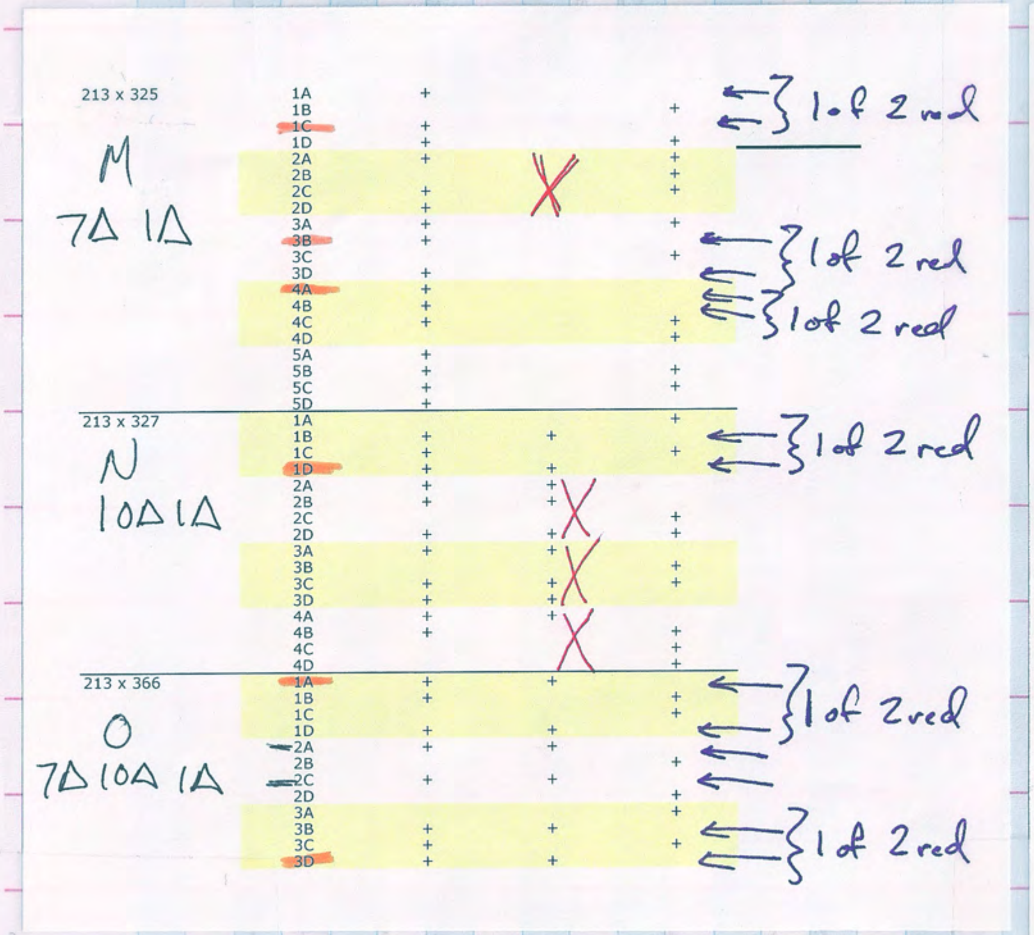
213 x 325
M
7Δ 1Δ
213 x 327
N
10Δ 1Δ
213 x 366
O
7Δ 10Δ 1Δ
1 of 2 red

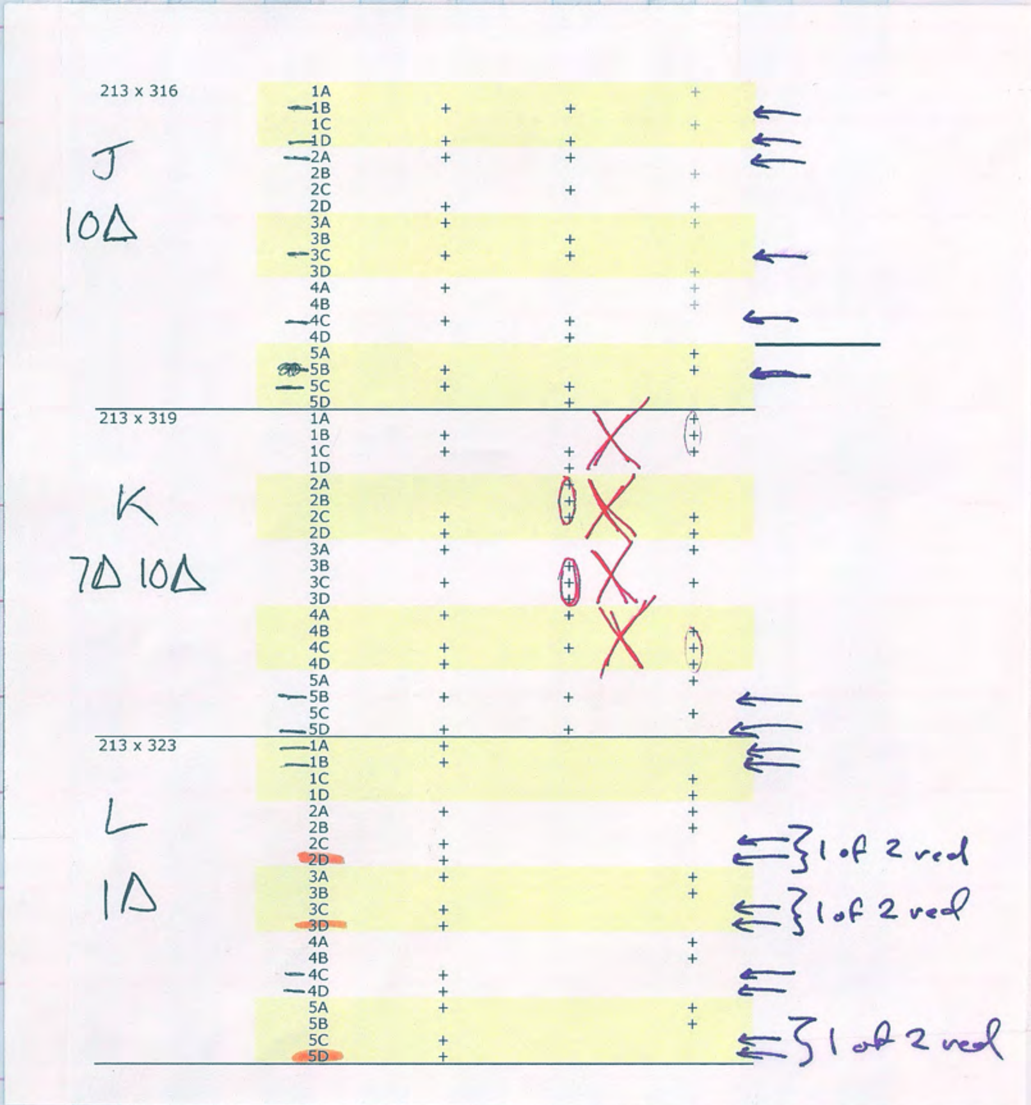
213 x 316
J
10Δ
213 x 319
K
7Δ 10Δ
213 x 323
L
1Δ
1 of 2 red

2/18/09

DISCUSSION OF BACKCROSSES

- I picked all 236 viable ~~spores~~ segregants from the 59 4-spore tetrads (p. 68). I ~~put~~ picked each segregant and put into a well of a 96-well plate containing 100 λ YPD.
- Segregants were pin transferred to YPD, YPD+G418, YPD+Nat, YPD+Hyg, & ~~YPD~~ YPGal. → Phenotypes are on pages 70 & 71.
  - The Hyg plates did not work (I added too little volume and did not give the drug long enough to diffuse)
  - Some of the pin transfers did not work well & some of the gal plates are hard to discern (grey type).
  - I put an X through 4-spore viables, which did not segregate 2:2 $G418^R$ or Nat^R (~~[illegible]~~ unless Gal1Δ::Nat is in this strain)

- For strains w/ gal1Δ::Nat and GFP/dTom :: Nat where I could not ~~not to~~ determine by segregation, I tested under the scope to find which gal⁻ segregant had the fluorescent protein.
  ↳ Highlighted in orange is the fluorescent segregant.

~~[illegible]~~

- From the 236 segregants I was left w/ 47 potentially correct strains. I mating type tested each of these strains by crossing to yGIL389 (MATa) & yGIL390 (MATα)

↳ 3 strains were ambiguous:
- H1A & H2D did not mate
- L5D mated well w/ 389, but I could see a few zygotes in the other cross as well

- I am left w/ 44 correct strains, of which I plan to freeze down 23

NOTE:: I suspect that strains yGIL393 ~~[illegible]~~ and yGIL394 contain a translocation.

2/18/09

GENOTYPES FOR THE ~~[illegible]~~ 44 CORRECT BACKCROSSED STRAINS

GAL genotype	Fluorphore	MAT	Spore	Strain #
1Δ 10Δ 7Δ	GFP	a	A2C	yGIL392
1Δ 10Δ 7Δ	GFP	a	A5B	
1Δ 10Δ 7Δ	GFP	a	B1C	yGIL393*
1Δ 10Δ 7Δ	GFP	a	B2D	
1Δ 10Δ 7Δ	GFP	alpha	B1B	yGIL394*
1Δ	dTomato	a	L1B	yGIL395
1Δ	dTomato	a	L2D	
1Δ	dTomato	a	L3D	
1Δ	dTomato	a	L4D	
1Δ	dTomato	alpha	L1A	yGIL396
1Δ	dTomato	alpha	L4C	
10Δ	dTomato	a	D2B	yGIL397
10Δ	dTomato	a	J4C	yGIL398
10Δ	dTomato	alpha	D1D	yGIL399
10Δ	dTomato	alpha	D3A	
10Δ	dTomato	alpha	J1B	yGIL400
10Δ	dTomato	alpha	J1D	
10Δ	dTomato	alpha	J2A	
10Δ	dTomato	alpha	J3C	
10Δ	dTomato	alpha	J5C	
7Δ	dTomato	a	C1D	yGIL401
7Δ	dTomato	a	C3D	
7Δ	dTomato	alpha	C1A	yGIL402
1Δ 10Δ	dTomato	a	H2A	
1Δ 10Δ	dTomato	a	H3B	yGIL403
1Δ 10Δ	dTomato	a	H3C	
1Δ 10Δ	dTomato	alpha	N1D	yGIL404
1Δ 7Δ	dTomato	a	G2C	yGIL405
1Δ 7Δ	dTomato	a	G3D	
1Δ 7Δ	dTomato	a	M1C	yGIL406
1Δ 7Δ	dTomato	a	M4A	
1Δ 7Δ	dTomato	alpha	G1C	yGIL407
1Δ 7Δ	dTomato	alpha	G4C	
1Δ 7Δ	dTomato	alpha	G4D	
1Δ 7Δ	dTomato	alpha	M3B	yGIL408
10Δ 7Δ	dTomato	a	E1A	yGIL409
10Δ 7Δ	dTomato	a	K5D	yGIL410
10Δ 7Δ	dTomato	alpha	E2D	yGIL411
10Δ 7Δ	dTomato	alpha	E3A	
10Δ 7Δ	dTomato	alpha	K5B	yGIL412
1Δ 10Δ 7Δ	dTomato	a	O2A	yGIL413
1Δ 10Δ 7Δ	dTomato	alpha	O1A	
1Δ 10Δ 7Δ	dTomato	alpha	O2C	yGIL414
1Δ 10Δ 7Δ	dTomato	alpha	O3D	

* may contain translocation

- set up the following crosses:

	Genotype	Color	Crosses MATa	MAT[illegible]
	WT	green	yGIL390	yGIL389
	cis-1	green	yGIL390	B1B
REF	cis-2	green	A2C	yGIL389
	WT	red	yGIL213	yGIL220
	cis-1	red	yGIL213	O2C
	cis-2	red	O2A	yGIL220
	7-1	red	C1D	N1D
	7-2	red	H3B	C1A
	10-1	red	D2B	G1C
	10-2	red	M1C	J1B
	1-1	red	L1B	K5B
	1-2	red	E1A	L1A
	Δ	red	O2A	O2C

pick 5 zygotes for each cross

2/23/09 → frozen down (as listed)

yGIL415 → yGIL427

2/20/09

- 3-5 zygotes grew for each strain. On Sunday I will start cultures for freezedown & fitness assays. For now, put plates in 4° fridge.

2/21/09 — START OF SECOND ROUND OF GAL-MUTANT FITNESS ASSAYS

- Yesterday I prepared media for the fitness assay as before (p. 45). From the 1.5L I made 140 ~~#~~ tubes of each YPD & YPG.
  ↳ I autoclaved the remaining media in 100mL bottles (30-50 mL).

2/22/09

- Inoculate 5mL of YPD & YPG w/ a single colony off of the pulled zygote plates from fridge (p.73)
  ↳ I took half of the colony and inoculated YPG, then took the other half to inoculate YPD. → 30° rollerdrum @ 5:15pm

- I also inoculated 3mL of YPD w/ a single colony from the restreaked haploids to be frozen down (yGIL392-414, p.73)

2/23/09

- 2:00pm: - Dilute o/N YPD cultures 1:333 (30λ into 10 mL)
  - Dilute o/N YPG cultures 1:222 (45λ into 10mL)
  ↳ I kept the original Δ YPG strain on the rollerdrum — I will probably use this one in the expt.

NOTE: In this run of the fitness assay, I am doing all of the dilutions (starting w/ the one above) ~~#~~ in the 30° room.

#

2/23/09

Test Counts (8:15 pm)

Bkg	436000	582000
WT-YPD	———	spilled
CIS·1-YPD	1.081 E7	1.07 E7
REF-YPD	1.22 E7	1.185 E7
WT-YPG	1.352 E7	1.384 E7
CIS·1-YPG	1.641 E7	2.015 E7
REF-YPG	1.283 E7	1.269 E7
Post Bkg	536000	476000

They are a little ahead of what I expected — presumably because I did the dilutions in the warm room w/ prewarmed media — compare to p. 48 — plan for ~9:30 dilutions

I will mix all strains 250λ Exp :: 250λ Ref.

Dilutions YPD: 17λ into YPD
ALT: 17λ into YPD
YPG: 109λ into YPG
} dilutions @ 9:15 pm

Coulter counts (10 pm)

	Bkg		274000	274000	118000	54000	56000
YPD	WT	1	1.716 E7	2.015 E7	1.685 E7		
YPD	CIS·1	2	1.853E7	1.869E7			
YPD	CIS·2	3	2.04 E7	2.065 E7			
YPD	7·1	4	1.831 E7	1.717 E7			
YPD	7·2	5	1.817 E7	1.859E7			
YPD	10·1	6	1.841 E7	1.882E7			
YPD	10·2	7	1.848E7	1.8 E7			
YPD	1·1	8	1.719 E7	1.711 E7			
YPD	1·2	9	1.861 E7	1.796 E7			
YPD	Δ	10	2.083 E7	2.032 E7			
ALT (YPD)	WT	11	1.984 E7	1.916 E7			
ALT (YPD)	CIS·1	12	1.846 E7	1.792 E7			
ALT (YPD)	CIS·2	13	2.147E7	2.123 E7			
ALT (YPD)	7·1	14	1.991 E7	1.926 E7			

(1)

CONTINUED ON p76 →

Coulter Counts Continued

ALT (YPD)	7.2	15	$1.903E^7$	$2.316E^7$	$1.84E^7$
	10.1	16	$1.819E^7$	$1.784E^7$	
	10.2	17	$1.984E^7$	$1.997E^7$	
	1.1	18	$1.743E^7$	$1.742E^7$	
	1.2	19	$1.791E^7$	$1.792E^7$	
	Δ	20	$1.897E^7$	$1.864E^7$	
YPG	WT	21	$1.68E^7$	$1.641E^7$	
	CIS.1	22	$1.6E^7$	$1.63E^7$	
	CIS.2	23	$1.805E^7$	$1.772E^7$	
	7.1	24	$1.702E^7$	$1.678E^7$	
	7.2	25	$1.678E^7$	$1.717E^7$	
	10.1	26	$1.691E^7$	$1.678E^7$	
	10.2	27	$1.675E^7$	$1.684E^7$	
	1.1	28	$1.629E^7$	$2.016E^7$	$1.647E^7$
	1.2	29	$1.568E^7$	$1.572E^7$	
	Δ	30	$1.8E^7$	$1.781E^7$	
Post Bkg			568000	350000	

~~[illegible]~~ For the YPG culture of Δ I used the original o/n (which I left on the rollerdrum) instead of the 1:222 dilution that was, expectedly, dilute.

2/24/09

Test Counts (8:45 am)

Bkg	56000	102000
WT-YPD	$1.739E^7$	$1.783E^7$
CIS1-YPD	$1.66E^7$	$1.617E^7$
WT-YPG	$1.927E^7$	$1.949E^7$
CIS.1-YPG	$2.095E^7$	$2.097E^7$
Post Bkg	630000	430000

Dilutions
- YPD: 15 λ into YPD
- ALT: 148 λ into YPG
- YPG: 100λ into YPG

} Dilutions 9am

2/24/09

COULTER COUNTS (10 am)

			74000	86000	
		Bkg	74000	86000	
YPD	WT	1	2.475E7	2.482E7	
YPD	CIS·1	2	2.709E7	2.367E7	2.38E7
YPD	CIS·2	3	2.369E7	2.28E7	
YPD	7·1	4	2.466E7	2.453E7	
YPD	7·2	5	2.483E7	2.466E7	
YPD	10·1	6	2.417E7	2.376E7	
YPD	10·2	7	2.44E7	2.409E7	
YPD	1·1	8	2.509E7	2.483E7	
YPD	1·2	9	2.511E7	2.539E7	
YPD	Δ	10	2.317E7	2.277E7	
ALT (YPD)	WT	11	2.544E7	2.501E7	
ALT (YPD)	CIS·1	12	2.317E7	2.337E7	
ALT (YPD)	CIS·2	13	2.393E7	2.368E7	
ALT (YPD)	7·1	14	2.392E7	2.35E7	
ALT (YPD)	7·2	15	2.454E7	2.447E7	
ALT (YPD)	10·1	16	2.447E7	2.508E7	
ALT (YPD)	10·2	17	2.434E7	2.378E7	
ALT (YPD)	1·1	18	2.31E7	2.324E7	
ALT (YPD)	1·2	19	2.424E7	2.403E7	
ALT (YPD)	Δ	20	2.441E7	2.394E7	
YPG	WT	21	2.347E7	2.309E7	
YPG	CIS·1	22	2.357E7	2.316E7	
YPG	CIS·2	23	2.424E7	2.438E7	
YPG	7·1	24	2.559E7	2.535E7	
YPG	7·2	25	2.37E7	2.351E7	
YPG	10·1	26	2.748E7	2.682E7	
YPG	10·2	27	2.414E7	2.405E7	
YPG	1·1	28	2.403E7	2.327E7	
YPG	1·2	29	2.282E7	2.283E7	
YPG	Δ	30	1.535E7	1.5E7	
		Post Bkg	564000	324000	

(2)

2/24/09

NOTES:

- While I was preparing the samples for FACS (after dilution), I broke the tube for 10.1-YPG. I pipetted 500λ off of the benchtop for FACS and 10λ for coulter counting — the counts for this sample look high, possibly from aspirating junk off of the bench. (p. 77).

- Cells seem to be growing a little faster this round — possibly because I am doing all of the dilutions in the 30° room. Other possibilities are less vol. in the media tubes or media quality — although it should be quite similar to last time.

- I am following the same order as last time (p. 51) except I am sampling for FACS before sampling for coulter.

- Sporulation — Today I sporulated the original o/n cultures which have sat o/n again on my bench.
  → spin ~1.5 mL → ~~wash~~ aspirate; wash w/ 1 mL H_2O resuspend in 1 mL 2% KAc; add to 3 mL tube of 2% KAc rollerdrum @ RT

- Since the cells were a little dense when I did the test counts, I moved the dilution time up to 9 — when I looked at the clock for the 1st dilution it was in-between 9 & 9:15 so I rounded up — But now I will stick to 9:00 dilutions.

2/24/09

Test Counts (8:30pm)

Bkg	144000	146000
WT-YPD	$1.735E^7$	$1.751E^7$
CIS·1-YPD	$1.613E^7$	$1.597E^7$
WT-ALT	$1.912E^7$	$1.902E^7$
CIS·1-ALT	$1.602E^7$	$1.604E^7$
WT-YPG	$2.032E^7$	$2.008E^7$
CIS·1-YPG	$2.12E^7$	2.184
Post Bkg	818000	568000

DILUTIONS @ 9pm
- YPD: 15 λ into YPD
- ALT: 20 λ into YPD
- YPG: 90 λ into YPG

Coulter Counts (9:45 pm)

	Bkg		32000	38000
YPD	WT	1	$2.409E^7$	$2.381E^7$
	CIS·1	2	$2.227E^7$	$2.156E^7$
	CIS·2	3	$2.192E^7$	$2.213E^7$
	7·1	4	$2.327E^7$	$2.345E^7$
	7·2	5	$2.332E^7$	$2.353E^7$
	10·1	6	$2.059E^7$	$2.039E^7$
	10·2	7	$2.303E^7$	$2.272E^7$
	1·1	8	$2.11E^7$	$2.104E^7$
	1·2	9	$2.242E^7$	$2.251E^7$
	Δ	10	$2.01E^7$	$1.932E^7$
ALT (YPG)	WT	11	$2.318E^7$	$2.273E^7$
	CIS·1	12	$1.856E^7$	$1.861E^7$
	CIS·2	13	$1.889E^7$	$1.837E^7$
	7·1	14	$1.797E^7$	$1.809E^7$
	7·2	15	$1.859E^7$	$1.839E^7$
	10·1	16	$1.928E^7$	$1.935E^7$
	10·2	17	$1.872E^7$	$1.89E^7$
	1·1	18	$1.762E^7$	$1.724E^7$
	1·2	19	$1.767E^7$	$1.736E^7$
	Δ	20	$1.391E^7$	$1.38E^7$

3

Coulter Counts continued

YPG	WT	21	2.251E7	2.212E7
	CIS·1	22	2.378E7	2.338E7
	CIS·2	23	2.435E7	2.435E7
	7·1	24	2.463E7	2.499E7
	7·2	25	2.297E7	2.361E7
	10·1	26	2.328E7	2.305E7
	10·2	27	2.45E7	2.392E7
	1·1	28	2.27E7	2.244E7
	1·2	29	2.319E7	2.31E7
	Δ	30	1.303E7	1.308E7
	Post Bkg		342000	312000

2/25/09

Test Counts (8:30 am)

Bkg	46000	44000
WT - YPD	1.69E7	1.668E7
CIS·1 - YPD	1.383E7	1.405E7
WT - ALT	1.244E7	1.212E7
CIS·1 - ALT	9.09E6	8.972E6
WT - YPG	1.577E7	1.562E7
CIS·1 - YPG	1.82E7	1.8E7
Post Bkg	672000	360000

Dilutions @ 9am
- YPD: 15 λ into YPD
- ALT: 150 λ into YPG
- YPG: 100 λ into YPG

Coulter Counts (~10am)

	Bkg		96000	92000
YPD	WT	1	2.292E7	2.315E7
	CIS·1	2	1.952E7	1.904E7
	CIS·2	3	1.911E7	1.905E7
	7·1	4	2.017E7	2.019E7
	7·2	5	2.113E7	2.129E7
	10·1	6	2.111E7	2.076E7
	10·2	7	2.128E7	2.111E7
	1·1	8	2.019E7	1.986E7
	1·2	9	1.931E7	1.888E7
	Δ	10	1.801E7	1.724E7

4

2/25/09

Coulter Counts Continued

ALT (YPD)	WT	11	1.766 E7	1.775 E7	
	CIS·1	12	1.249 E7	1.258 E7	
	CIS·2	13	1.297 E7	1.292 E7	
	7·1	14	1.197 E7	1.168 E7	
	7·2	15	1.264 E7	1.237 E7	
	10·1	16	1.285 E7	1.296 E7	
	10·2	17	1.321 E7	1.336 E7	
	1·1	18	1.262 E7	1.234 E7	
	1·2	19	1.358 E7	1.359 E7	
	Δ	20	8.712 E6	8.72 E6	
YPG	WT	21	1.92 E7	1.958 E7	
	CIS·1	22	2.118 E7	2.138 E7	
	CIS·2	23	1.946 E7	1.944 E7	
	7·1	24	2.151 E7	2.119 E7	
	7·2	25	1.98 E7	1.966 E7	
	10·1	26	1.849 E7	1.859 E7	
	10·2	27	2.019 E7	2.006 E7	
	1·1	28	1.9 E7	1.866 E7	1.85 E7
	1·2	29	1.877 E7	1.838 E7	
	Δ	30	8.912 E6	9.042 E6	
	Post Bkg		354000	298000	

Test Counts (8:30 pm)

Bkg	418000	1.018 E6
WT - YPD	1.319 E7	1.314 E7
Cis·1 - YPD	1.128 E7	1.052 E7
WT - ALT	1.713 E7	1.724 E7
Cis·1 - ALT	1.368 E7	1.345 E7
WT - YPG	1.27 E7	1.27 E7
Cis·1 - YPG	1.598 E7	1.602 E7
Post Bkg	688000	248000

DILUTIONS @ 9pm
- YPD: 20 λ into YPD
- ALT: 20 λ into YPD
- YPG: 100 λ into YPG

2/25/09

COULTER COUNTS (9:45 pm)

Group	Sample	#	Count 1	Count 2
	Bkg		40000	66000
YPD	WT	1	$1.691E^7$	$1.689E^7$
YPD	CIS·1	2	$1.426E^7$	$1.459E^7$
YPD	CIS·2	3	$1.507E^7$	$1.525E^7$
YPD	7·1	4	$1.6E^7$	$1.58E^7$
YPD	7·2	5	$1.548E^7$	$1.569E^7$
YPD	10·1	6	$1.492E^7$	$1.496E^7$
YPD	10·2	7	$1.514E^7$	$1.516E^7$
YPD	1·1	8	$1.556E^7$	$1.572E^7$
YPD	1·2	9	$1.589E^7$	$1.585E^7$
YPD	Δ	10	$1.351E^7$	$1.304E^7$
ALT (YPG)	WT	11	$1.998E^7$	$1.986E^7$
ALT (YPG)	CIS·1	12	$1.425E^7$	$1.39E^7$
ALT (YPG)	CIS·2	13	$1.367E^7$	$1.341E^7$
ALT (YPG)	7·1	14	$1.392E^7$	$1.381E^7$
ALT (YPG)	7·2	15	$1.386E^7$	$1.417E^7$
ALT (YPG)	10·1	16	$1.478E^7$	$1.448E^7$
ALT (YPG)	10·2	17	$1.46E^7$	$1.413E^7$
ALT (YPG)	1·1	18	$1.379E^7$	$1.33E^7$
ALT (YPG)	1·2	19	$1.366E^7$	$1.323E^7$
ALT (YPG)	Δ	20	$9.874E^6$	$9.554E^6$
YPG	WT	21	$1.471E^7$	$1.445E^7$
YPG	CIS·1	22	$1.884E^7$	$1.902E^7$
YPG	CIS·2	23	$1.452E^7$	$1.457E^7$
YPG	7·1	24	$1.877E^7$	$1.852E^7$
YPG	7·2	25	$1.645E^7$	$1.666E^7$
YPG	10·1	26	$1.318E^7$	$1.355E^7$
YPG	10·2	27	$1.756E^7$	$1.746E^7$
YPG	1·1	28	$1.472E^7$	$1.458E^7$
YPG	1·2	29	$1.504E^7$	$1.457E^7$
YPG	Δ	30	$6.504E^6$	$6.168E^7$
	Post Bkg		214000	178000

(5)

2/26/09

Coulter Counts (9:45 am)

		Bkg	30000	48000
YPD	WT	1	$1.744E^7$	$1.767E^7$
	CIS-1	2	$1.483E^7$	$1.437E^7$
	CIS-2	3	$1.48E^7$	$1.487E^7$
	7-1	4	$1.564E^7$	$1.57E^7$
	7-2	5	$9.148E^6$	$8.962E^6$
	10-1	6	$1.42E^7$	$1.469E^7$
	10-2	7	$1.444E^7$	$1.507E^7$
	1-1	8	$1.478E^7$	$1.464E^7$
	1-2	9	$1.4E^7$	$1.406E^7$
	Δ	10	$1.259E^7$	$1.24E^7$
ALT (YPD)	WT	11	$1.624E^7$	$1.631E^7$
	CIS-1	12	$9.116E^6$	$9.338E^6$
	CIS-2	13	$8.976E^6$	$8.844E^6$
	7-1	14	$8.534E^6$	$8.5E^6$
	7-2	15	$9.278E^6$	$9.596E^6$
	10-1	16	$9.796E^6$	$9.69E^6$
	10-2	17	$1.019E^7$	$9.914E^6$
	1-1	18	$8.982E^6$	$8.736E^6$
	1-2	19	$8.482E^6$	$8.852E^6$
	Δ	20	$5.494E^6$	$5.636E^6$
YPG	WT	21	$1.147E^7$	$1.124E^7$
	CIS-1	22	$1.559E^7$	$1.57E^7$
	CIS-2	23	$1.154E^7$	$1.127E^7$
	7-1	24	$1.594E^7$	$1.596E^7$
	7-2	25	$1.336E^7$	$1.284E^7$
	10-1	26	$9.522E^6$	$9.59E^6$
	10-2	27	$1.523E^7$	$1.471E^7$
	1-1	28	$1.178E^7$	$1.157E^7$
	1-2	29	$1.108E^7$	$1.121E^7$
	Δ	30	$3.732E^6$	$3.662E^6$
		Post Bkg	118000	92000

(6)

3/2/09

RESULTS

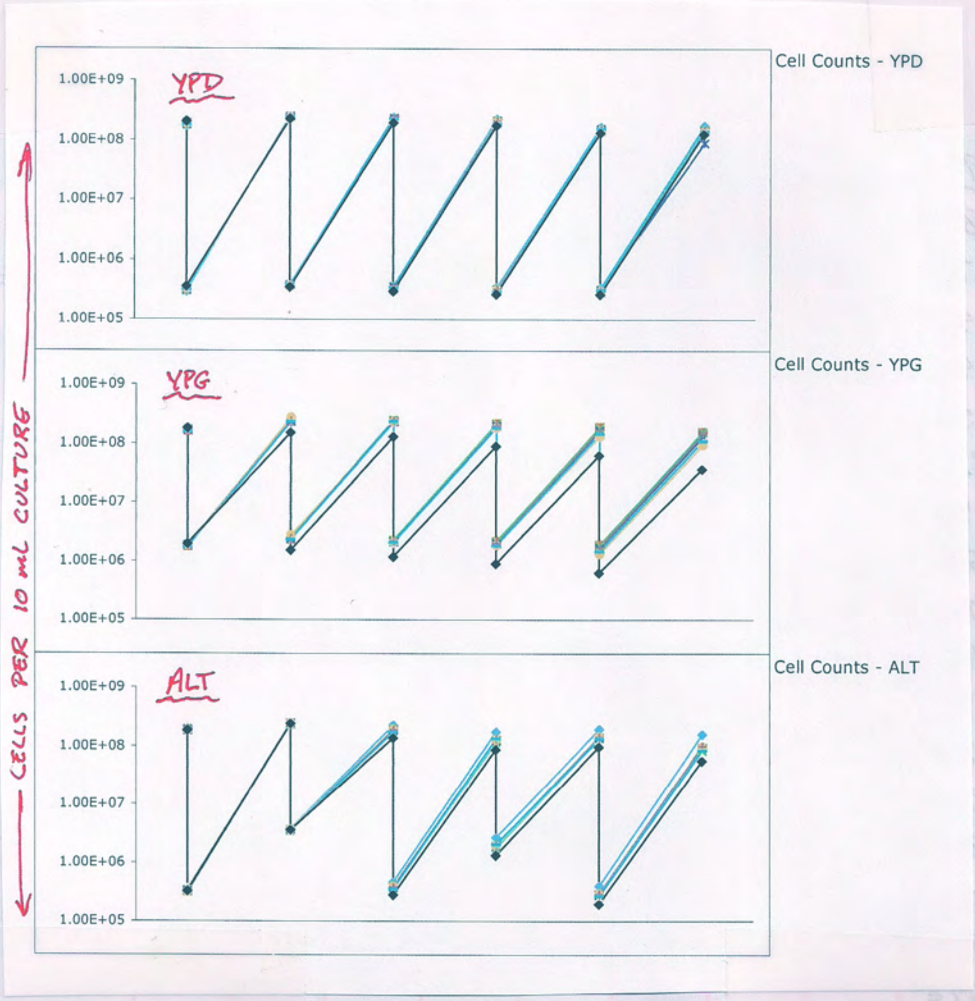

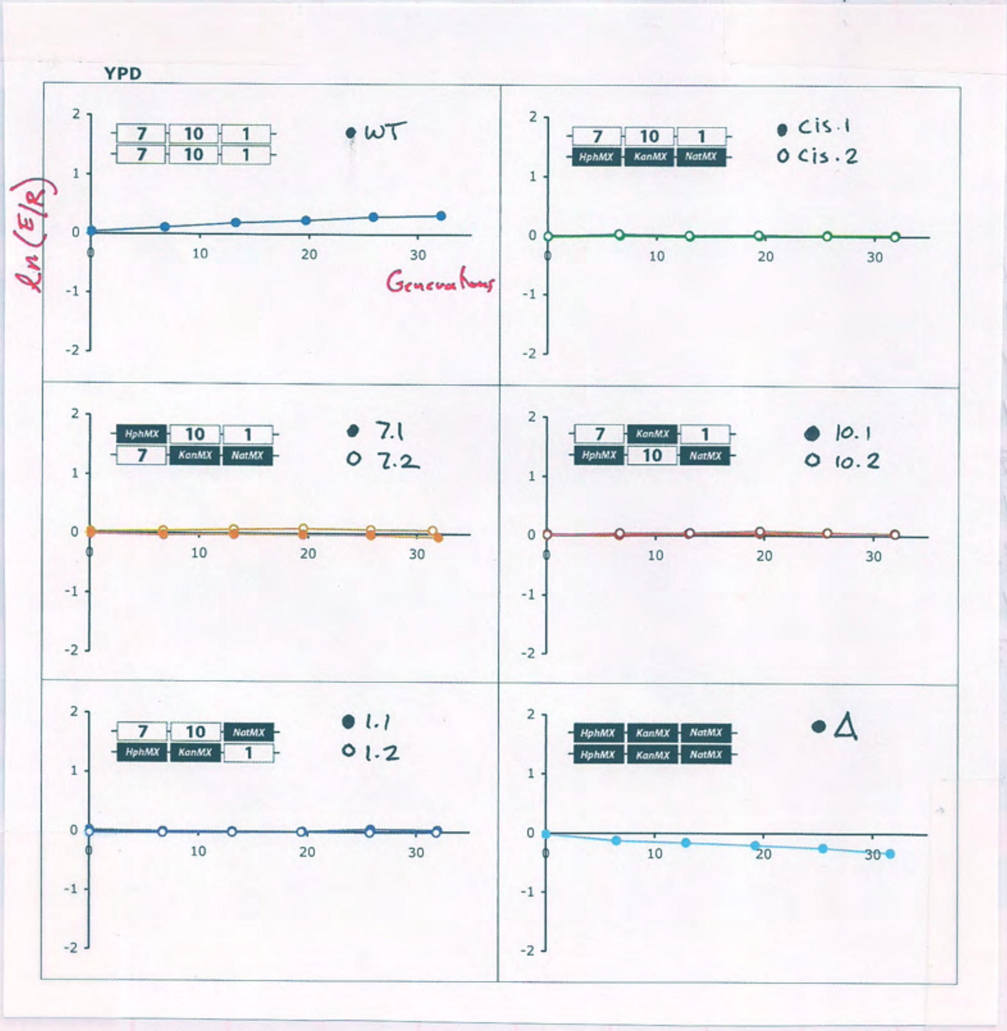

3/2/09

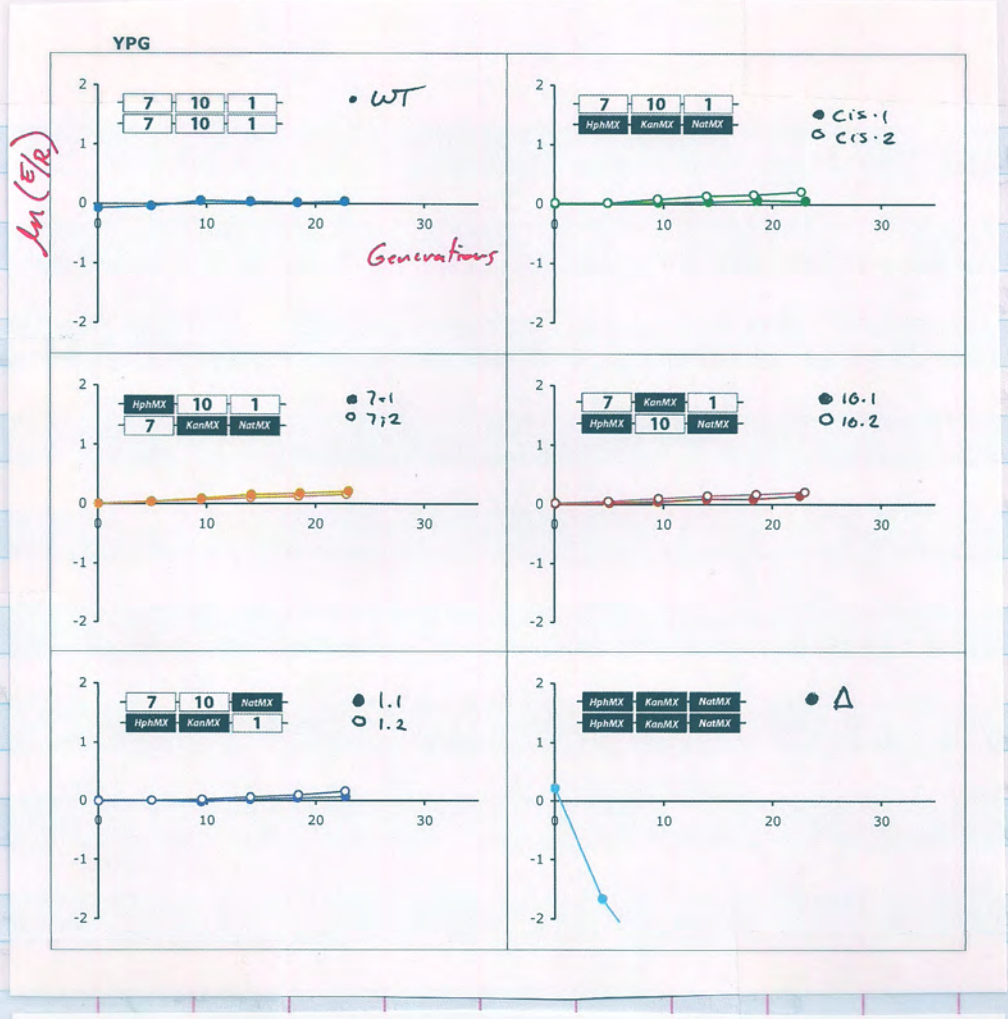
YPG
ln(E/R)
Generations
WT
cis.1
cis.2
7.1
7;2
10.1
10.2
1.1
1.2
Δ
HphMX
KanMX
NatMX

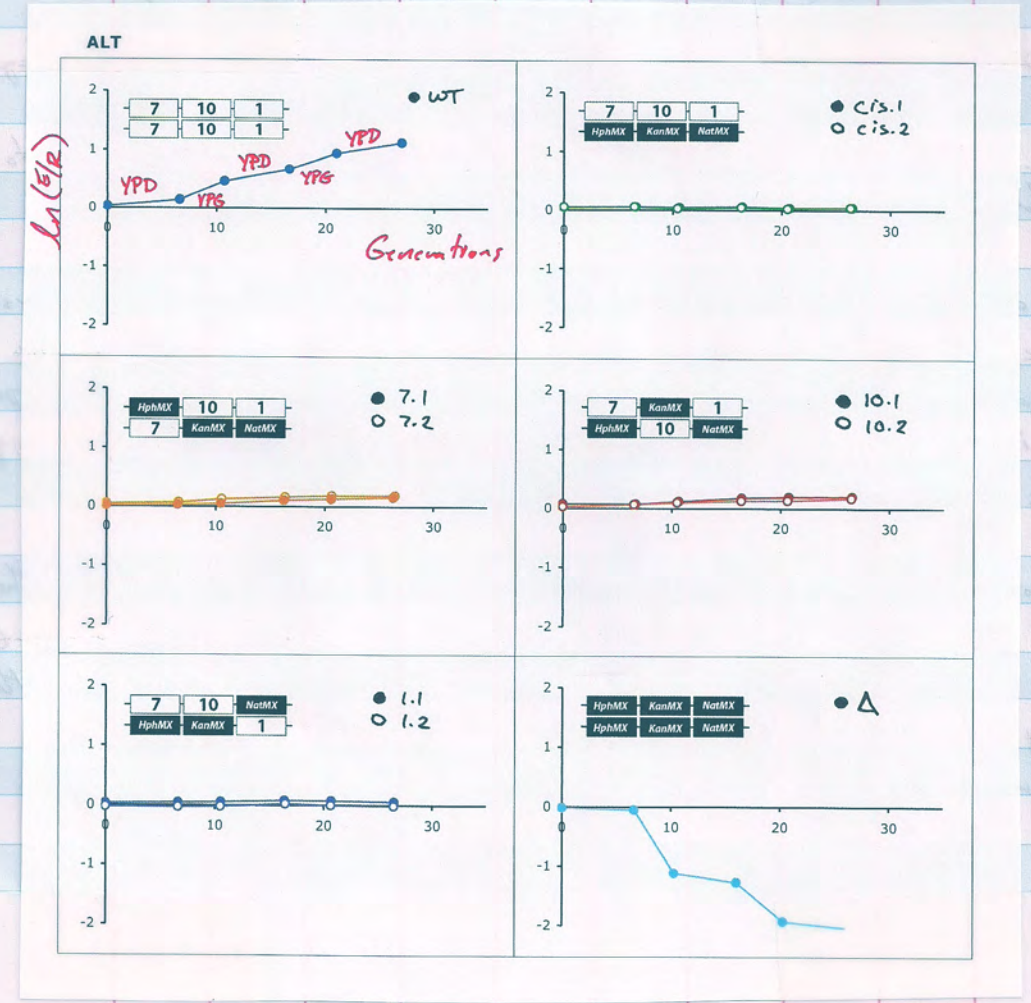
ALT
ln(E/R)
Generations
YPD
YPG
WT
cis.1
cis.2
7.1
7.2
10.1
10.2
1.1
1.2
Δ
HphMX
KanMX
NatMX

3/3/09

NOTES AND ANALYSIS OF FITNESS ASSAYS

OVERALL: This is probably the best-looking data I have generated - I consider this experiment a big success; however, I got the null result: there is no difference between cis, 1-trans, 10-trans, and/or 7-trans in any of the tested environments. Interestingly, the WT is equivalent in fitness in YPD (if the drug cassettes are controlled for), YPG, but has a ~4.5% advantage in changing conditions.

COULTER COUNTS: I did an excellent job w/ the dilutions, always diluting back when the cells were ~1-2 E^7 cells/mL - compare p. 84 w/ p. 59. I used the Coulter data to calculate generations for each dilution. I assume 10 mL cultures but to test this assumption I measured the vol (by weight) of 10 tubes of each YPD & YPG

- YPG - 100.31 g / 10 tubes ⟹ 10.0 mL/tube
- YPD - 100.09 g / 10 tubes ⟹ 10.0 mL/tube

} I am surprised this was right on.

YPD: In YPD I see no diff. for the 8 heterozygotes but an advantage for the WT and an equal-magnitude disadvantage for the het. Δ. This is consistent w/ a ~1% disadvantage due to the drug markers.

YPG: As expected, the Δ is terrible. No diff. in WT - this suggest (1) there is neg. feedback in GAL pathway and (2) the cost of the drug markers is mitigated because the GAL genes are highly expressed. There is a slight adv. (less than 0.5%) in the 8 heterozygotes - perhaps this reflects fitness diff. in reporter (?)

ALT: As expected, the Δ does poorly when shifted to YPG but is otherwise fine in YPD. There may be a slight advantage in 7-trans & 10-trans that is not seen in cis or 1-trans - I am not sure if that is significant or what to make of it. The WT has a v. strong advantage in the alternating conditions.

3/3/09

NOTES & ANALYSIS CONT.

FACS: To save time on FACS day I transferred the samples from microfuge tube to pre-chilled FACS tube the night before (2/26) and ran FACS last Friday (2/27). Unlike last time, there were no major issues w/ the LSRII (see pg. 57 & 62) The two-color expts were easily interpretable using one set of gates for YPD and another for YPG.

NEGATIVE FEEDBACK: That there is no difference in fitness between WT and the heterozygous reference suggests that there is sufficient negative feedback ~~in~~ to ensure that the proper amount of GAL1, 10, and 7. What is the nature of this feedback? Transcriptional? Translational? Post-translational? It may be useful to do arrays on these strains growing in Gal.

DYNAMICS OF SWITCHING: The simplest explanation for the advantage of WT in alternating YPD-YPG is that the second copy of GAL1-10-7 allows the cells to reach steady state faster. What about 3 copies? 4 copies? Also, is this advantage only in the Glucose → Galactose shift? Hard to tell from the plots because the actual shift probably occurs several generations (?) after transfer.

3/4/09

NEW STRAIN CONSTRUCTION: MOVING GAL1·10·7

- The previous experiment failed to show a difference between cis- and trans- configurations of the GAL genes. However this was not really a direct test of moving the GAL genes — in all cases the genes were still allelic (or nearly so).
- Instead, I want to actually move the GAL1-10-7 region to a different place in the genome and repeat the knockouts there. I have come up w/ a strategy that should work:

- Amy has a copy of the Yeast Genome Tilling plasmid collection. I obtained plasmid YPGM11114 which contains the GAL1-10-7 genomic region

YPGM11114 INSERT:

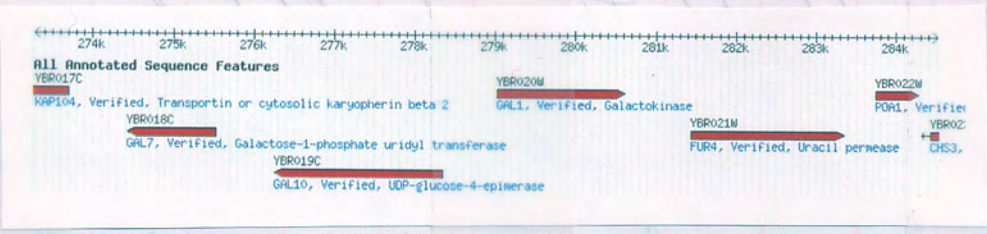

digested using NEB Cutter to find RE sites

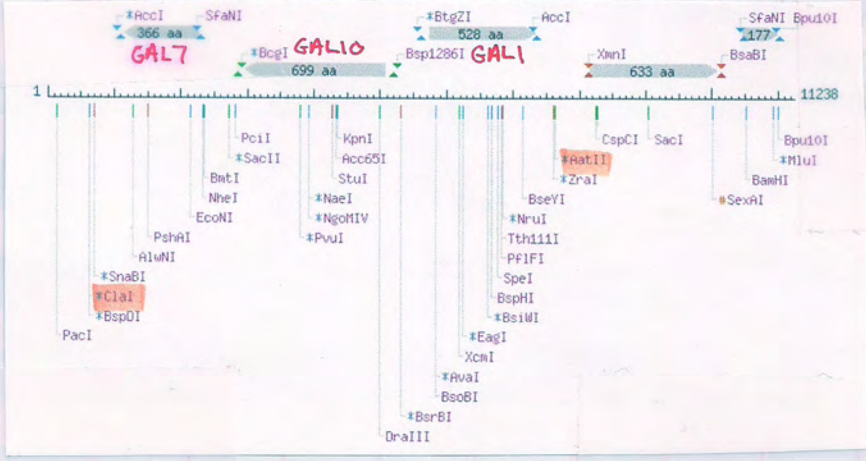

↳ AatII ~ 300 bp downstream of GAL1
ClaI ~ 500 bp downstream of GAL7

3/9/09

Strategy for integrating GAL1,10,7 @ YEL014C

· YEL014C is a dubious ORF that does not overlap w/ any other CDS. ∴ it is a suitable site to integrate GAL1,10,7.

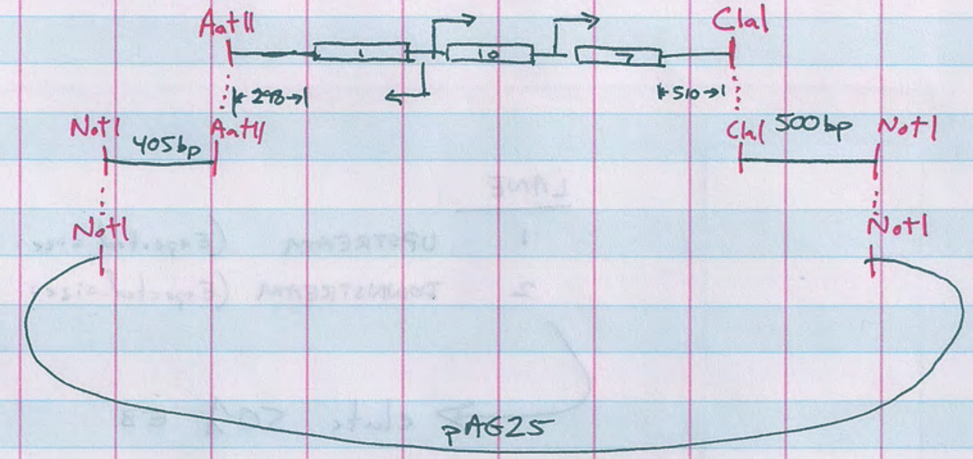

① Cut YPGM11114 w/ AatII & ClaI
② Ligate 405 bp upstream of YEL014C & 500 bp downstream of YEL014C using AatII & ClaI& sites, respectively
③ Cut w/ NotI and integrate into NotI sites of pAG25

· Over the weekend, I miniprepped 4 clones each containing YPGM11114 or pAG25:

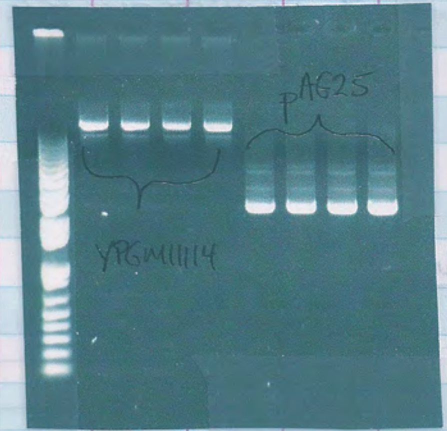

→ elute in 50λ EB

3/9/09

· PCR YEL014C UPSTREAM & DOWNSTREAM sequence using primers:

oGIL125	YEL014C_extF4_Not1	GTGGATGCGGCCGCTGTAATAATATTAAGATGGG
oGIL130	YEL014C_extR4_AatII	TAGTGCGACGTCTGTGATCAAATTTTTGTGTG
oGIL131	YEL014C_extF6_ClaI	TATCCAATCGATGCTAAAGTAATTCTTGGTTT
oGIL132	YEL014C_extR6_Not1	ATAGTCGCGGCCGCGCCATGCAGGGATATGACTG

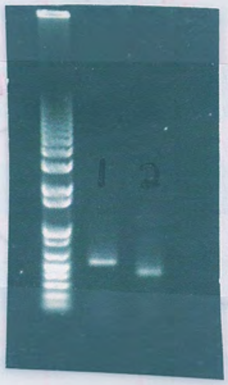

LANE
1 UPSTREAM (Expected size: 500 bp)
2 DOWNSTREAM (Expected size: 405 bp)

→ elute 50λ EB

Cla I & AatII DIGESTIONS

Digest the 4 YPGM11114 minipreps & the UPSTREAM & DOWNSTREAM PCRs
- add 5 λ NEBuffer 4 to each tube
- add 0.5 λ BSA to each
- add 0.5 λ AatII to the minipreps & UPSTREAM PCR
- add 0.5 λ ClaI to the minipreps & DOWNSTREAM PCR
- 37° block ~ 5pm.

3/10/09

Run out digests (~10 am)

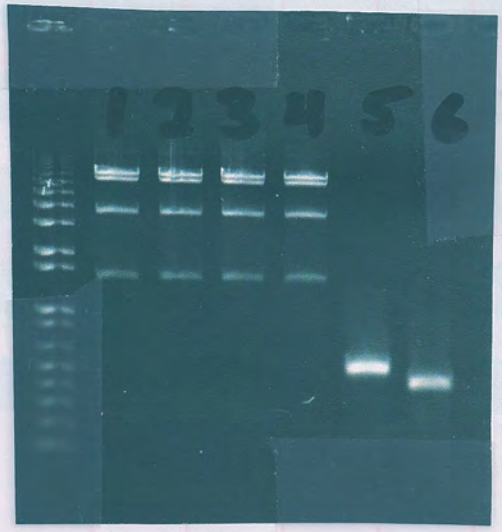

LANE
1 YPGM11114 A cut w/ AatII & ClaI
2 —"— B —"—
3 —"— C —"—
4 —"— D —"—
5 YEL014C UPSTREAM cut w/ AatII
6 YEL014C DOWNSTREAM cut w/ ClaI

3/10/09

Expected sizes for AatII & ClaI digest

The YPGM11114 insert could be in two orientations since it was cloned into one RE site.

ORIENTATION 1		ORIENTATION 2	
w/o Methylation	w/ Dam Methylation	w/o Methylation	w/ Dam Methylation
3229 bp }	6254*	3229 bp }	6254*
3025		3025	
1506	1506	4485	4485
6989	6989 ← GAL1,7,10	6989	6989
3653	3653	674	674

* one ClaI site is subjected to Dam methylation

- The digest on p. 90 is consistent w/ Orientation 1 + methylation
- The E. coli DH10B must be Dam-positive.

- PCR purify the UPSTREAM & DOWNSTREAM digests
- Gel purify the 6989 bp band

NEXT PAGE

⟶ I pooled the 4 YPGM11114 digests and ran them in 3 large lanes

---

- Overnight I attempted to PCR the GAL1,10,7 region from a WT FY4 strain (bmlΔ gDNA from freezer) and from the triple Δ (yGIL366 from benchtop). I used Primestar polymerase (previous run w/ Taq failed) and the following primers:

400downGALrevR: 5'AAAGGGTTCTGCTAGAGTCC3' (from Sandy)

&

44129480 IDT
G.LANG
83663969 3/4/2009
GAL7_extR2
5'-AAC ATA GGT GCA GGA TTT CC-3'
Tm= 52.5 °C , MW= 6,141.0
8.3OD260 41.60nmol 0.26mg

LANE	TEMPLATE
1	FY4 WT GAL1-10-7
2	yGIL366

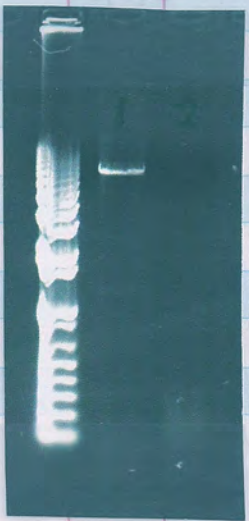

3/10/09

- Elute UPSTREAM & DOWNSTREAM into 30λ EB
- ~~B~~ Gel purify and pool plasmid digest band 7kb.
  ↳ Elute in combined 30λ

Check concentration by nanodrop:

UPSTREAM PCR	46.5 ng/μL
DOWNSTREAM PCR	30.4 ↓
GAL1·10·7	12.0

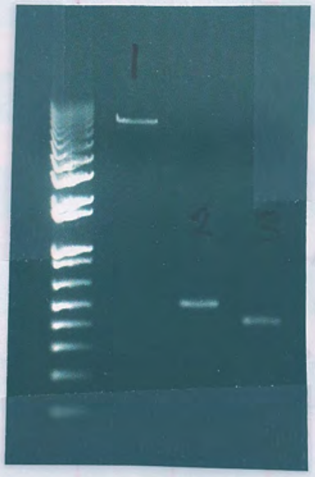

LANE	
1	GAL1·10·7, gel purified
2	UPSTREAM
3	DOWNSTREAM

Set up Ligation:

21.5 λ	GAL1·10·7
2.0 λ	UPSTREAM
2.0 λ	DOWNSTREAM
3.0 λ	10× Ligase Buffer
1.5 λ	T4 DNA ligase
30.0 λ	

RT Ligation @ 5:30pm

3/11/09

Run out ligation (1pm):

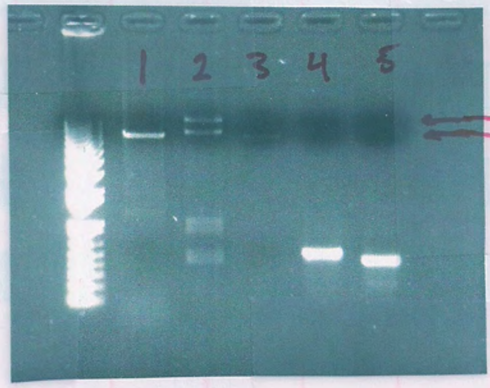

GAL·1·10·7 w/ ligated UPSTREAM/DOWNSTREAM ends

GAL·1·10·7

LANE	
1	GAL1·10·7 PCR (p.91)
2	Ligation
3	GAL1·10·7, gel purified
4	UPSTREAM
5	DOWNSTREAM

3/11/09

- Heat kill o/N ligation 10 min @ 65°
- PCR purify ligation ⟶ elute in 30 λ

- Cut insert & pAG25 w/ NotI

30 λ	pAG25 (A on p. 89, lane 1) or GAL1·10·7 ~~of~~ ligation
3 λ	NEBuffer 3
0.3 λ	100× BSA
0.3 λ	NotI
33.6 λ	⟶ 37° block @ 2:30

- At 5:45 - add 0.3 λ CIP to pAG25
- CIP for 1 hour
- Inactivate NotI ~ 20 min @ 65°C
- Put in freezer

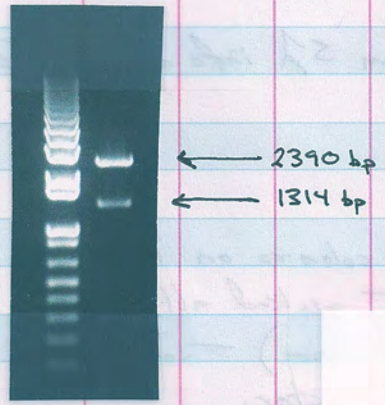

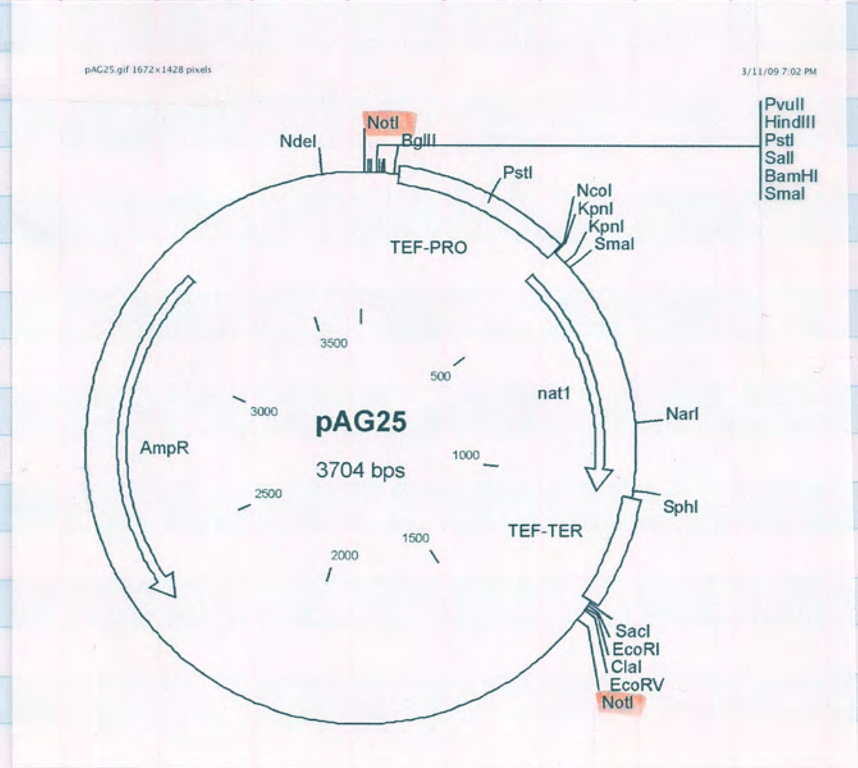

3/12/09

- Gel purify ligated GAL1·10·7 & YEL014C-sequence and pAG25 both cut w/ NotI ⟶ elute in 30 λ EB
  - I also gel purified the band of GAL1·10·7 w/o the YEL014C ends

Nanodrop to quantify:

vector	–	44.4 ng/μl
insert (GAL1·10·7 w/ ends)	–	1.8
GAL1·10·7 w/o ends	–	5.4

- Set up ligation:

16.5 λ	Insert (GAL1,10,7 w/ ends)	⟶	29.7 ng
0.5 λ	Vector	⟶	22.2 ng
2.0 λ	10× ligase buffer		
1.0 λ	T4 DNA ligase		
20.0 λ			

↳ ligate RT 20 min. ⟶ transform 5λ into duplicate DH5α

3/13/09

- Transformation efficiency was poor — only 3 colonies on Plate 1 ~~and~~ 5 on plate 2. This morning I picked all 8 and inoculated 2.5 mL LB+Amp (~10am) — I am concerned they may all be empty vector.

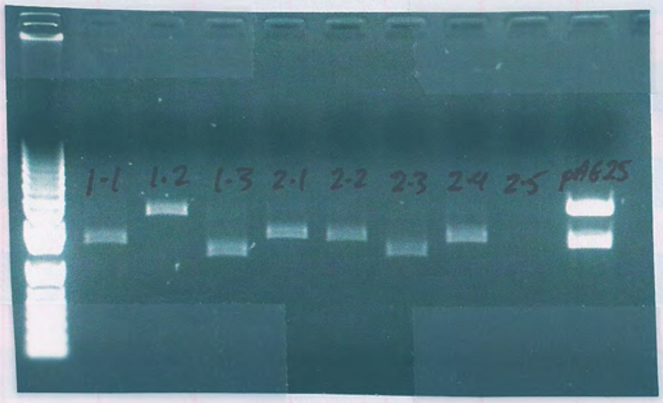

⟶ NotI digested minipreps

3/24/09

SECOND ATTEMPT AT MOVING GAL1·10·7

Last week I made midipreps of 2 clones each of YPGM11114 and pAG25 according to QIAGENs instructions.

→ Nanodrop to quantify

YPGM11114	A	261.2 ng/μL
"	B	257.6
pAG25	A	515.9
"	B	525.3
pAG25 miniprep (p.89)		88.1

· Digest YPGM11114

43.5 λ	Midiprep of YPGM11114
5.0	NEBuffer 4
0.5	BSA
0.5	AatII
0.5	ClaI
50 λ	

} Digest ~3h; heat inactivate enzymes @ 65° for 20min

· PCR upstream & Downstream linkers
→ · same as p.90 but 5, 100λ PCRs
· PCR cleanup pooled PCRs → elute 30λ EB
· Digest – add 3λ NEBuffer 4, 0.3λ BSA, & 0.3λ enzyme
→ AatII – upstream
ClaI – downstream

· Digest ~3 h, heat inactivate @ 65° 20min.

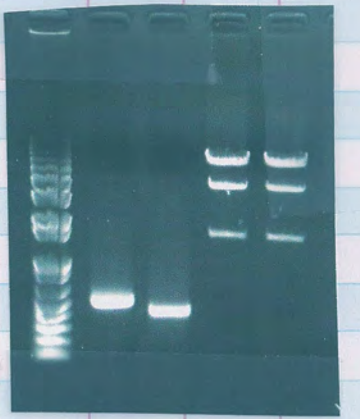

Lane
1 Upstream cut
2 Downstream cut
3 YPGM11114 A cut
4 " B cut

} the two ↑ MW bands can be distinguished if the gel is run out longer.

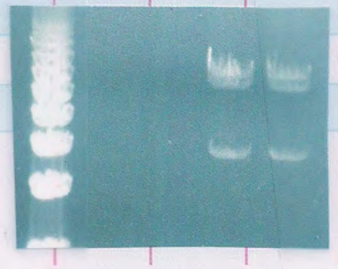

3/24/09

- Ligate linkers and cut plasmid

50 λ	YPGM11114 A cut w/ AatII & ClaI
30 λ	Upstream linker cut w/ AatII
30 λ	Downstream linker cut w/ ClaI
13 λ	10x Ligase Buffer
6.5 λ	T4 DNA ligase
~130 λ	

↳ Ligate ~ 1h @ RT

- P/C, ethanol precipitate ligated plasmid
  - bring vol up to 200 λ w/ TE
  - add 200 λ P/C, vortex, spin 5 min
  - transfer to new tube
  - add 25 λ 3M NaOAc
  - add 225 λ Isopropanol
  - ppt @ RT for 3.5h
  - spin 10 min ; aspirate
  - wash w/ 500 λ 70% EtOH; spin 2m; ~~Ast~~ aspirate; speed Vac
  - resuspend 50 λ TE
  - Nanodrop: 406.8 ng/μl

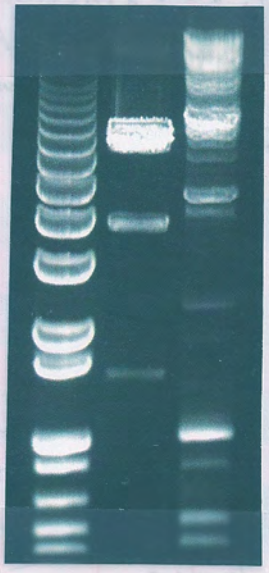

3/24/09

Transform yGIL413 & yGIL414 w/ ligatron

- Yesterday I started o/N cultures of yGIL413 & yGIL414
- This morning I diluted the cultures 1:100 into 10 ml YPD
- Coulter Count (2:30 pm)

Bkg	246000	146000
yGIL413	2.587E7	2.618E7
yGIL414	2.537E7	2.544E7
Post Bkg	914000	670000

- Start transformations ~ 2:45 pm

3/25/09

~~Replica plate~~ Yesterday I transformed both yGIL413 & yGIL414 with 10 λ of the ligatron on p. 96. As usual this was done in duplicate → one was plated onto YPD and the other onto YPD + ClonNat, G418, & Hyg. Today I see growth on both plates, with less growth near the center of the drug plate.

- Replica plate the 4 plates to minimal Galactose.

3/27/09

There are a large # of colonies on all 4 plates ~ probably between 500-1000 colonies. It is hard to tell if there are more colonies on the plates that were replica plated from YPD compared to the ones from YPD+drug.

I picked 16 colonies off of the yGIL413 & yGIL414 plates (from drug) ~~onto~~ → and restreaked onto YPD.

I overlaid drug onto YPD plates

G418	60 λ	+ 40 λ
ClonNat	30 λ	+ 70 λ
Hyg.	7.8 λ	+ 90.2 λ (new stock ~ 1140 mg/ml)
	↑ Drug	↑ H_2O

3/3~~0~~/09

VERIFYING TRANSFORMANTS

- On Sunday (3/29), I picked a single colony off of each of the 32 restreaked plates and inoculated 3 ml YPD.
- Yesterday I transferred 200λ of each ~~and replica~~ into a 96 well plate and pin transferred to YPD, ~~YPGal~~, minimal Gal, G418, Hyg, & ClonNat

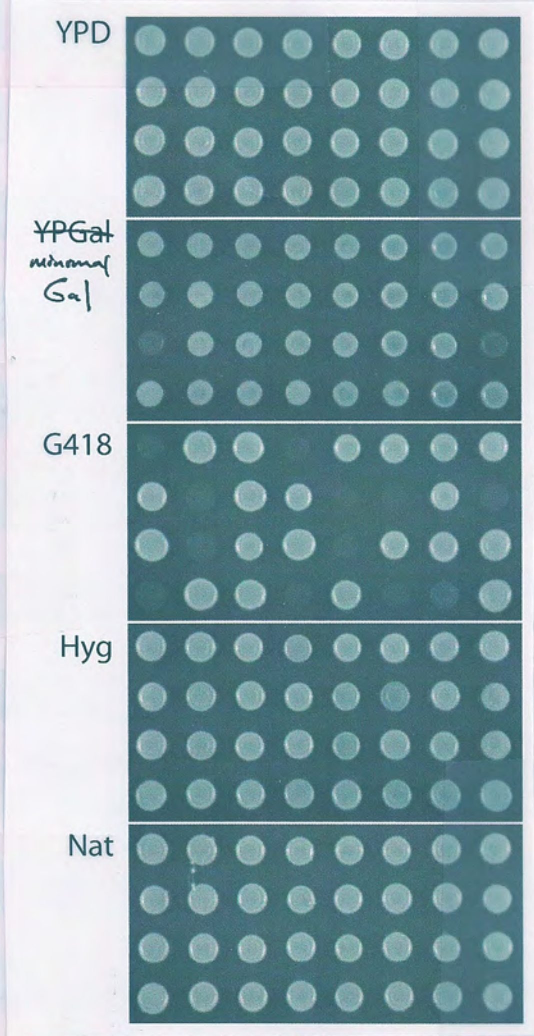

} putative yGIL413 transformants A → H, I → P

} putative yGIL413 transformants A → H, I → P

Notes

- As expected everything grows on YPD and YPD + ClonNat (Nat also marks the fluorophore)
- Two strains do not grow on GAL (~~413~~ 414 A & 414 H)
- 12 of the 32 putative transformants do not grow on YPD + G418 suggesting that GAL1.10.7 has integrated at its endogenous locus, thus replacing the drug cassettes.
- I expected the YPD + Hyg plate to look like the YPD + G418 plate, but everything grows → two possibilities
  - (1) not enough drug — this is the 1st time I used this Hyg. I should streak a sensitive strain onto this plate.
  - (2) These strains contain a duplication in the GAL1.10.7 region which duplicates the Hyg^R cassette. This could also explain my inability to generate a PCR product over this region (p. 91). I hope this is not the case.

4/2/09

I streaked two Hyg^S strains, yGIL213 & yGIL220 onto the Hyg plate → both grew

∴ Not enough drug.

3/31/09

I wanted to PCR verify that the GAL1·10·7 region integrated into YEL014C using the following primers

44504448 IDT
G.LANG
84023920 3/25/2009
YEL014C_extF7 135
5'- TAT TAA CGG CCA AAT TAC CC -3'
Tm= 50.3 °C , MW= 6,045.0
8.3OD260 42.70nmol 0.26mg

44504449 IDT
G.LANG
84023921 3/25/2009
YEL014C_extR7 136
5'- CAA AAT GAA CTA CAG GAT TCC -3'
Tm= 49.1 °C , MW= 6,407.2
8.6OD260 40.40nmol 0.26mg

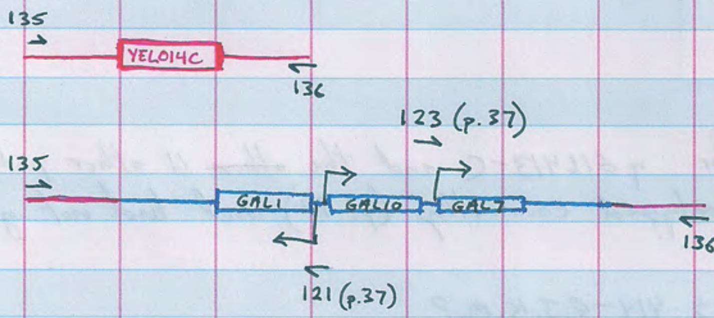

- I made gDNA preps of 12 putative transformants 413A-F & 414A-F
- PCR using:
  - YEL014C_extF7 / GAL1_extF3
  - YEL014C_extR7 / GAL7_extF3
  - YEL014C_extF7 / YEL014C_extR7

  } 3m. extension

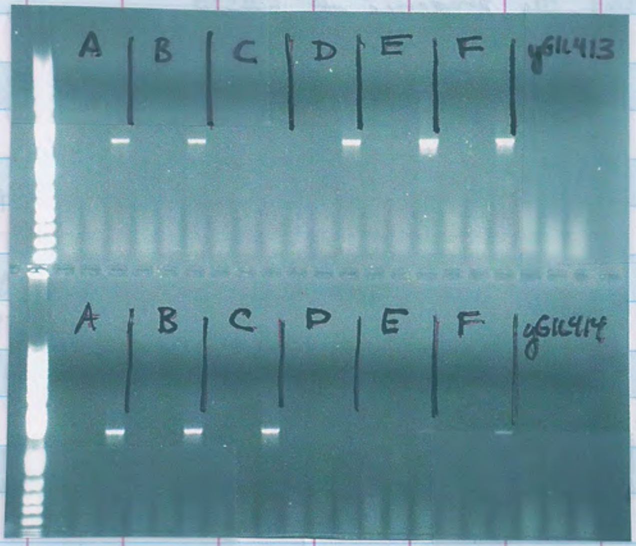

→ In each putative transformant, the three rows correspond to the three PCRs

NOTES
- Of these 12 strains, 7 phenotyped correctly (p 98).
- Only for 413C & 414D did I not get a PCR product for in-tact YEL014C
- I did not get any PCRs off of the parental gDNA preps
- I did not get PCR products w/ the check primers

- Rerun PCR using 413C & 414D as template and using primers GAL1_extF2 (p. 25) #113 and GAL7_extF2 (p. 25) #117 in place of GAL1-extF3 & GAL7-extF3

4/2/09

- For the two gDNA preps (413-C & 414-D) where I did not get any PCR products, I reran the PCRs:

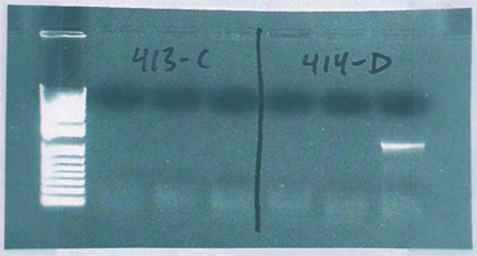

→ still nothing for 413-C, but 414-D is negative (lanes same as p. 99)

- I made gDNA preps for yGIL413-C and the ~~other~~ 11 other putative transformants that phenotyped correctly (p. 98) and had not yet been genotyped:

  413 - G, H, I, K, L, O & 414 - G, J, K, M, P

- PCR using primer pairs:

LANE 1	135 & ~~[illegible]~~ 113
" 2	117 & 136
" 3	135 & 136
" 4	115 & 116 (p 25) — verifies the presence of GAL10

(lanes 1–3: same as before)

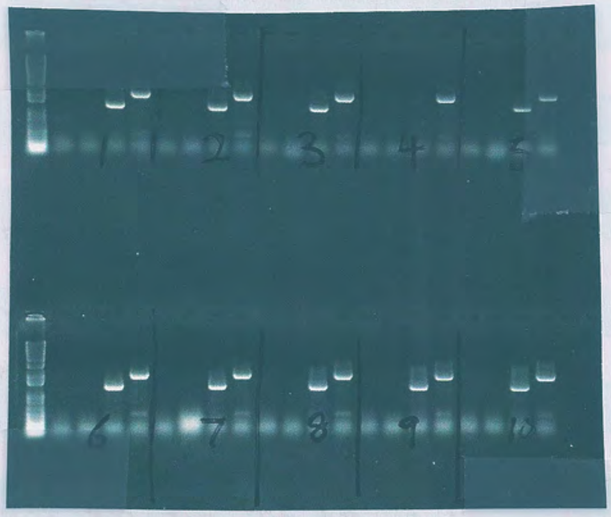

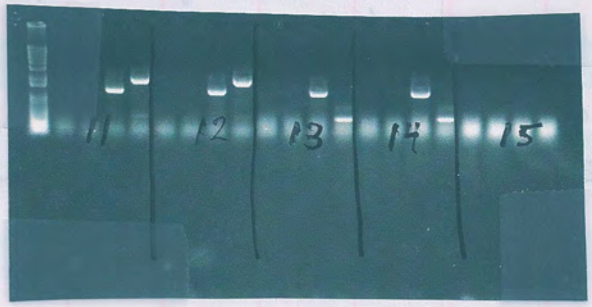

TEMPLATE

1	413 - C
2	↓ G
3	H
4	I
5	K
6	L
7	O
8	414 - G
9	↓ J
10	K
11	M
12	P
13	yGIL366
14	" 413
15	" 414

NOTES

- Perhaps still too much template DNA in yGIL414.
- As expected, no GAL10 in yGIL366 or yGIL413
- GAL10 present in everything else
- WT YEL014C in all strains (but ambiguous in 413-I)

→ will redo PCR on 413-I

4/2/09

CROSSING PHENOTYPICALLY CORRECT PUTATIVE TRANSFORMANTS

- 18 putative transformants are phenotypically correct:
  413 - B, C, E, F, G, H, I, K, L, O & 414 - C, D, F, G, J, K, M, P
  ↳ none genotyped correctly (413-I is still ambiguous)

Possibilities:
- I may have put YPGM11114 (or something like it) back together and the GAL.1.10.7 region is maintained as a 2 micron.
  ↳ there are a number of high MW bands on p. 96
- Other homology could have targeted GAL.1.10.7 to either LEU2 or regions adjacent to the endogenous GAL.1.10.7
- successful transformants could have resulted from duplication of the drug-marked region in these strains.

- To sort out the above possibilities, I crossed the above putative transformants to yGIL405 or yGIL407 depending on mating type. These strains have a WT GAL10 and are $G418^S$, but lack GAL1 and GAL7 therefore cannot use galactose.
  ↳ a useable strain will segregate $G418^R$ and Gal^+ 2:2 and independently

4/5/09

- On Wednesday evening (4/3) I picked a single diploid and inoculated 3 mL YPD.
- Today I took ~1.5 mL of the saturated culture, spun, washed w/ H_2O and resuspended in 4 mL 2% KAc.

- Since I am concerned that most of my putative transformants contain a plasmid I have been attempting to cure the plasmid by replica plating the triple-drug plates or the Gal plates (replica plated from the 3 drug) on YPD. I replica plated each twice to YPD (on 4/3 and 4/4). Today I replica plated to minimal Gal.
